# Genomic Characteristics of Recently Recognized Vibrio cholerae El Tor Lineages Associated with Cholera in Bangladesh, 1991 to 2017

**DOI:** 10.1128/spectrum.00391-22

**Published:** 2022-03-22

**Authors:** Md Mamun Monir, Talal Hossain, Masatomo Morita, Makoto Ohnishi, Fatema-Tuz Johura, Marzia Sultana, Shirajum Monira, Tahmeed Ahmed, Nicholas Thomson, Haruo Watanabe, Anwar Huq, Rita R. Colwell, Kimberley Seed, Munirul Alam

**Affiliations:** a icddr, b, Formerly International Centre for Diarrhoeal Disease Research, Bangladesh, Dhaka, Bangladesh; b National Institutes of Infectious Diseases (NIID), Tokyo, Japan; c Sanger Institute, Cambridge, United Kingdom; d Maryland Pathogen Research Institute, University of Maryland, Baltimore, Maryland, USA; e Bloomberg School of Public Health, Johns Hopkins University, Baltimore, Maryland, USA; f University of California, Berkeley, California, USA; University at Albany, State University of New York

**Keywords:** bacterial evolution, comparative genomics, *Vibrio cholerae* lineages, antimicrobial resistance, phage-inducible chromosomal island-like elements (PLE)

## Abstract

Comparative genomic analysis of Vibrio cholerae El Tor associated with endemic cholera in Asia revealed two distinct lineages, one dominant in Bangladesh and the other in India. An in-depth whole-genome study of V. cholerae El Tor strains isolated during endemic cholera in Bangladesh (1991 to 2017) included reference genome sequence data obtained online. Core genome phylogeny established using single nucleotide polymorphisms (SNPs) showed V. cholerae El Tor strains comprised two lineages, BD-1 and BD-2, which, according to Bayesian phylodynamic analysis, originated from paraphyletic group BD-0 around 1981. BD-1 and BD-2 lineages overlapped temporally but were negatively associated as causative agents of cholera during 2004 to 2017. Genome-wide association study (GWAS) revealed 140 SNPs and 31 indels, resulting in gene alleles unique to BD-1 and BD-2. Regression analysis of root to tip distance and year of isolation indicated early BD-0 strains at the base, whereas BD-1 and BD-2 subsequently emerged and progressed by accumulating SNPs. Pangenome analysis provided evidence of gene acquisition by both BD-1 and BD-2, of which six crucial proteins of known function were predominant in BD-2. BD-1 and BD-2 diverged and have distinctively different genomic traits, namely, heterogeneity in VSP-2, VPI-1, mobile elements, toxin encoding elements, and total gene abundance. In addition, the observed phage-inducible chromosomal island-like element (PLE1), and SXT ICE elements (ICE^TET^) in BD-2 presumably provided a fitness advantage for the lineage to outcompete BD-1 as the etiological agent of endemic cholera in Bangladesh, with implications for global cholera epidemiology.

**IMPORTANCE** Cholera is a global disease with specific reference to the Bay of Bengal Ganges Delta where Vibrio cholerae O1 El Tor, the causative agent of the disease showed two circulating lineages, one dominant in Bangladesh and the other in India. Results of an in-depth genomic study of V. cholerae associated with endemic cholera during the past 27 years (1991 to 2017) indicate emergence and succession of the two lineages, BD-1 and BD-2, arising from a common ancestral paraphyletic group, BD-0, comprising the early strains and short-term evolution of the bacterium in Bangladesh. Among the two V. cholerae lineages, BD-2 supersedes BD-1 and is predominant in the most recent endemic cholera in Bangladesh. The BD-2 lineage contained significantly more SNPs and indels, and showed richness in gene abundance, including antimicrobial resistance genes, gene cassettes, and PLE to fight against bacteriophage infection, acquired over time. These findings have important epidemic implications on a global scale.

## INTRODUCTION

Cholera is a life-threatening infectious diarrheal disease caused by Vibrio cholerae serogroups O1 and O139 of the Gram-negative gammaproteobacteria (1, 2). The global incidence of cholera is estimated to be 2.9 million cases annually with almost 95,000 deaths (3). In 2017, 34 countries reported a total of 1,227,391 cases and 5,654 deaths (4). Seven cholera pandemics have been recognized since 1817. However, limited information is available regarding the etiological agent for the first five pandemics and no isolates of the causative agent are extant. The sixth pandemic, and possibly those earlier were caused by V. cholerae O1 classical biotype, while the ongoing seventh pandemic is caused by V. cholerae El Tor biotype and began with the displacement of V. cholerae classical biotype in Asia in 1961 (5). V. cholerae El Tor was isolated in Africa in the 1970s and Latin America in 1991 where for more than a century there had been no cholera outbreaks (6). In 1992, a V. cholerae non-O1 strain designated V. cholerae O139 Bengal initiated outbreaks of cholera in coastal areas of India and Bangladesh and subsequently was isolated from patients in several countries of Asia (2). V. cholerae El Tor continues to be the major etiological agent of cholera worldwide.

The severe dehydrating diarrhea characteristic of cholera is associated with several factors, including a toxin and several virulence genes involved in colonization and toxicity and their coordinated expression (1). Cholera toxin (CT) is the virulence factor responsible for secretory diarrhea of cholera and is encoded in the genome of a lysogenic CTX phage. V. cholerae El Tor responsible for the current cholera pandemic harbors the CTX phage classical biotype variant, and the ctxB^cla^: ctxB genotype 1 (*ctxB*1) or *ctxB*7 (7). V. cholerae responsible for the current cholera pandemic has become more virulent by undergoing several shifts in CTX genotype and acquiring virulence-related gene islands (8). Integrative conjugative elements (ICEs) and lysogenic phages are genetic elements that play an important role in the acquisition of virulence, antimicrobial resistance, and heavy metal resistance, which are important components of the pathogenicity of V. cholerae (9, 10). Functions of these elements are important for the pathogen to exert evolutionary advantage and variants can be used as markers of clonal expansion (1). Acquisition of mobile genetic elements (MGEs) through horizontal gene transfer (HGT) and propitious chromosomal mutations are significant landmarks for an evolving bacterium (11).

Whole-genome sequencing of V. cholerae El Tor strains associated with the seventh cholera pandemic revealed three waves, suggesting independent but overlapping paths for the pathogen to spread globally from the Bay of Bengal estuary where cholera has been endemic at least since 1961 but likely for centuries (5). Intercontinental transmission of V. cholerae has been proposed for the 2010 outbreak in Haiti (12). Bangladesh borders on the Bay of Bengal and is considered to be a hot spot of Asiatic cholera, where ca. 100,000 cases and 4,500 deaths are reported each year (13). V. cholerae O1 responsible for endemic cholera in Bangladesh and India has been found to have undergone genetic changes over time, including the acquisition of classical biotype attributes in an El Tor background, thereby becoming more successful as a pathogen (14, 15). A recent whole-genome analysis of Vibrio cholerae El Tor strains isolated between 2009 and 2016 indicated two distinct lineages exist in Bengal (16). The objective of the study reported here was to investigate V. cholerae endemic cholera strains isolated from 1991 to 2017 to understand more completely the emergence and progression of the two lineages in Bangladesh. Virulence and related genomic islands, including toxin and antimicrobial resistance genes differing significantly among the V. cholerae El Tor lineages, were also investigated for potential relevance to the emergence of the lineages.

## RESULTS

### Phylogenetic analysis.

**FIG 1:**
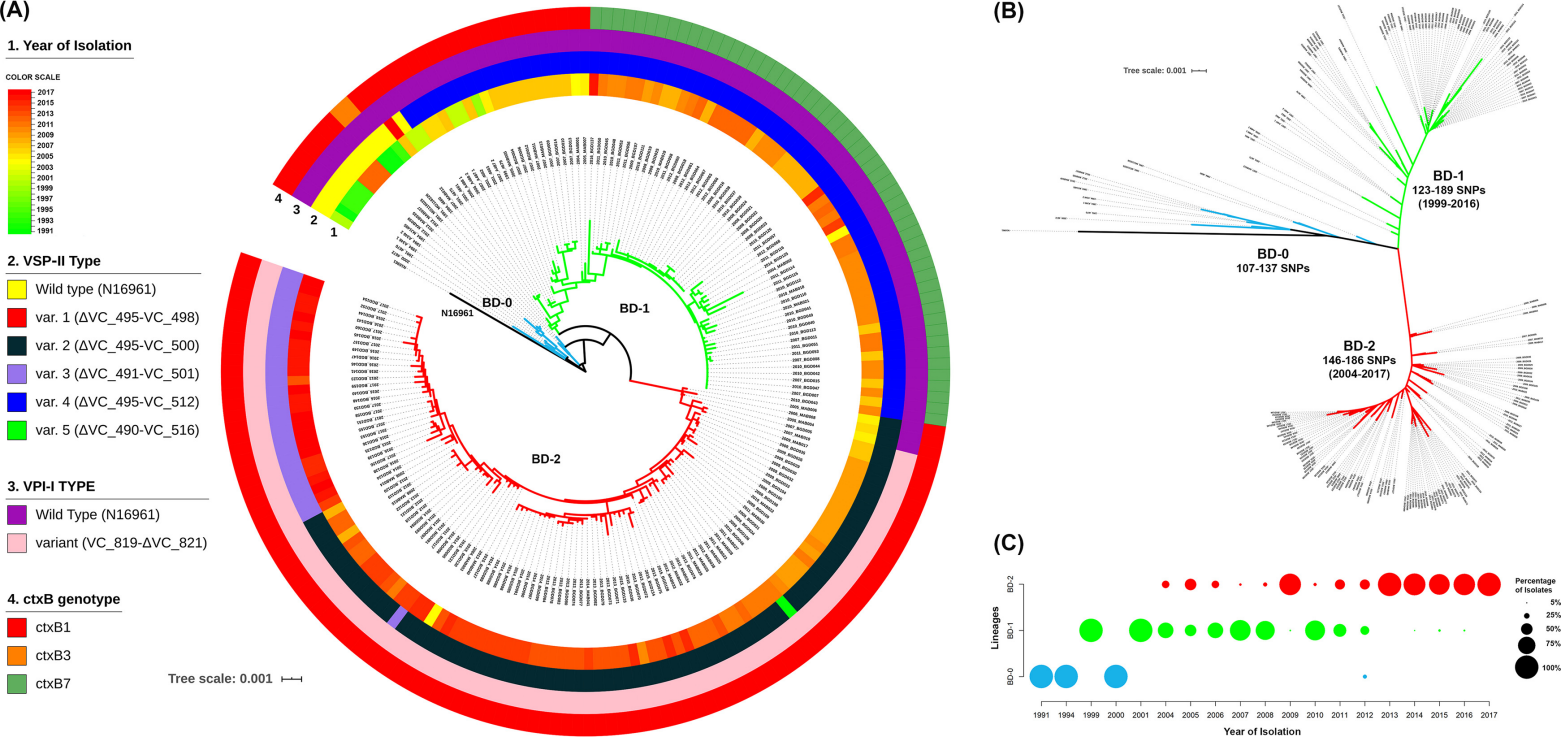
Phylogenetic analyses of strains showing respective genomic features and year of isolation. (A) Maximum likelihood phylogenetic tree generated from whole-genome SNPs and number of isolated V. cholerae O1 El Tor strains belonging to lineages BD-0, BD-1, and BD-2 rooted from out-group reference strain Vibrio cholerae N16961. Rings show features of the isolates according to the color scheme provided on the left. Tree branches are colored blue, green, and red defining lineages BD-0, BD-1, and BD-2, respectively. (B) Unrooted tree showing independent evolution of BD-1 and BD-2 strains with the number of core genome SNPs of strains in the lineages compared to the N16961 reference strain. (C) Percentage of isolates per year for the three lineages. The size of the circles indicates the percentage of strains belonging to lineages according to the scheme shown.

A total of 119 strains were included in the study and their genomes were sequenced using the Illumina platform (MiSeq or HiSeq 2500 sequencer). In addition, 56 strains from our previous study (16) and 17 genomes from the European Nucleotide Archive (17) were used, which are representative of isolates from Bangladesh between 1991 and 2017 (Table S1). Paired-end reads of the 192 genomes were mapped to V. cholerae El Tor N16961 reference strain, a seventh-pandemic V. cholerae O1 El Tor (7PET) strain isolated in Bangladesh in 1975 (18). A total of 1,298 single nucleotide polymorphisms (SNPs) and 413 indels (insertions or deletions) were obtained and, after filtering indels, low call rate, and high-density SNPs, a total of 893 high-quality SNPs were retained for further study. A phylogenetic analysis was conducted to construct a tree based on the 893 high-quality SNPs to evaluate the genetic diversity of the Vibrio cholerae O1 El Tor isolates from Bangladesh. A nested hierarchical structure in the phylogenetic tree was observed, with all but four of the strains isolated between 1999 and 2017 clustering into two major clades, BD-1 (*n* = 76) and BD-2 (*n* = 105), shown in green and red, respectively. The remaining strains formed paraphyletic group BD-0 (*n* = 11) (Fig. 1A, blue). Except for three strains isolated in 2012 that formed a subclade, BD-0 consisted mostly of strains isolated earlier between 1991 and 2000. Dates of isolation of common ancestors of the lineages were inferred using Bayesian Markov Chain Monte Carlo framework Bayesian evolutionary analysis sampling trees (BEAST) (19) (Fig. S1), and maximum clade credibility (MCC) tree was inferred from the posterior distribution of the best fitting model using program TreeAnnotator tool of the BEAST software package. It was estimated from the MCC tree that the most recent common ancestor (MRCA) of lineage BD-1 was isolated in 1987 (95% HPD: 1983 to 1991), and lineage BD-2 in 1997 (95% HPD: 1994 to 2000), where HPD stands for height posterior density. Strains of BD-1 and BD-2 shared genome sequences of strains isolated since 1981 (95% HPD: 1976 to 1986). The number of SNPs in strains of the two clades is relative to reference V. cholerae N16961, which showed strains of BD-0 differed by 107 to 137 SNPs, BD-1 by 123 to 189 SNPs, and BD-2 by 146 to 186 SNPs. An unrooted tree showed SNP diversity among BD-0, BD-1, and BD-2 clades with SNP diversity of BD-2 highest (Fig. 1B). Comparison of isolates in the clades and year of isolation revealed clonal aggregation within the dominant clade and strong temporal signature. Strains of BD-1 and BD-2 were found to be temporally spread but simultaneously isolated during the periods of 2004 to 2011, 2012, 2014 to 2016 (Fig. 1C, Table S2). Strains of BD-1 were mainly isolated during 2004 to 2011 (66.3%, *n* = 65) while strains of BD-2 were isolated during those years in fewer numbers (33.7%, *n* = 33) except 2009 when BD-2 strains were dominant (93.33%, *n* = 14) (Table S1). The following years, from 2012 to 2017, showed BD-2 strains to be dominant (73.5%, *n* = 72) and BD-1 strains the minority (10.2%, *n* = 10).

### Genetic variants associated with the clades.

**FIG 2:**
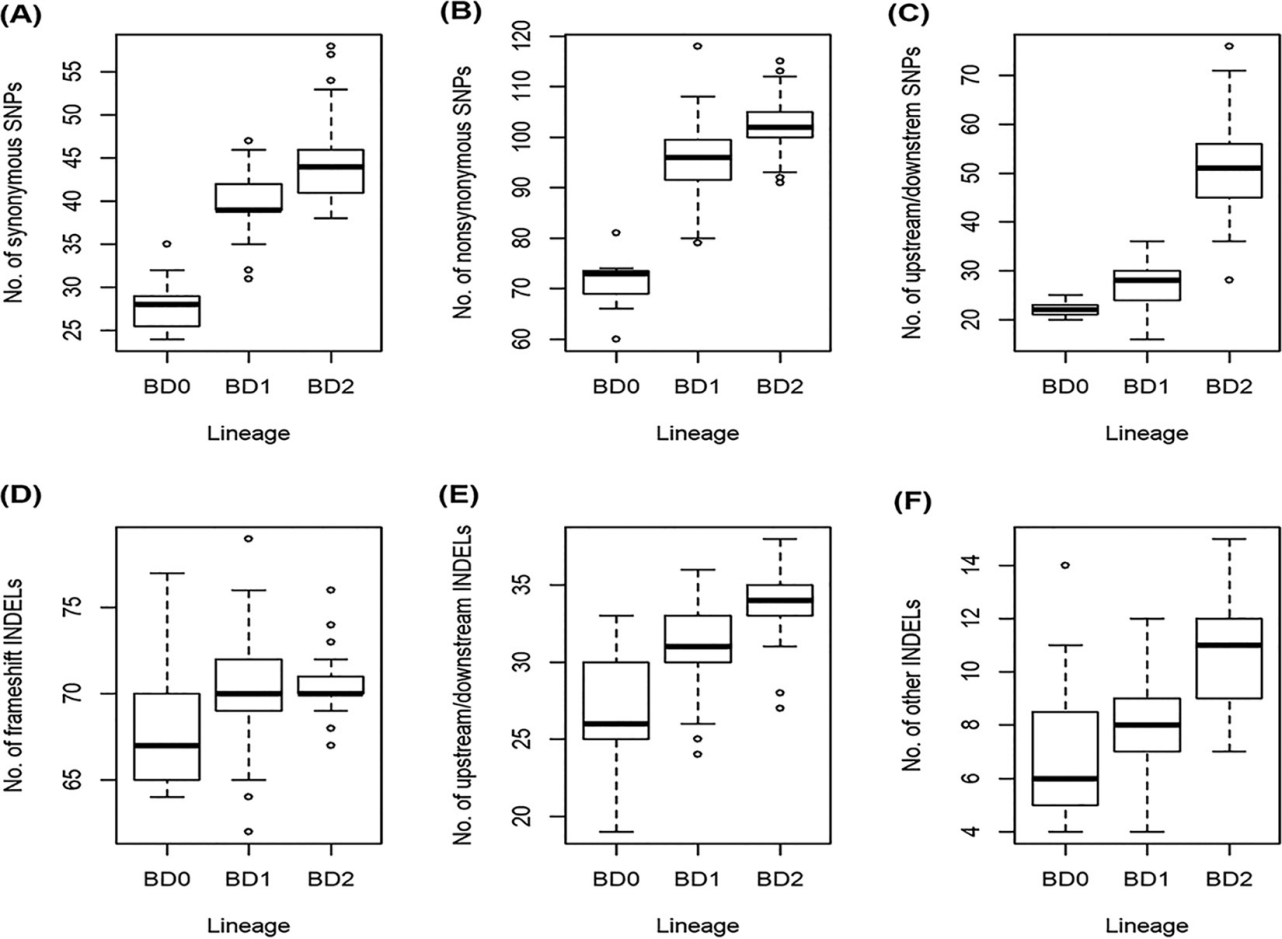
Box plots of SNPs distribution and indel type in each of three lineage groups. (A) Distribution of 337 synonymous SNP variants. This figure shows that strains of BD-2 lineage accumulated more synonymous SNP variants compared to BD-0 and BD-1 lineages. Notably, synonymous SNP variants do not change the form of protein. (B) Distribution of 613 nonsynonymous SNP variants. These non-synonymous SNP variants include 570 missense variants, 38 stop gained variants, 2 splice-region-variants and stop-retained-variants, 2 stop-lost and splice-region-variants, 1 initiator codon variant. (C) Distribution of 348 upstream/downstream SNP variants. (D) Distribution of 238 frameshift indel variants. (E) Distribution of 107 upstream/downstream indel variants. (F) Distribution of 68 indel variants, including 13 conservative-inframe-insertions, 14 disruptive-inframe-insertions, 11 frameshift-variant and stop-gained, 10 disruptive-inframe-deletions, 10 conservative-inframe-deletions, 1 stop-gained and disruptive-inframe-deletions, 2 feature-elongations, 1 frameshift-variant and stop-lost and splice-region-variant, 1 stop-gained and disruptive-inframe-insertion, 2 frameshift-variant and splice-region-variant, 2 frameshift-variant and start-lost, 1 stop-gained and conservative-inframe-insertion.

Associations between lineages and the genetic variants were studied using 1298 SNPs and 413 indels, identified by aligning raw reads against V. cholerae N16961 reference genome. Variant annotation using SnpEff (20) showed that among the 1298 SNPs, there were 337 synonymous, 613 nonsynonymous, and 348 variants on intergenic regions (Fig. 2A to C, Table S2). Moreover, of 413 indels, there were 238 frameshift-variants, 107 variants on intergenic regions, and 68 other types of variants (Fig. 2D to F, Table S2). Most of the identified SNPs and indels were in the protein-coding region, many of which function to change the form of a protein. By plotting the distribution of SNP types and indel variants for BD-0 (*n* = 11), BD-1 (*n* = 76), and BD-2 (*n* = 105), it was observed that strains of the clades accumulated SNPs and indels. Strains of BD-2 accumulated more SNPs and indels, increasing genetic distance from BD-0 and BD-1 (Fig. 1B, Fig. 2) and suggesting evolution was occurring compared with reference V. cholerae O1 N16961.

**FIG 3:**
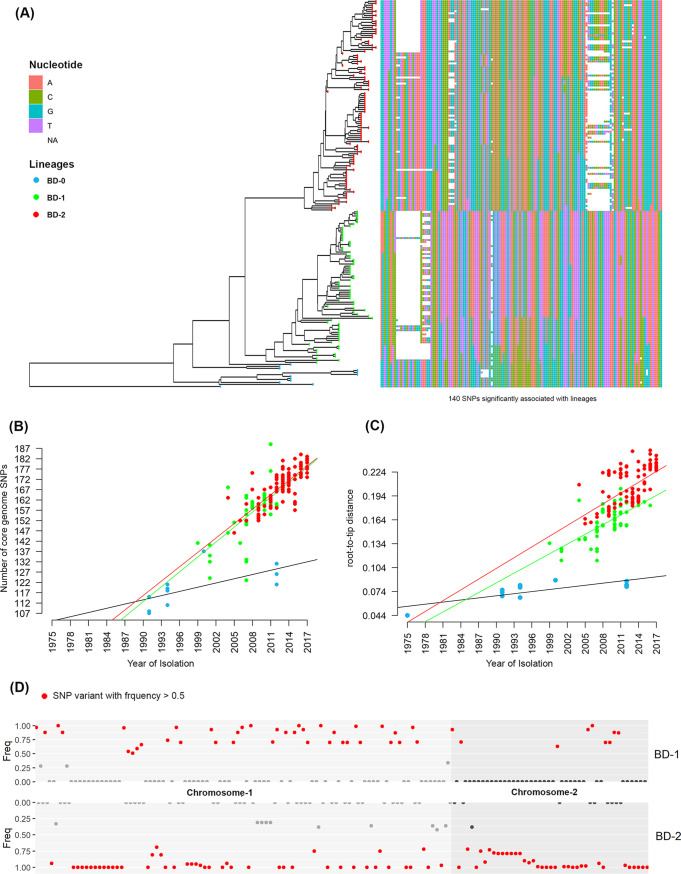
SNP analysis of genetic diversity. (A) Phylogenetic treemap of the strains and heat map for genotypes of 140 SNPs are significantly associated with different lineages. The colors delineate four different nucleotides where white represents the missing genotype. Heatmap shows clear differences in the lineages. (B) Number of core genome SNPs referencing the year of isolation. The figure shows the steady accumulation of SNPs of different lineage strains over time. (C) Regression analysis of root-to-tip distance for strains of the lineages. This figure shows the diversity of strains of different lineages. (D) Miami plot of alternative allele frequencies of SNPs for the dominant lineages BD-1 and BD-2. This figure shows the clear difference in SNP accumulation by the two dominant lineages BD-1 and BD-2.

Fisher exact test (21) was performed for association analysis between genetic variants and the clades BD-1 and BD-2. Association analysis showed that 140 SNPs and 31 indels had a genome-wide significant association (*P* < 6.40 × 10^−9^) with BD-1 and BD-2. Among the 140 SNPs were 25 synonymous variants, 53 missense variants, 2 stop gain variants, and 60 variants on intergenic regions (Table S3 and Fig. S2). It was discovered that 21 SNP missense mutations were present in genes with known functions in more than 80% of BD2 strains, resulting in mutant proteins (Table 1). However, there were only seven missense mutations were found in genes with known functions in more than 80% of BD1 strains. Genotype and frequency of 140 significantly associated SNPs, number of SNPs by year of isolation, and root to tip distance showed significant genetic differences between BD-1 and BD-2 (Fig. 3). The number of core genome SNPs by year of isolation was analyzed to detect temporal SNP accumulation patterns of the clades. The number of core genome SNPs did increase over time for both BD-1 and BD-2 (Fig. 3B). Moreover, root-to-tip regression analysis indicated a steady increase in SNP divergence among the strains of the two clades over time (Fig. 3C). Miami plot for frequency of alternative alleles of the 140 significant SNPs showed BD-2 strains had accumulated more clade-specific SNPs, notably in chromosome-2 compared to BD-1 (Fig. 3D).

**TABLE 1:** SNPs resulted unique mutant proteins in BD1 and BD2

SNP^*a*^	REF	ALT	FrqBD1	FrqBD2	*P* value	Gene	AA change	Product
S1_2609994	G	A	0	105	5.61E−53	nudF_1	Arg109Cys	ADP-ribose pyrophosphatase
S2_266019	A	G	0	105	5.61E−53	ulaA	Ile354Thr	Ascorbate-specific permease IIC component UlaA
S2_1024884	G	A	0	105	5.61E−53	putA	Ala600Val	Bifunctional protein PutA
S2_989172	C	T	0	105	5.61E−53	yecS	Pro191Ser	YecS
S1_798976	T	C	0	105	5.61E−53	suhB	Glu217Gly	Inositol-1-monophosphatase
S1_994229	G	A	0	105	5.61E−53	stcE_2	Gly201Asp	Metalloprotease StcE precursor
S2_921045	A	C	0	105	5.61E−53	ctpH_6	Ile161Ser	Methyl-accepting chemotaxis protein CtpH
S1_1622584	G	A	0	105	5.61E−53	cobB	Pro50Leu	NAD-dependent protein deacetylase
S2_773493	T	A	0	105	5.61E−53	phhA	Gln19Leu	Phenylalanine-4-hydroxylase
S1_681574	G	T	0	105	5.61E−53	glmM	Arg196Leu	Phosphoglucosamine mutase
S2_161094	T	G	0	105	5.61E−53	siaT_5	Ser241Ala	Sialic acid TRAP transporter permease protein SiaT
S1_1452755	T	C	0	105	5.61E−53	cysG_1	Val38Ala	Siroheme synthase
S1_2731709	G	A	0	105	5.61E−53	tamA	Thr266Ile	Translocation and assembly module TamA precursor
S1_545919	T	G	0	104	4.32E−51	pctB_1	Leu249Trp	Methyl-accepting chemotaxis protein PctB
S1_2814292	T	C	0	102	4.43E−48	argG	Thr283Ala	Argininosuccinate synthase
S1_1332186	T	G	0	99	1.96E−44	gyrA	Asp660Glu	DNA gyrase subunit A
S1_149686	G	T	0	99	1.96E−44	murI	Ala137Ser	Glutamate racemase
S2_562858	A	T	0	99	1.96E−44	VCA0627	Thr6Ser	rRNA methylase
S1_628646	C	T	0	85	1.32E−32	hrpB_1	Ala782Val	ATP-dependent RNA helicase HrpB
S1_673206	A	G	0	85	1.32E−32	tyrS_2	Thr393Ala	Tyrosine—tRNA ligase
S1_2357516	G	A	0	79	7.24E−29	angR	Leu227Phe	Anguibactin system regulator
S1_2483236	G	A	66	0	4.18E−39	lysX	Ala150Thr	Alpha-aminoadipate—LysW ligase LysX
S1_1682925	C	T	67	0	3.63E−40	appC	Ala226Thr	Cytochrome bd-II ubiquinol oxidase subunit 1
S1_368119	T	C	67	0	3.63E−40	mutL	Cys350Arg	DNA mismatch repair protein MutL
S1_1359179	G	A	67	0	3.63E−40	licH	Ala56Thr	putative 6-phospho-beta-glucosidase
S1_1060408	C	T	71	0	6.86E−45	nagA_1	Asp150Asn	N-acetylglucosamine-6-phosphate deacetylase
S1_276112	G	A	76	0	5.61E−53	mak	Gly116Arg	Fructokinase
S1_1782501	G	A	76	0	5.61E−53	cph2_4	Leu79Phe	Phytochrome-like protein cph2

a Here, SNP refers to the SNPs which had alternative alleles uniquely found in more than 80% of BD1 or BD-2 strains, located within proteins of known functions and altered amino acids. SNPs were named according to their chromosomal position. For example, “S1_2609994” is an SNP/indel site, where “S” stands for the site and “2609994” stands for the site's base pair location. Reference allele = REF, alternative allele = ALT, AA change = amino acid change. Freq_BD1 is the frequency of an alternative allele in BD1 and Freq_BD2 is the frequency of an alternative allele in BD2. Note that, the frequencies of alternative alleles of the SNPs are zero for BD-0. *P* value is from the Fisher exact test.

### Relative gene abundance.

**FIG 4:**
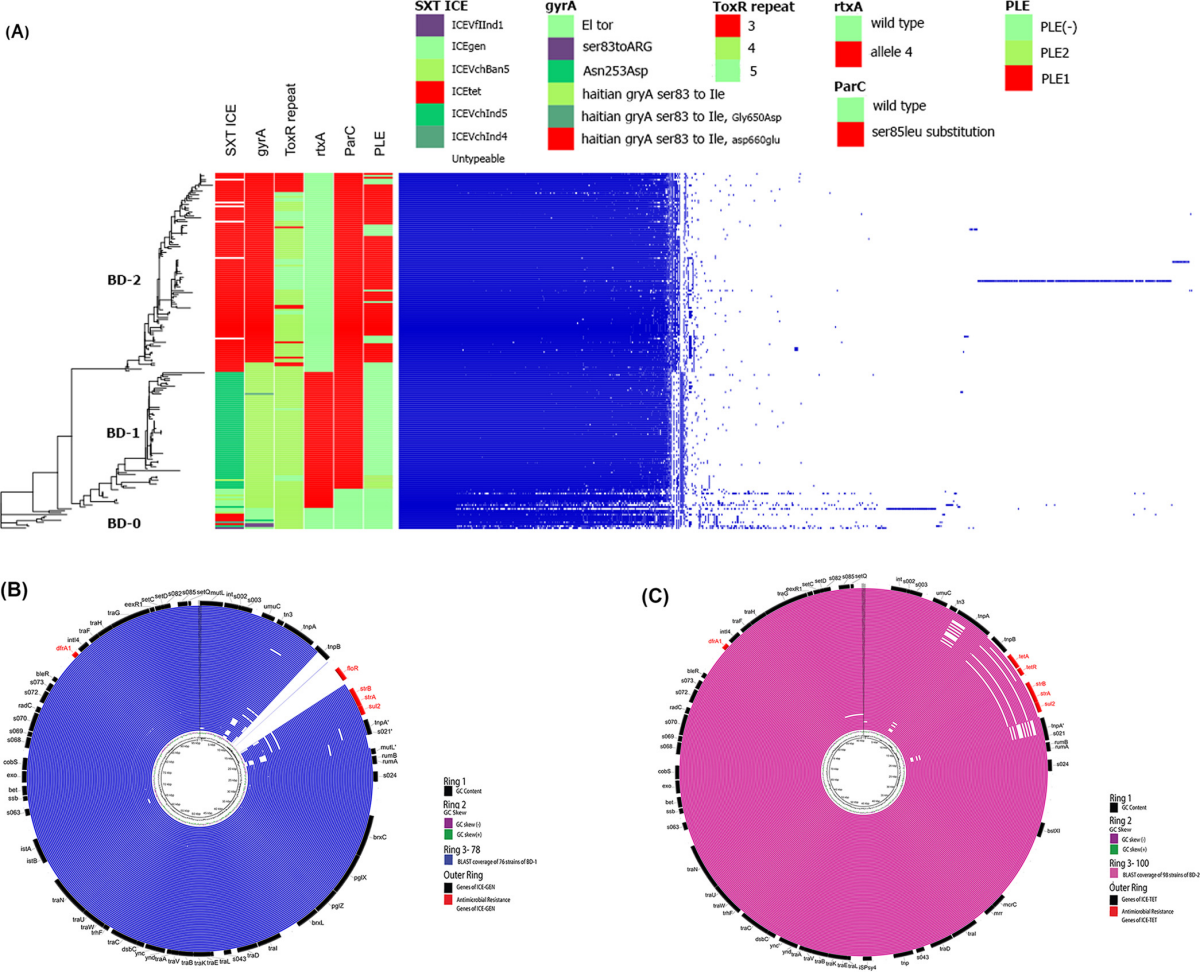
Pangenome analysis showing differences in the abundance of gene clusters among the lineages. (A) Relative gene abundance of lineages identified by Roary. Features of the sequences are shown with bars and details for features listed in Table S1. (B) BLAST coverage of SXT regions of BD-1 isolates compared with ICE-GEN. Rings represent sequentially outwards following Table S1. Outermost ring shows the different genes of ICE-GEN. (C) BLAST coverage of SXT regions of BD-2 isolates compared with ICE-TET. The rings represent strains of BD-2 sequentially outwards following Table S1. The outermost ring shows different genes of ICE-TET.

Pangenome analysis was done using Roary to investigate differences in core and pan genes among the strains of BD-0, BD-1, and BD-2. Roary classified the identified functional genes into four categories: (i) core genes, present in 99 to 100% of the strains; (ii) softcore genes, present in 95 to 99% of the strains; (iii) shell genes, present in 15 to 95% of the strains; and (iv) cloud genes, present in less than 15% of the strains (22). Pangenome analysis revealed significant differences in overall gene composition among the clades (Fig. 4A). According to the definition of core genes in pangenome analysis, the number of core genes largely varied among BD-0, BD-1, and BD-2 (Table S4). Similarly, the number of soft-core genes was also varied. BD-0 is a group of close relatives with a larger genetic distance relative to BD-1 and BD-2. All BD-0 strains and more than 95% of the BD-1 and BD-2 strains had 1102 common genes (Table S5A) most having a known function. About 10% of BD-2 strains had 44 unique genes of which six encoding crucial proteins of known function were found in more than 90% of the BD-2 strains. Those genes are tetracycline repressor protein (tetR), tetracycline resistance protein (tetA), type-I restriction enzyme EcoKI M protein (hsdM), type-I restriction enzyme EcoR124II R protein (hsdR), Mrr restriction system protein (mrr), and 5-methylcytosine-specific restriction enzyme B (mcrB) (Table S5B). In addition, methyl-accepting chemotaxis protein (CtpH) and group_10030 virulence proteins were exclusively found in 60% and 65% of BD-2 strains, respectively. In contrast, about 5 to 15% of the BD-1 strains carried 19 genes that were unique for them (Table S5C). Three genes common to all BD-0 strains were not detected in BD-2 and were present only in 1 to 2 of the BD-1 strains.

Next, we conducted Pan-GWAS to identify clade-specific genes by considering gene presence and absence as the explanatory variable and defined lineage groups as the response variable. A total of 92 genes were significantly (*P* < 4.98 × 10^−6^) associated with BD-0 and BD-1 (Table S6A). Of these, 62 genes were identified in 54 to 73% of BD-0 but not in BD-1 strains. Of 164 genes associated with BD-0 and BD-2, 46 were found in more than 73% of BD-2, but not in BD-0 strains (Table S6B). In addition, 66 genes were found in more than 45% of BD-0, but not in BD-2 strains. Of 143 genes associated with BD-1 and BD-2 (Table S6C), 29 were found in more than 76% of BD-1, but not in BD-2 strains. Again, 47 genes were found in 22 to 97% of the BD-2, but not in BD-1 strains. These results provide evidence that strains of BD-1 and BD-2 diverged and evolved as two lineages by accumulating genes, after originating from common ancestor BD-0.

### Pathogenicity islands and phage inducible chromosomal island-like elements.

**FIG 5:**
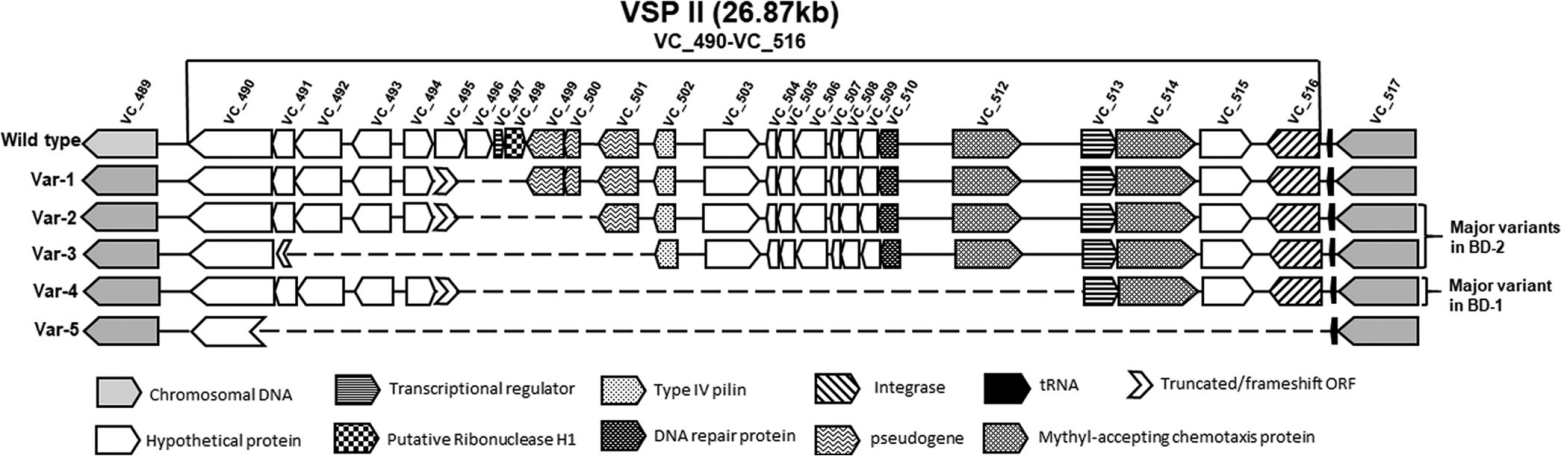
Schematic diagram of VSP-II. Schematic alignment view of VSP-II regions for the isolates. Direction of gene transcription is indicated by arrows and gene shadows represent functional annotation. Six types were identified with all BD-0 strains wild-type VSP-II. Two major types, var-2 and var-3, were observed for most BD-2 strains and one major type var-4 for most BD-1 strains.

V. cholerae strains included in this study were further examined by targeting the pandemic and pathogenicity islands namely, VSP-1, VSP-II, VPI-1, and VPI-2, including the phage inducible chromosomal island-like elements (PLE). Based on the extent of detected regions compared to V. cholerae N16961, five variants of VSP-II (variants 1 to 5 of the wild type) as reported in our recent study (16), and one variant of VPI-1 (variant 1 of the wild type) were observed (Fig. 5). V. cholerae El Tor strains differed in the type of VSP-II and VPI-1 variants. BD-0 had the wild-type of VSP-II, in reference to the El Tor N16961 strain. Most BD-1 strains (except two) had variant-4 VSP-II, with a partial deletion in VC_495 and complete deletion in VC_496 to VC_512, and BD-2 strains carried three VSP-II variants of which ca. 73% had variant-2 VSP-II with partial ORF VC_495 deletion, and complete VC_496 to VC_500 deletion, which appeared consistent with our prior study (16). BD-0 and BD-1 harbored wild type of VPI-1, whereas most of the BD-2 strains (102 of 105 strains) had variant VPI-1 with complete deletion of VC_819 to VC_820 ORFs; and partial deletion in VC_821. All BD-0 strains and 66 of 76 BD-1 strains lacked PLE (Table S1 and S7), while PLE2 was found in 10 BD-1 strains isolated in 2007 possessing the *ctxB*1 genotype, and one in 2005. Interestingly, most of the BD-2 strains (83 of 103) carried PLE1, but the rest lacked PLE. Thus, BD-2 lineage strains associated with recent Bangladesh endemic cholera are variant-3 VSP-II, variant VPI-1, and the majority possess PLE1.

### Variations in SXT/R391 and important genes.

Although differences in SXT/R391, *ctxB*, *gyrA*, *rtxA*, and *parC* across two lineages (BD-1, an analog of lineage-2; BD-2, an analog of lineage-1) were investigated in our recent study (16), these important genetic elements were rechecked to draw overall conclusions for all strains included in this investigation. Moreover, variation in ToxR binding repeats was checked across strains of different lineages. Integrative and conjugative elements (ICEs) were targeted from whole-genome sequences by aligning raw reads or contigs with five publicly available sequences of the ICE element (accession no. GQ463140.1, GQ463141.1, GQ463142.1, MK165649.1, and MK165650.1). Nucleotide blast was used to match extracted sequences with ICE element sequences and typed based on the highest bit score. Four strains of BD-0 blast search yielded high bit scores when aligned with ICE^GEN^ (MK165650.1), ICE*Vch*Ind5 (GQ463142.1), or ICE*Vch*Ban5 (GQ463140.1). Bit scores were highest for the other BD-0 strains when aligned with ICE^TET^ (accession no. MK165649.1), which has genomic characteristics similar to ICEVchVhn2255 (accession no. KT151660). For all BD-1 strain bit scores were high when aligned with ICE^GEN^, ICEVchInd5, or ICEVchBan5, and for BD-2 strains bit scores were highest when aligned with ICE^TET^, which is consistent with our previous results. All BD-1 and BD-2 strains contained mutant *gyrA* with an amino acid alteration Ser83Ile, whereas 99 (94.28%) of the 105 BD-2 strains exhibited Asp660Glu, which was not present in BD-1 or BD-0, also supporting our previous findings.

V. cholerae O1 El Tor strains in this study were CTX positive, and each carried a single copy of CTXФ with a particular *ctxB* genotype. Three variants, *ctxB1* (classical genotype), *ctxB3* (typical El Tor genotype), and *ctxB*7 (Haitian variant), of the cholera toxin gene, were detected and found associated with the clades (Fig. 1A). Similar to previous findings, all BD-2 strains had *ctxB1* genotype, majority of BD-1 strains had *ctxB7* genotype, and all but two BD-1 strains possessed *rtxA* that differed from El Tor reference N16961 by a single SNP at position 13602 of 1563748 bp (NCBI accession no. NC002505.1), corresponding to *rtxA* allele 4 (23). However, in this study, it was observed that early BD-1 strains had the *ctxB1* genotype, and over time gained the *ctxB7* genotype.

A prior study showed that Kolkata strains had four heptad repeats (TTTTGAT), whereas Haitian strains had five heptad repeats (24). All BD-0 strains had four heptad repeats (Table S1), while most BD-1 strains (93.4%; *n* = 71) had four repeats, and only 5.3% (*n* = 4) strains had five repeats. As a result, most BD-1 strains with *ctxB*7 genotypes differed from Haitian strains in ToxR binding repeats. BD-2 strains had more diversity in ToxR binding repeats with 59.0% (*n* = 62) carrying heptad repeats, 24.8% (*n* = 26) five repeats, and 16.2% (*n* = 17) three repeats.

## DISCUSSION

Vibrio cholerae biotype El Tor, the causative agent of the 7th cholera pandemic has increased transmissibility and virulence with the acquisition of classical biotype traits (14, 15). The 7th pandemic strains of cholera circulating in Asia comprises two El Tor clades, one dominant in Bangladesh and the other in India (16). Genomic analyses that included additional strains and publicly available genome sequences of wave-2 and wave-3 strains (6, 12) provide a detailed view of longitudinally and temporally representative V. cholerae clades associated with endemic cholera in Bangladesh over 27 years (1991 to 2017). The results provide new insights potentially interpretable as origin and progression, based on differences in SNPs, indels, and gene acquisition, including antibiotic resistance cassettes in BD-1 and BD-2, the latter having gained ascendency and dominance as the agent of Bangladesh endemic cholera.

Results of whole-genome sequencing (16), combined with additional genome sequence data for V. cholerae El Tor isolates of Bangladesh endemic cholera, allowed identification of two lineages, designated BD-1 and BD-2. The two clades appear to have originated from a common ancestor of paraphyletic group BD-0, as early as 1981 (95% HPD: 1976 to 1986). According to Mutreja et al. (12), seven strains of BD-0 isolated between 1991 and 2000 represent wave-2 strains, and only one strain isolated in 1994, wave-3 with a most recent common ancestor (MRCA) for BD-1 and BD-2. The BD-1 and BD-2 clades may belong to wave-3. Although BD-0 consisted predominantly of wave-2 strains, three sequenced strains isolated in 2012 shared a wave-2-like genetic background (6), suggesting wave-2 strains may have already been present. Almost all wave-3 strains from a previous study (12) were grouped with strains belonging to BD-1. Consistent with the results of a previous study (16), significant differences were noted between BD-1 and BD-2, which varied in temporal predominance as the causal agent of Bangladesh endemic cholera. Most (*n* = 62; 82%) BD-1 strains had been isolated between 2007 and 2012, with predominance during that time. Between 2005 and 2017, 105 strains belonging to BD-2 were reported, with 97 obtained between 2009 and 2017, implying BD-2 association with recent Bangladesh endemic cholera until 2017. Phylodynamic analysis using BEAST (19) revealed strains of BD-1 had been isolated in Bangladesh roughly 10 years before BD-2 strains (Fig. S1), and previously identified as Asian lineage-2 and Asian lineage-1, respectively (16).

BD-1 and BD-2 strains appear to have advanced by accumulating different SNPs and indels. Fisher exact test (21) identified 140 SNPs and 31 indel differences between BD-1 and BD-2, resulting in gene alleles unique to them (Fig. 3). Most of the SNPs and indels were components of protein-coding genes, suggesting a possible crucial role in their adaption in Bangladesh. Regression analysis of the number of SNPs and year of isolation suggested that both clades consistently accumulated SNPs over time, implying evolution in response to environmental selective pressure.

Pangenome analysis using Roary (22) provided evidence of gene acquisition by strains of the clades. A recent study of V. cholerae O1 strains isolated in Pakistan found evidence of gene acquisition, where the number of core and accessory genes varied among different lineages (25). According to the results of the analysis reported here, the number of core and accessory genes varied significantly among strains of BD-0, BD-1, and BD-2 in Bangladesh (Fig. 4A). The Pan-GWAS approach helped to identify genes unique for each clade which could be considered contributing to virulence and/or niche adaptation (26).

Phage inducible chromosomal island-like elements (PLE) protect V. cholerae populations from ICP1 infection by acting as an abortive infection system (27). In this study, the observed predominance in BD-2 of PLE1, not found in BD-0 and BD-1, could have provided a selective advantage for the lineage over BD-1, establishing dominance as an etiological agent of endemic cholera in Bangladesh in recent years.

Two BD-0 strains carried CTX phage with *ctxB*3, while other strains carried CTX phage with typical *ctxB*1. Strains at the base of BD-1 had CTX with *ctxB*1 isolated before 2007 and comprised multiple clusters. Moreover, the CTX phage of all BD-2 strains contained classical *ctxB*1. A mutation in *rtxA* creating a premature stop codon disabled toxin function in emerging V. cholerae El Tor strains bearing *ctxB*1 (24). As in the classical strains, altered El Tor pandemic strains eliminated *rtxA* after acquiring classical *ctxB*. In this study, BD-0 and BD-2 strains contained the wild-type *rtxA* allele 1 (Fig. 3A) described by Dolores and Satchell (23). None contained deletions in the *rstB* gene when reads were compared to V. cholerae N16961 reference genome, indicating *rstB* of Bangladeshi V. cholerae O1 El Tor isolates does not resemble that of the Haitian outbreak isolates that have been analyzed.

ToxR is a global transcriptional regulator of virulence gene expression, and this repeated sequence is required for ToxR binding and activation of the *ctxAB* promoter. The ToxR-binding site is located immediately upstream of *ctxAB* and the affinity of ToxR binding is influenced by the repeat sequences (28). The presence of an increased number of ToxR binding repeats located between *zot* and *ctxA* has been hypothesized to correlate with a severe form of cholera (28). In this study, variation was detected in the number of ToxR binding repeats (TTTTGAT) among sequences of the V. cholerae El Tor isolates. All BD-0 strains had four heptad repeats observed in 93.4% of BD-1 and 59% of BD-2 strains. For BD-2 strains, however, greater variation was observed in ToxR binding repeats as ca. 24.8% (*n* = 26) of BD-2 strains contained five heptad repeats, whereas 16.2% (*n* = 17) had three heptad repeats, suggesting the robustness of the clade.

Targets of quinolones are type II topoisomerases of DNA gyrase, a heterotetramer composed of two A and two B subunits, encoded by *gyrA* and *gyrB* genes, respectively (29). It was observed that all BD-1 and BD-2 strains had a common mutation Ser83 to Ile in *gyrA*, while 94.29% (99/105) BD-2 had an additional mutation Asp660 to Glu. Furthermore, 87% (66/76) of BD-1 strains exhibited a mutation Ser85 to Leu *parC*, whereas all BD-2 strains (105/105) had this mutation. In Haitian V. cholerae strains, *gyrA* and *parC* genes had two point mutations: Ser83 to Ile in *gyrA* and Ser85 to Leu in *parC*. Both are linked to quinolone resistance in V. cholerae strains associated with recent cholera outbreaks in India, Nigeria, and Cameroon (30).

SXT/R391 family ICEs are transferable elements associated with antimicrobial resistance in V. cholerae (31). The SXT-ICE regions of the isolates included in this study were compared with five sequences of the elements to the type SXT/R391 family ICEs belonging to strains associated with cholera (V. cholerae O1 and O139) (9, 32). Four BD-0 strains exhibited ICE elements similar to ICE^GEN^, ICEVchInd5, or ICEVchBan5, whereas the rest had ICE elements similar to ICE^TET^. Interestingly, ICE elements of BD-1 strains included ICE^GEN^, ICEVchInd5, or ICEVchBan5-like ICE elements, whereas BD-2 strains differed completely from the others, with only ICE^TET^-like ICE elements.

The results of the study reported here included BD-1 and BD-2 isolated during the Bangladesh endemic cholera of 2004 onwards and that, while existing together, with each subsequent year they exhibited different dominance. BD-2 diverged while retaining the ability to produce multifunctional-autoprocessing repeats-in-toxin (MARTX) and acquiring SXT element ICE^TET^ containing tetracycline resistance genes. This observation hints at a selective advantage of BD-2 strains over BD-1 strains for robustness. It is evident from the results of the analyses that BD-1 and BD-2 differ significantly, owing to gene composition and SNPs, and may have evolved independently due to selection pressures. The use of antibiotics, including tetracycline, can exert selection pressure in evolution (16, 33), while strains stopping to produce MARTX along with other variations in the genome might provide a selective advantage. According to suggestions from studies of the dynamics of V. cholerae, immunocompetence of the host against V. cholerae strains may contribute to the dynamics of V. cholerae, hence producing an effect from interaction with humans in the selection and cannot be ruled out (34).

Cholera globally is influenced by thriving populations of V. cholerae occurring naturally in the Ganges Delta of the Bay of Bengal (GDBB) (1, 2, 5, 14). Overall results presented here suggest means of emergence and progression of the two clades in evolution from a progenitor V. cholerae El Tor initiating the seventh pandemic in Asia (5) and reflecting short-term evolution of V. cholerae El Tor associated with Bangladesh endemic cholera in the GDBB (14, 31). BD-2 is concluded to have emerged relatively recently and evolved by acquiring SNPs over time. Also, BD-2 strains showed diversity in indels, possessing SXT/R391 family ICE-elements, PLE1, *tetR*, and several other important genetic elements, and predominantly associated with recent Bangladesh endemic cholera. As is apparent from our results, BD-1 appears to be an analog of a previously reported lineage 2 from Asia, the major causative agent of cholera in India, Yemen, and Haiti (16). In contrast, BD-2 strains of the present study appear to be an analog of Asian lineage 1, which successfully outcompeted BD-1 (Asian lineage 2) and established predominance as an etiological agent of cholera in a historical hot spot of the disease, Bangladesh. It can be concluded that this reflects the robustness of BD-2 as an epidemic clone emerging locally with the potential to transmit globally and underscoring the need to track the two successful V. cholerae El Tor clades.

## MATERIALS AND METHODS

### Bacterial isolates.

A total of 119 V. cholerae O1 strains from the icddr,b collection of strains isolated in Bangladesh between 2004 and 2017 (Table S1) were sequenced. Paired-end Illumina short reads for the isolated strains were generated (150 bp, 150 bp) using MiSeq or Hiseq 2500 sequencer as described in our recent study (16). Publicly available paired-end raw reads of 17 strains isolated in Bangladesh between 1991 and 2007 (see the study flow chart in Fig. S3) and 56 strains from our recent study (16) were included in the analysis.

### Genome assembly, CTX-prophage typing, and gene annotation.

An ultrafast FASTQ preprocessor implemented in FASTP (35), was used to inspect raw paired-end reads and filter bad ligation or adapter parts. *De novo* genome assembly implemented in VelvetOptimizer (36) was used to build contigs by optimizing the parameter *N*_50_, a metric for assessing the continuity of an assembly. PHASTER, a rapid prophage sequence identification and annotation web server (37), was used to extract CTX-prophage, which was subsequently typed according to mutations in *rstA*, *rstB*, and *ctxB* (7). The bacterial genome annotation tool, Prokka (38), was used for whole-genome gene annotation. ResFinder (39) was used to find the antimicrobial-resistant gene profiles for all the strains.

### SNP identification and phylogenetic analysis.

Bowtie2 (40) was used to align high-quality reads with the reference genome sequence of V. cholerae N16961 El Tor (NCBI accession no. NC_002505.1 and NC_002506.1) for variant calling. Samtools (41) and Bcftools (42) were used to call genome variants. A maximum-likelihood phylogeny was inferred on an alignment of concatenated SNPs evenly distributed across a nonrepetitive, nonrecombinant core genome using IQ-TREE v1.6.1 (43). Trees were visualized in FigTree v1.4.3 (http://tree.bio.ed.ac.uk/software/figtree/) or Interactive Tree of Life online tool (44).

### Bayesian phylogenetic inference.

The Bayesian Evolutionary Analysis Sampling Trees (BEAST) v.2.4.4 software package (19) was used for temporal analysis to estimate the divergence date of V. cholerae O1 isolates in Bangladesh. The date of isolation of each strain was used as tip data. A random clock model was implemented using Markov Chain Monte Carlo (MCMC) chains run for 100 million generations with 10% burn-in and sampled every 1000 generations. A GTR nucleotide substitution model was used. Tree data were summarized using TreeAnotator, a tool of BEAST software package, to generate the maximum clade credibility tree.

### Pangenome analysis.

A pangenome was constructed using Roary (22) from annotated assemblies of the sample set with a percentage protein identity of 95%. The protein sequences were first extracted and iteratively preclustered with cd-hit (version 4.6) down to 98% identity. An all against all blast (version 2.2.31) was performed on the remaining nonclustered sequences and a single representative sequence from each cd-hit cluster was selected. The data were used by MCL (45) (version 11-294) to cluster the sequences. The preclusters and MCL clusters were merged, and paralogs split by inspecting the conserved gene neighborhood around each sequence (5 genes on either side). Each sequence for each cluster was independently aligned using PRANK (46) (version 0.140603) and combined to form a multi-FASTA alignment of the core genes. Sequences of SXT elements were compared with ICE^GEN^ and ICE^TET^ using BRIG 0.95 with 70% BLAST identity (47).

### Data availability.

Nucleotide sequence data generated in this study are available in the DDBJ/EMBL/GenBank databases under BioProject ID PRJDB12727.
