# Supplementary material for: Genomic Characteristics of Recently Recognized Vibrio cholerae El Tor Lineages Associated with Cholera in Bangladesh, 1991 to 2017
Source: Microbiol Spectr. 2022 Mar 22;10(2):e00391-22. doi: 10.1128/spectrum.00391-22 (PMC9045249; doi:10.1128/spectrum.00391-22)
Supplement: SUPPLEMENTAL FILE 1 — Supplemental material. Download SPECTRUM00391-22_Supp_1_seq1.pdf, PDF file, 2.2 MB [file spectrum00391-22_supp_1_seq1.pdf]

Genomic characteristics of recently recognized *Vibrio cholerae* El Tor lineages associated with cholera in Bangladesh, 1991-2017

Authors:

Md Mamun Monir[1], Talal Hossain[1], Masatomo Morita[2], Makoto Ohnishi[2], Fatema-Tuz Johura[1], Marzia Sultana[1], Shirajum Monira[1], Tahmeed Ahmed[1], Nicholas Thomson[3], Haruo Watanabe[2], Anwar Huq[4], Rita R. Colwell[4,5], Kimberley Seed[6], and Munirul Alam[1§].

Table S1. Genetic characteristics of strains included in the study

Lineage	Strain ID	Year	Source	Reference	Accession	SXT ICE	Acquired antibiotic resistance profile	gyrA	Tox R	ctxB	rstA	CTX	PLE
BD-0	4670	1991	No data	Mutreja et al. 2011, Nature	ERR019883	ICEVflInd1	ant(3")-Ia, catB9, sul1, qacE	El tor gyrA	4	ctxB_1	TTAC	CTX-2	PLE(-)
	MG116025	1991	No data	Mutreja et al. 2011, Nature	ERR018122	ICEgen	catB9, dfrA1	El tor gyrA	4	ctxB_3	CTT	CTX-3	PLE(-)
	MG116226	1991	No data	Mutreja et al. 2011, Nature	ERR025396	ICEVchBan5	aph(3")-Ib, aph(6)-Id, catB9, dfrA1, floR, sul2	El tor gyrA	4	ctxB_3	CTT	CTX-3	PLE(-)
	4660	1994	No data	Mutreja et al. 2011, Nature	ERR018117	ICEgen	aph(3")-Ib, aph(6)-Id, catB9, sul2	El tor gyrA	4	ctxB_1	CTT	CTX-3	PLE(-)
	A346_1	1994	No data	Mutreja et al. 2011, Nature	ERR025392	ICEtet	aph(3")-Ib, aph(6)-Id, catB9, dfrA1, floR, sul2, tet(A)	Ser83 to ARG	4	ctxB_1	TTAC	CTX-2	PLE(-)
	A346_2	1994	No data	Mutreja et al. 2011, Nature	ERR018179	ICEVchInd5	aph(6)-Id, catB9, dfrA1, sul2	Ser83 to ARG	4	ctxB_1	TTAC	CTX-2	PLE(-)
	MJ1485	1994	No data	Mutreja et al. 2011, Nature	ERR018120	ICEVchInd4	aph(3")-Ib, aph(6)-Id, catB9, dfrA1, floR, sul2	El tor gyrA	4	ctxB_1	TTAC	CTX-2	PLE(-)
	4672	2000	No data	Mutreja et al. 2011, Nature	ERR019884	ICEtet	aph(3")-Ib, aph(6)-Id, catB9, floR, tet(A)	El tor gyrA	4	ctxB_1	TTAC	CTX-2	PLE(-)
	MAB035	2012	Env	This study	DRR335720	ICEtet	aph(3")-Ib, aph(6)-Id, catB9, dfrA1, floR, sul2, tet(A)	El tor gyrA	4	ctxB_1	TTAC	CTX-2	PLE(-)
	MAB037	2012	Env	This study	DRR335721	ICEtet	aph(3")-Ib, aph(6)-Id, catB9, dfrA1, floR, sul2, tet(A)	El tor gyrA	4	ctxB_1	TTAC	CTX-2	PLE(-)
	MAB039	2012	Clinical	This study	DRR335723	ICEtet	aph(3")-Ib, aph(6)-Id, catB9, dfrA1, floR, sul2, tet(A)	Asn253 to Asp	4	ctxB_1	TTAC	CTX-2	PLE(-)
BD-1	4679	1999	No data	Mutreja et al. 2011, Nature	ERR018114	ICEgen	aph(3")-Ib, aph(6)-Id, catB9, dfrA1, floR, sul2	Haitian gyrA Ser83 to Ile	4	ctxB_1	CTT	CTX-3	PLE(-)
	4661	2001	No data	Mutreja et al. 2011, Nature	ERR018116	ICEgen	aph(3")-Ib, aph(6)-Id, catB9, dfrA1, floR, sul2	Haitian gyrA Ser83 to Ile	4	ctxB_1	CTT	CTX-3	PLE(-)
	4662	2001	No data	Mutreja et al. 2011, Nature	ERR025373	ICEgen	aph(3")-Ib, aph(6)-Id, catB9, dfrA1, floR, sul2	Haitian gyrA Ser83 to Ile	4	ctxB_1	CTT	CTX-3	PLE(-)
	4663	2001	No data	Mutreja et al. 2011, Nature	ERR018115	ICEgen	aph(3")-Ib, aph(6)-Id, catB9, dfrA1, floR, sul2	Haitian gyrA Ser83 to Ile	4	ctxB_1	CTT	CTX-3	PLE(-)

	4675	2001	No data	Mutreja et al. 2011, Nature	ERR018113	ICEgen	aph(6)-Id, catB9, dfrA1, sul2	Haitian gyrA Ser83 to Ile	4	ctxB_1	CTT	CTX-3	PLE(-)
	MAB001	2004	Env	This study	DRR335691	ICEVchInd5	aph(3")-Ib, aph(6)-Id, catB9, dfrA1, floR, sul2	Haitian gyrA Ser83 to Ile	4	ctxB_1	CTT	CTX-3	PLE(-)
	MAB002	2004	Clinical	This study	DRR335692	ICEVchInd5	aph(3")-Ib, aph(6)-Id, catB9, dfrA1, floR, sul2	Haitian gyrA Ser83 to Ile	4	ctxB_7	CTT	CTX-3b	PLE(-)
	MAB005	2005	Env	This study	DRR335695	ICEVchInd5	aph(3")-Ib, aph(6)-Id, catB9, dfrA1, floR, sul2	Haitian gyrA Ser83 to Ile	4	ctxB_1	CTT	CTX-3	PLE2
	MAB007	2005	Clinical	This study	DRR335697	ICEVchInd5	aph(3")-Ib, aph(6)-Id, catB9, dfrA1, floR, sul2	Haitian gyrA Ser83 to Ile	4	ctxB_1	CTT	CTX-3	PLE(-)
	A488_1	2006	No data	Mutreja et al. 2011, Nature	ERR025393	ICEVchBan5	aph(3")-Ib, aph(6)-Id, catB9, dfrA1, floR, sul2	Haitian gyrA Ser83 to Ile	4	ctxB_1	CTT	CTX-3	PLE(-)
	A488_2	2006	No data	Mutreja et al. 2011, Nature	ERR018128	ICEgen	aph(3")-Ib, aph(6)-Id, catB9, dfrA1, floR, sul2	Haitian gyrA Ser83 to Ile	4	ctxB_1	CTT	CTX-3	PLE(-)
	A487_1	2007	No data	Mutreja et al. 2011, Nature	ERR025395	ICEVchBan5	aph(3")-Ib, aph(6)-Id, catB9, dfrA1, floR, sul2	Haitian gyrA Ser83 to Ile	4	ctxB_1	CTT	CTX-3	PLE(-)
	A487_2	2007	No data	Mutreja et al. 2011, Nature	ERR018182	ICEgen	aph(3")-Ib, aph(6)-Id, catB9, dfrA1	Haitian gyrA Ser83 to Ile	4	ctxB_1	CTT	CTX-3	PLE(-)
	BGD004	2007	Clinical	This study	DRR335607	ICEVchInd5	aph(3")-Ib, aph(6)-Id, catB9, dfrA1, floR, sul2	Haitian gyrA Ser83 to Ile	4	ctxB_1	CTT	CTX-3	PLE2
	BGD006	2007	Clinical	This study	DRR335609	ICEVchInd5	aph(3")-Ib, aph(6)-Id, catB9, dfrA1, floR, sul2	Haitian gyrA Ser83 to Ile	4	ctxB_1	CTT	CTX-3	PLE2
	BGD007	2007	Clinical	This study	DRR335610	ICEVchInd5	aph(3")-Ib, aph(6)-Id, catB9, dfrA1, floR, sul2	Haitian gyrA Ser83 to Ile	4	ctxB_7	CTT	CTX-3b	PLE(-)
	BGD008	2007	Clinical	This study	DRR335611	ICEVchInd5	aph(3")-Ib, aph(6)-Id, catB9, dfrA1, floR, sul2	Haitian gyrA Ser83 to Ile	4	ctxB_7	CTT	CTX-3b	PLE(-)
	BGD009	2007	Clinical	This study	DRR335612	ICEVchInd5	catB9, dfrA1	Haitian gyrA Ser83 to Ile	5	ctxB_1	CTT	CTX-3	PLE2
	BGD010	2007	Clinical	This study	DRR335613	ICEVchInd5	aph(3")-Ib, aph(6)-Id, catB9, dfrA1, floR, sul2	Haitian gyrA Ser83 to Ile	untypable	ctxB_1	CTT	CTX-3	PLE2
	BGD011	2007	Clinical	This study	DRR335614	ICEVchInd5	aph(3")-Ib, aph(6)-Id, catB9, dfrA1, floR, sul2	Haitian gyrA Ser83 to Ile	4	ctxB_7	CTT	CTX-3b	PLE(-)
	BGD012	2007	Clinical	This study	DRR335615	ICEVchInd5	aph(3")-Ib, aph(6)-Id, catB9, dfrA1, floR, sul2	Haitian gyrA Ser83 to Ile	4	ctxB_1	CTT	CTX-3	PLE2
	BGD013	2007	Clinical	This study	DRR335616	ICEVchBan5	aph(3")-Ib, aph(6)-Id, catB9, dfrA1, floR, sul2	Haitian gyrA Ser83 to Ile	5	ctxB_1	CTT	CTX-3	PLE2
	BGD014	2007	Clinical	This study	DRR335617	ICEVchInd5	catB9, dfrA1	Haitian gyrA Ser83 to Ile	5	ctxB_1	CTT	CTX-3	PLE2

	BGD015	2007	Clinical	This study	DRR335618	ICEVchInd5	aph(3")-Ib, aph(6)-Id, catB9, dfrA1, floR, sul2	Haitian gyrA Ser83 to Ile	4	ctxB_7	CTT	CTX-3b	PLE(-)
	MAB011	2007	Env	This study	DRR335700	ICEVchInd5	aph(3")-Ib, aph(6)-Id, catB9, dfrA1, floR, sul2	Haitian gyrA Ser83 to Ile	4	ctxB_1	CTT	CTX-3	PLE2
	MAB012	2007	Clinical	This study	DRR335701	ICEVchInd5	aph(3")-Ib, aph(6)-Id, catB9, dfrA1, floR, sul2	Haitian gyrA Ser83 to Ile	4	ctxB_1	CTT	CTX-3	PLE(-)
	MAB013	2007	Clinical	This study	DRR335702	ICEVchInd5	aph(3")-Ib, aph(6)-Id, catB9, dfrA1, floR, sul2	Haitian gyrA Ser83 to Ile	4	ctxB_1	CTT	CTX-3	PLE2
	BGD016	2008	Clinical	This study	DRR335619	ICEVchInd5	aph(3")-Ib, aph(6)-Id, catB9, dfrA1, floR, sul2	Haitian gyrA Ser83 to Ile	4	ctxB_7	CTT	CTX-3b	PLE(-)
	BGD018	2008	Clinical	This study	DRR335620	ICEVchInd5	aph(3")-Ib, aph(6)-Id, catB9, dfrA1, floR, sul2	Haitian gyrA Ser83 to Ile	4	ctxB_7	CTT	CTX-3b	PLE(-)
	BGD019	2008	Clinical	This study	DRR335621	ICEVchInd5	aph(3")-Ib, aph(6)-Id, catB9, dfrA1, floR, sul2	Haitian gyrA Ser83 to Ile	4	ctxB_7	CTT	CTX-3b	PLE(-)
	BGD020	2008	Clinical	This study	DRR335622	ICEVchInd5	aph(3")-Ib, aph(6)-Id, catB9, dfrA1, floR, sul2	Haitian gyrA Ser83 to Ile	4	ctxB_7	CTT	CTX-3b	PLE(-)
	BGD021	2008	Clinical	This study	DRR335623	ICEVchInd5	aph(3")-Ib, aph(6)-Id, catB9, dfrA1, floR, sul2	Haitian gyrA Ser83 to Ile	4	ctxB_7	CTT	CTX-3b	PLE(-)
	BGD022	2008	Clinical	This study	DRR335624	ICEVchInd5	aph(3")-Ib, aph(6)-Id, catB9, dfrA1, floR, sul2	Haitian gyrA Ser83 to Ile	4	ctxB_7	CTT	CTX-3b	PLE(-)
	BGD023	2008	Clinical	This study	DRR335625	ICEVchInd5	aph(3")-Ib, aph(6)-Id, catB9, dfrA1, floR, sul2	Haitian gyrA Ser83 to Ile	4	ctxB_7	CTT	CTX-3b	PLE(-)
	BGD024	2008	Clinical	This study	DRR335626	ICEVchInd5	aph(3")-Ib, aph(6)-Id, catB9, dfrA1, floR, sul2	Haitian gyrA Ser83 to Ile	4	ctxB_7	CTT	CTX-3b	PLE(-)
	BGD025	2008	Clinical	This study	DRR335627	ICEVchInd5	aph(3")-Ib, aph(6)-Id, catB9, dfrA1, floR, sul2	Haitian gyrA Ser83 to Ile	4	ctxB_7	CTT	CTX-3b	PLE(-)
	BGD107	2009	No data	Morita et al. 2020, mBio	DRX179743	ICEVchInd5	aph(3")-Ib, aph(6)-Id, catB9, dfrA1, floR, sul2	Haitian gyrA Ser83 to Ile	4	ctxB_7	CTT	CTX-3b	PLE(-)
	BGD037	2010	Clinical	This study	DRR335635	ICEVchInd5	aph(3")-Ib, aph(6)-Id, catB9, dfrA1, floR, sul2	Haitian gyrA Ser83 to Ile	4	ctxB_7	CTT	CTX-3b	PLE(-)
	BGD038	2010	Clinical	This study	DRR335636	ICEVchInd5	aph(3")-Ib, aph(6)-Id, catB9, dfrA1, floR, sul2	Haitian gyrA Ser83 to Ile	4	ctxB_7	CTT	CTX-3b	PLE(-)
	BGD039	2010	Clinical	This study	DRR335637	ICEVchInd5	aph(3")-Ib, aph(6)-Id, catB9, dfrA1, floR, sul2	Haitian gyrA Ser83 to Ile	4	ctxB_7	CTT	CTX-3b	PLE(-)
	BGD040	2010	Clinical	This study	DRR335638	ICEVchInd5	aph(3")-Ib, aph(6)-Id, catB9, dfrA1, floR, sul2	Haitian gyrA Ser83 to Ile	4	ctxB_7	CTT	CTX-3b	PLE(-)
	BGD041	2010	Clinical	This study	DRR335639	ICEVchInd5	aph(3")-Ib, aph(6)-Id, catB9, dfrA1, floR, sul2	Haitian gyrA Ser83 to Ile	4	ctxB_7	CTT	CTX-3b	PLE(-)

	BGD042	2010	Clinical	This study	DRR335640	ICEVchInd5	aph(3")-Ib, aph(6)-Id, catB9, dfrA1, floR, sul2	Haitian gyrA Ser83 to Ile	4	ctxB_7	CTT	CTX-3b	PLE(-)
	BGD043	2010	Clinical	Morita et al. 2020, mBio	DRX179812	ICEVchInd5	aph(3")-Ib, aph(6)-Id, catB9, dfrA1, floR, sul2	Haitian gyrA Ser83 to Ile	4	ctxB_7	CTT	CTX-3b	PLE(-)
	BGD044	2010	Clinical	This study	DRR335641	ICEVchInd5	aph(3")-Ib, aph(6)-Id, catB9, dfrA1, floR, sul2	Haitian gyrA Ser83 to Ile	4	ctxB_7	CTT	CTX-3b	PLE(-)
	BGD045	2010	Clinical	This study	DRR335642	ICEVchInd5	aph(3")-Ib, aph(6)-Id, catB9, dfrA1, floR, sul2	Haitian gyrA Ser83 to Ile	4	ctxB_7	CTT	CTX-3b	PLE(-)
	BGD047	2010	Clinical	This study	DRR335644	ICEVchInd5	aph(3")-Ib, aph(6)-Id, catB9, dfrA1, floR, sul2	Haitian gyrA Ser83 to Ile	4	ctxB_7	CTT	CTX-3b	PLE(-)
	BGD048	2010	Clinical	This study	DRR335645	ICEVchInd5	aph(3")-Ib, aph(6)-Id, catB9, dfrA1, floR, sul2	Haitian gyrA Ser83 to Ile	4	ctxB_7	CTT	CTX-3b	PLE(-)
	BGD049	2010	Clinical	This study	DRR335646	ICEVchInd5	aph(3")-Ib, aph(6)-Id, catB9, dfrA1, floR, sul2	Haitian gyrA Ser83 to Ile	4	ctxB_7	CTT	CTX-3b	PLE(-)
	BGD110	2010	No data	Morita et al. 2020, mBio	DRX179745	ICEVchInd5	aph(3")-Ib, aph(6)-Id, catB9, dfrA1, floR, sul2	Haitian gyrA Ser83 to Ile	4	ctxB_7	CTT	CTX-3b	PLE(-)
	BGD111	2010	No data	Morita et al. 2020, mBio	DRX179746	ICEVchInd5	aph(3")-Ib, aph(6)-Id, catB9, dfrA1, floR, sul2	Haitian gyrA Ser83 to Ile	4	ctxB_7	CTT	CTX-3b	PLE(-)
	BGD112	2010	No data	This study	DRR335669	ICEVchInd5	aph(3")-Ib, aph(6)-Id, catB9, dfrA1, floR, sul2	Haitian gyrA Ser83 to Ile, Gly650 Asp	4	ctxB_7	CTT	CTX-3b	PLE(-)
	BGD113	2010	No data	Morita et al. 2020, mBio	DRX179747	ICEVchInd5	aph(3")-Ib, aph(6)-Id, catB9, dfrA1, floR, sul2	Haitian gyrA Ser83 to Ile	5	ctxB_7	CTT	CTX-3b	PLE(-)
	MAB018	2010	Env	This study	DRR335706	ICEVchInd5	aph(3")-Ib, aph(6)-Id, catB9, dfrA1, floR, sul2	Haitian gyrA Ser83 to Ile	4	ctxB_7	CTT	CTX-3b	PLE(-)
	MAB019	2010	Env	This study	DRR335707	ICEVchInd5	aph(3")-Ib, aph(6)-Id, catB9, dfrA1, floR, sul2	Haitian gyrA Ser83 to Ile	4	ctxB_7	CTT	CTX-3b	PLE(-)
	MAB021	2010	Env	This study	DRR335708	ICEVchInd5	aph(3")-Ib, aph(6)-Id, catB9, dfrA1, floR, sul2	Haitian gyrA Ser83 to Ile	4	ctxB_7	CTT	CTX-3b	PLE(-)
	BGD050	2011	Clinical	Morita et al. 2020, mBio	DRX179775	ICEVchInd5	aph(3")-Ib, aph(6)-Id, catB9, dfrA1, floR, sul2	Haitian gyrA Ser83 to Ile	4	ctxB_7	CTT	CTX-3b	PLE(-)
	BGD051	2011	Clinical	This study	DRR335647	ICEVchInd5	aph(3")-Ib, aph(6)-Id, catB9, dfrA1, floR, sul2	Haitian gyrA Ser83 to Ile	4	ctxB_7	CTT	CTX-3b	PLE(-)
	BGD052	2011	Clinical	This study	DRR335648	ICEVchInd5	aph(3")-Ib, aph(6)-Id, catB9, dfrA1, floR, sul2	Haitian gyrA Ser83 to Ile	4	ctxB_7	CTT	CTX-3b	PLE(-)
	BGD053	2011	Clinical	This study	DRR335649	ICEVchInd5	aph(3")-Ib, aph(6)-Id, catB9, dfrA1, floR, sul2	Haitian gyrA Ser83 to Ile	4	ctxB_7	CTT	CTX-3b	PLE(-)

	BGD056	2011	Clinical	Morita et al. 2020, mBio	DRX179776	ICEVchInd5	aph(3")-Ib, aph(6)-Id, catB9, dfrA1, floR, sul2	Haitian gyrA Ser83 to Ile	4	ctxB_7	CTT	CTX-3b	PLE(-)
	BGD057	2011	Clinical	Morita et al. 2020, mBio	DRX179778	ICEVchInd5	aph(3")-Ib, aph(6)-Id, catB9, dfrA1, floR, sul2	Haitian gyrA Ser83 to Ile	4	ctxB_7	CTT	CTX-3b	PLE(-)
	BGD058	2011	Clinical	Morita et al. 2020, mBio	DRX179779	ICEVchInd5	aph(3")-Ib, aph(6)-Id, catB9, dfrA1, floR, sul2	Haitian gyrA Ser83 to Ile	4	ctxB_7	CTT	CTX-3b	PLE(-)
	BGD114	2011	No data	Morita et al. 2020, mBio	DRX179748	ICEVchInd5	aph(3")-Ib, aph(6)-Id, catB9, dfrA1, floR, sul2	Haitian gyrA Ser83 to Ile	4	ctxB_7	CTT	CTX-3b	PLE(-)
	BGD115	2011	No data	This study	DRR335670	ICEVchInd5	aph(3")-Ib, aph(6)-Id, catB9, dfrA1, floR, sul2	Haitian gyrA Ser83 to Ile	4	ctxB_7	CTT	CTX-3b	PLE(-)
	BGD116	2011	No data	Morita et al. 2020, mBio	DRX179749	ICEVchInd5	aph(3")-Ib, aph(6)-Id, catB9, dfrA1, floR, sul2	Haitian gyrA Ser83 to Ile	4	ctxB_7	CTT	CTX-3b	PLE(-)
	BGD060	2012	Clinical	Morita et al. 2020, mBio	DRX179773	ICEVchInd5	aph(3")-Ib, aph(6)-Id, catB9, dfrA1, floR, sul2	Haitian gyrA Ser83 to Ile	4	ctxB_7	CTT	CTX-3b	PLE(-)
	BGD061	2012	Clinical	This study	DRR335650	ICEVchInd5	aph(3")-Ib, aph(6)-Id, catB9, dfrA1, floR, sul2	Haitian gyrA Ser83 to Ile	4	ctxB_7	CTT	CTX-3b	PLE(-)
	BGD064	2012	Clinical	This study	DRR335651	ICEVchInd5	aph(3")-Ib, aph(6)-Id, catB9, dfrA1, floR, sul2	Haitian gyrA Ser83 to Ile	4	ctxB_7	CTT	CTX-3b	PLE(-)
	BGD065	2012	Clinical	Morita et al. 2020, mBio	DRX179774	ICEVchInd5	aph(3")-Ib, aph(6)-Id, catB9, dfrA1, floR, sul2	Haitian gyrA Ser83 to Ile	4	ctxB_7	CTT	CTX-3b	PLE(-)
	BGD066	2012	Clinical	This study	DRR335652	ICEVchInd5	aph(3")-Ib, aph(6)-Id, catB9, dfrA1, floR, sul2	Haitian gyrA Ser83 to Ile	4	ctxB_7	CTT	CTX-3b	PLE(-)
	BGD067	2012	Clinical	Morita et al. 2020, mBio	DRX179777	ICEVchInd5	aph(3")-Ib, aph(6)-Id, catB9, dfrA1, floR, sul2	Haitian gyrA Ser83 to Ile	4	ctxB_7	CTT	CTX-3b	PLE(-)
	BGD068	2012	Clinical	This study	DRR335653	ICEVchInd5	aph(3")-Ib, aph(6)-Id, catB9, dfrA1, floR, sul2	Haitian gyrA Ser83 to Ile	4	ctxB_7	CTT	CTX-3b	PLE(-)
	BGD125	2014	No data	This study	DRR335672	ICEVchInd5	aph(3")-Ib, aph(6)-Id, catB9, dfrA1, floR, sul2	Haitian gyrA Ser83 to Ile	4	ctxB_7	CTT	CTX-3b	PLE(-)
	BGD126	2015	No data	Morita et al. 2020, mBio	DRX179757	ICEVchInd5	aph(3")-Ib, aph(6)-Id, catB9, dfrA1, floR, sul2	Haitian gyrA Ser83 to Ile	4	ctxB_7	CTT	CTX-3b	PLE(-)
	BGD137	2016	No data	Morita et al. 2020, mBio	DRX179763	ICEVchInd5	aph(3")-Ib, aph(6)-Id, catB9, dfrA1, floR, sul2	Haitian gyrA Ser83 to Ile	4	ctxB_7	CTT	CTX-3b	PLE(-)
**BD-2**	BGD123	2013	No data	This study	DRR335671	ICEtet	aph(3")-Ib, aph(6)-Id, catB9, dfrA1, sul2, tet(59)	Haitian gyrA Ser83 to Ile, Asp660 to Glu	5	ctxB_1	CTT	CTX-3	PLE1
	BGD135	2015	No data	This study	DRR335676	ICEtet	aph(3")-Ib, aph(6)-Id, catB9, dfrA1, sul2, tet(59)	Haitian gyrA Ser83 to Ile,	5	ctxB_1	CTT	CTX-3	PLE1

								Asp660 to Glu					
	BGD136	2015	No data	Morita et al. 2020, mBio	DRX179762	ICEtet	aph(3")-Ib, aph(6)-Id, catB9, dfrA1, sul2, tet(59)	Haitian gyrA Ser83 to Ile, ASP660 to Glu	5	ctxB_1	CTT	CTX-3	PLE1
	BGD139	2016	No data	Morita et al. 2020, mBio	DRX179765	ICEtet	aph(3")-Ib, aph(6)-Id, catB9, dfrA1, sul2, tet(59)	Haitian gyrA Ser83 to Ile, ASP660 to Glu	5	ctxB_1	CTT	CTX-3	PLE1
	BGD140	2016	No data	Morita et al. 2020, mBio	DRX179766	ICEtet	aph(3")-Ib, aph(6)-Id, catB9, dfrA1, sul2, tet(59)	Haitian gyrA Ser83 to Ile, ASP660 to Glu	4	ctxB_1	CTT	CTX-3	PLE1
	BGD141	2016	No data	Morita et al. 2020, mBio	DRX179767	ICEtet	aph(3")-Ib, aph(6)-Id, catB9, dfrA1, sul2, tet(59)	Haitian gyrA Ser83 to Ile, ASP660 to Glu	5	ctxB_1	CTT	CTX-3	PLE1
	BGD143	2016	No data	Morita et al. 2020, mBio	DRX179768	ICEtet	aph(3")-Ib, aph(6)-Id, catB9, dfrA1, sul2, tet(59)	Haitian gyrA Ser83 to Ile, ASP660 to Glu	3	ctxB_1	-	CTX-3	PLE1
	BGD144	2016	No data	This study	DRR335677	ICEtet	aph(3")-Ib, aph(6)-Id, catB9, dfrA1, sul2, tet(59)	Haitian gyrA Ser83 to Ile, ASP660 to Glu	3	ctxB_1	CTT	CTX-3	PLE1
	BGD145	2016	No data	This study	DRR335678	ICEtet	aph(3")-Ib, aph(6)-Id, catB9, dfrA1, sul2, tet(59)	Haitian gyrA Ser83 to Ile, ASP660 to Glu	3	ctxB_1	CTT	CTX-3	PLE1
	BGD146	2016	No data	Morita et al. 2020, mBio	DRX179769	ICEtet	aph(3")-Ib, aph(6)-Id, catB9, dfrA1, sul2, tet(59)	Haitian gyrA Ser83 to Ile, ASP660 to Glu	3	ctxB_1	CTT	CTX-3	PLE1
	BGD147	2016	No data	This study	DRR335679	ICEtet	aph(3")-Ib, aph(6)-Id, catB9, dfrA1, sul2, tet(59)	Haitian gyrA Ser83 to Ile, ASP660 to Glu	3	ctxB_1	CTT	CTX-3	PLE1
	BGD148	2016	No data	Morita et al. 2020, mBio	DRX179770	ICEtet	aph(3")-Ib, aph(6)-Id, catB9, dfrA1, sul2, tet(59)	Haitian gyrA Ser83 to Ile, ASP660 to Glu	5	ctxB_1	CTT	CTX-3	PLE1
	BGD149	2016	No data	Morita et al. 2020, mBio	DRX179771	ICEtet	aph(3")-Ib, aph(6)-Id, catB9, dfrA1, sul2, tet(59)	Haitian gyrA Ser83 to Ile, ASP660 to Glu	3	ctxB_1	CTT	CTX-3	PLE1

	BGD150	2017	No data	This study	DRR335680	Untypeable	catB9	Haitian gyrA Ser83 to Ile, ASP660 to Glu	4	ctxB_1	CTT	CTX-3	PLE1
	BGD151	2017	No data	This study	DRR335681	Untypeable	catB9	Haitian gyrA Ser83 to Ile, ASP660 to Glu	4	ctxB_1	CTT	CTX-3	PLE1
	BGD152	2017	No data	This study	DRR335682	ICEtet	aph(3")-Ib, aph(6)-Id, catB9, dfrA1, sul2, tet(59)	Haitian gyrA Ser83 to Ile, ASP660 to Glu	3	ctxB_1	CTT	CTX-3	PLE(-)
	BGD153	2017	No data	This study	DRR335683	ICEtet	aph(3")-Ib, aph(6)-Id, catB9, dfrA1, sul2, tet(59)	Haitian gyrA Ser83 to Ile, ASP660 to Glu	4	ctxB_1	CTT	CTX-3	PLE1
	BGD154	2017	No data	This study	DRR335684	ICEtet	aph(3")-Ib, aph(6)-Id, catB9, dfrA1, sul2, tet(59)	Haitian gyrA Ser83 to Ile, ASP660 to Glu	3	ctxB_1	CTT	CTX-3	PLE(-)
	BGD155	2017	No data	This study	DRR335685	ICEtet	aph(3")-Ib, aph(6)-Id, catB9, dfrA1, sul2, tet(59)	Haitian gyrA Ser83 to Ile, ASP660 to Glu	4	ctxB_1	CTT	CTX-3	PLE1
	BGD156	2017	No data	This study	DRR335686	ICEtet	aph(3")-Ib, aph(6)-Id, catB9, dfrA1, sul2, tet(59)	Haitian gyrA Ser83 to Ile, ASP660 to Glu	5	ctxB_1	CTT	CTX-3	PLE1
	BGD157	2017	No data	This study	DRR335687	ICEtet	aph(3")-Ib, aph(6)-Id, catB9, dfrA1, sul2, tet(59)	Haitian gyrA Ser83 to Ile, ASP660 to Glu	3	ctxB_1	CTT	CTX-3	PLE(-)
	BGD158	2017	No data	This study	DRR335688	ICEtet	aph(3")-Ib, aph(6)-Id, catB9, dfrA1, sul2, tet(59)	Haitian gyrA Ser83 to Ile, ASP660 to Glu	4	ctxB_1	CTT	CTX-3	PLE1
	BGD159	2017	No data	This study	DRR335689	ICEtet	aph(3")-Ib, aph(6)-Id, catB9, dfrA1, sul2, tet(59)	Haitian gyrA Ser83 to Ile, ASP660 to Glu	5	ctxB_1	CTT	CTX-3	PLE1
	BGD160	2017	No data	This study	DRR335690	Untypeable	catB9	Haitian gyrA Ser83 to Ile, ASP660 to Glu	3	ctxB_1	CTT	CTX-3	PLE(-)

	BGD036	2010	Clinical	Morita et al. 2020, mBio	DRX179811	ICEtet	aph(3")-Ib, aph(6)-Id, catB9, dfrA1, sul2, tet(59)	Haitian gyrA Ser83 to Ile, ASP660 to Glu	4	ctxB_1	CTT	CTX-3	PLE1
	BGD070	2013	Clinical	Morita et al. 2020, mBio	DRX179780	ICEtet	aph(3")-Ib, aph(6)-Id, catB9, dfrA1, sul2, tet(59)	Haitian gyrA Ser83 to Ile, ASP660 to Glu	4	ctxB_1	CTT	CTX-3	PLE1
	BGD071	2013	Clinical	This study	DRR335654	ICEtet	aph(3")-Ib, aph(6)-Id, catB9, dfrA1, sul2, tet(59)	Haitian gyrA Ser83 to Ile, ASP660 to Glu	4	ctxB_1	CTT	CTX-3	PLE1
	BGD072	2013	Clinical	Morita et al. 2020, mBio	DRX179781	ICEtet	aph(3")-Ib, aph(6)-Id, catB9, dfrA1, sul2, tet(59)	Haitian gyrA Ser83 to Ile, ASP660 to Glu	4	ctxB_1	CTT	CTX-3	PLE(-)
	BGD073	2013	Clinical	This study	DRR335655	ICEtet	aph(3")-Ib, aph(6)-Id, catB9, dfrA1, sul2, tet(59)	Haitian gyrA Ser83 to Ile, ASP660 to Glu	4	ctxB_1	CTT	CTX-3	PLE1
	BGD074	2013	Clinical	This study	DRR335656	ICEtet	aph(3")-Ib, aph(6)-Id, catB9, dfrA1, sul2, tet(59)	Haitian gyrA Ser83 to Ile, ASP660 to Glu	5	ctxB_1	CTT	CTX-3	PLE1
	BGD075	2013	Clinical	This study	DRR335657	ICEtet	aph(3")-Ib, aph(6)-Id, catB9, dfrA1, sul2, tet(59)	Haitian gyrA Ser83 to Ile, ASP660 to Glu	3	ctxB_1	CTT	CTX-3	PLE1
	BGD077	2013	Clinical	Morita et al. 2020, mBio	DRX179783	ICEtet	aph(3")-Ib, aph(6)-Id, catB9, dfrA1, sul2, tet(59)	Haitian gyrA Ser83 to Ile, ASP660 to Glu	5	ctxB_1	CTT	CTX-3	PLE1
	BGD078	2013	Clinical	This study	DRR335658	ICEtet	aph(3")-Ib, aph(6)-Id, catB9, dfrA1, sul2, tet(59)	Haitian gyrA Ser83 to Ile, ASP660 to Glu	5	ctxB_1	CTT	CTX-3	PLE1
	BGD079	2013	Clinical	Morita et al. 2020, mBio	DRX179785	ICEtet	aph(3")-Ib, aph(6)-Id, catB9, dfrA1, sul2, tet(59)	Haitian gyrA Ser83 to Ile, ASP660 to Glu	4	ctxB_1	CTT	CTX-3	PLE1
	BGD080	2013	Clinical	This study	DRR335659	ICEtet	aph(3")-Ib, aph(6)-Id, catB9, dfrA1, sul2, tet(59)	Haitian gyrA Ser83 to Ile, ASP660 to Glu	5	ctxB_1	CTT	CTX-3	PLE1

	BGD082	2013	Clinical	Morita et al. 2020, mBio	DRX179791	ICEtet	aph(3")-Ib, aph(6)-Id, catB9, dfrA1, sul2, tet(59)	Haitian gyrA Ser83 to Ile, ASP660 to Glu	4	ctxB_1	CTT	CTX-3	PLE1
	BGD083	2013	Clinical	This study	DRR335661	ICEtet	aph(3")-Ib, aph(6)-Id, catB9, dfrA1, sul2, tet(59)	Haitian gyrA Ser83 to Ile, ASP660 to Glu	5	ctxB_1	CTT	CTX-3	PLE1
	BGD084	2013	Clinical	Morita et al. 2020, mBio	DRX179784	ICEtet	aph(3")-Ib, aph(6)-Id, catB9, dfrA1, sul2, tet(59)	Haitian gyrA Ser83 to Ile, ASP660 to Glu	5	ctxB_1	CTT	CTX-3	PLE1
	BGD085	2014	Clinical	This study	DRR335662	ICEtet	aph(3")-Ib, aph(6)-Id, catB9, dfrA1, sul2, tet(59)	Haitian gyrA Ser83 to Ile, ASP660 to Glu	5	ctxB_1	CTT	CTX-3	PLE1
	BGD086	2014	Clinical	Morita et al. 2020, mBio	DRX179786	ICEtet	aph(3")-Ib, aph(6)-Id, catB9, dfrA1, sul2, tet(59)	Haitian gyrA Ser83 to Ile, ASP660 to Glu	5	ctxB_1	CTT	CTX-3	PLE1
	BGD087	2014	Clinical	Morita et al. 2020, mBio	DRX179787	ICEtet	aph(3")-Ib, aph(6)-Id, catB9, dfrA1, sul2, tet(59)	Haitian gyrA Ser83 to Ile, ASP660 to Glu	5	ctxB_1	CTT	CTX-3	PLE1
	BGD088	2014	Clinical	Morita et al. 2020, mBio	DRX179788	ICEtet	aph(3")-Ib, aph(6)-Id, catB9, dfrA1, sul2, tet(59)	Haitian gyrA Ser83 to Ile, ASP660 to Glu	5	ctxB_1	CTT	CTX-3	PLE1
	BGD089	2014	Clinical	Morita et al. 2020, mBio	DRX179789	ICEtet	aph(3")-Ib, aph(6)-Id, catB9, dfrA1, sul2, tet(59)	Haitian gyrA Ser83 to Ile, ASP660 to Glu	5	ctxB_1	CTT	CTX-3	PLE1
	BGD090	2014	Clinical	This study	DRR335663	ICEtet	aph(3")-Ib, aph(6)-Id, catB9, dfrA1, sul2, tet(59)	Haitian gyrA Ser83 to Ile, ASP660 to Glu	4	ctxB_1	CTT	CTX-3	PLE1
	BGD091	2014	Clinical	Morita et al. 2020, mBio	DRX179790	ICEtet	aph(3")-Ib, aph(6)-Id, catB9, dfrA1, sul2, tet(59)	Haitian gyrA Ser83 to Ile, ASP660 to Glu	5	ctxB_1	CTT	CTX-3	PLE1
	BGD098	2014	Clinical	Morita et al. 2020, mBio	DRX179795	ICEtet	aph(3")-Ib, aph(6)-Id, catB9, dfrA1, sul2, tet(59)	Haitian gyrA Ser83 to Ile, ASP660 to Glu	5	ctxB_1	CTT	CTX-3	PLE1

	BGD099	2014	Clinical	This study	DRR335666	ICEtet	aph(3")-Ib, aph(6)-Id, catB9, dfrA1, sul2, tet(59)	Haitian gyrA Ser83 to Ile, ASP660 to Glu	5	ctxB_1	CTT	CTX-3	PLE1
	MAB041	2014	Env	This study	DRR335725	ICEtet	aph(3")-Ib, aph(6)-Id, catB9, dfrA1, sul2, tet(59)	Haitian gyrA Ser83 to Ile, ASP660 to Glu	5	ctxB_1	CTT	CTX-3	PLE1
	BGD133	2015	No data	Morita et al. 2020, mBio	DRX179761	ICEtet	aph(3")-Ib, aph(6)-Id, catB9, dfrA1, sul2, tet(59)	Haitian gyrA Ser83 to Ile, ASP660 to Glu	4	ctxB_1	CTT	CTX-3	PLE(-)
	BGD134	2015	No data	This study	DRR335675	ICEtet	aph(3")-Ib, aph(6)-Id, catB9, dfrA1, sul2, tet(59)	Haitian gyrA Ser83 to Ile, ASP660 to Glu	3	ctxB_1	CTT	CTX-3	PLE1
	MAB003	2004	Clinical	This study	DRR335693	ICEtet	aph(3")-Ib, aph(6)-Id, catB9, dfrA1, sul2, tet(59)	Haitian gyrA Ser83 to Ile, ASP660 to Glu	4	ctxB_1	CTT	CTX-3	PLE1
	MAB014	2008	Clinical	This study	DRR335703	ICEtet	aph(3")-Ib, aph(6)-Id, catB9, dfrA1, sul2, tet(59)	Haitian gyrA Ser83 to Ile, ASP660 to Glu	4	ctxB_1	CTT	CTX-3	PLE1
	MAB015	2008	Clinical	This study	DRR335704	ICEtet	aph(3")-Ib, aph(6)-Id, catB9, dfrA1, sul2, tet(59)	Haitian gyrA Ser83 to Ile, ASP660 to Glu	4	ctxB_1	CTT	CTX-3	PLE(-)
	BGD117	2011	No data	Morita et al. 2020, mBio	DRX179750	ICEtet	aph(3")-Ib, aph(6)-Id, catB9, dfrA1, sul2, tet(59)	Haitian gyrA Ser83 to Ile, ASP660 to Glu	4	ctxB_1	CTT	CTX-3	PLE1
	MAB026	2011	Clinical	This study	DRR335712	ICEtet	aph(3")-Ib, aph(6)-Id, catB9, dfrA1, sul2, tet(59)	Haitian gyrA Ser83 to Ile, ASP660 to Glu	4	ctxB_1	CTT	CTX-3	PLE1
	MAB029	2011	Clinical	This study	DRR335715	ICEtet	aph(3")-Ib, aph(6)-Id, catB9, dfrA1, sul2, tet(59)	Haitian gyrA Ser83 to Ile, ASP660 to Glu	4	ctxB_1	CTT	CTX-3	PLE1
	BGD118	2012	No data	Morita et al. 2020, mBio	DRX179751	ICEtet	aph(3")-Ib, aph(6)-Id, catB9, dfrA1, sul2, tet(59)	Haitian gyrA Ser83 to Ile, ASP660 to Glu	4	ctxB_1	CTT	CTX-3	PLE(-)

	BGD119	2012	No data	Morita et al. 2020, mBio	DRX179752	ICEtet	aph(3")-Ib, aph(6)-Id, catB9, dfrA1, sul2, tet(59)	Haitian gyrA Ser83 to Ile, ASP660 to Glu	4	ctxB_1	CTT	CTX-3	PLE(-)
	BGD120	2012	No data	Morita et al. 2020, mBio	DRX179753	ICEtet	aph(3")-Ib, aph(6)-Id, catB9, dfrA1, sul2, tet(59)	Haitian gyrA Ser83 to Ile, ASP660 to Glu	3	ctxB_1	CTT	CTX-3	PLE(-)
	BGD121	2012	No data	Morita et al. 2020, mBio	DRX179754	ICEtet	aph(3")-Ib, aph(6)-Id, catB9, dfrA1, sul2, tet(59)	Haitian gyrA Ser83 to Ile, ASP660 to Glu	4	ctxB_1	CTT	CTX-3	PLE(-)
	MAB032	2012	Env	This study	DRR335717	ICEtet	aph(3")-Ib, aph(6)-Id, catB9, dfrA1, sul2, tet(59)	Haitian gyrA Ser83 to Ile, ASP660 to Glu	5	ctxB_1	CTT	CTX-3	PLE1
	MAB033	2012	Env	This study	DRR335718	ICEtet	aph(3")-Ib, aph(6)-Id, catB9, dfrA1, sul2, tet(59)	Haitian gyrA Ser83 to Ile, ASP660 to Glu	5	ctxB_1	CTT	CTX-3	PLE1
	MAB034	2012	Env	This study	DRR335719	ICEtet	aph(3")-Ib, aph(6)-Id, catB9, dfrA1, sul2, tet(59)	Haitian gyrA Ser83 to Ile, ASP660 to Glu	4	ctxB_1	CTT	CTX-3	PLE1
	MAB038	2012	Clinical	This study	DRR335722	ICEtet	aph(3")-Ib, aph(6)-Id, catB9, dfrA1, sul2, tet(59)	Haitian gyrA Ser83 to Ile, ASP660 to Glu	4	ctxB_1	CTT	CTX-3	PLE1
	BGD076	2013	Clinical	Morita et al. 2020, mBio	DRX179782	ICEtet	aph(3")-Ib, aph(6)-Id, catB9, dfrA1, sul2, tet(59)	Haitian gyrA Ser83 to Ile, ASP660 to Glu	4	ctxB_1	CTT	CTX-3	PLE1
	BGD081	2013	Clinical	This study	DRR335660	ICEtet	aph(3")-Ib, aph(6)-Id, catB9, dfrA1, sul2, tet(59)	Haitian gyrA Ser83 to Ile, ASP660 to Glu	4	ctxB_1	CTT	CTX-3	PLE1
	BGD122	2013	No data	Morita et al. 2020, mBio	DRX179755	ICEtet	aph(3")-Ib, aph(6)-Id, catB9, dfrA1, sul2, tet(59)	Haitian gyrA Ser83 to Ile, ASP660 to Glu	4	ctxB_1	CTT	CTX-3	PLE(-)
	MAB040	2013	Env	This study	DRR335724	ICEtet	aph(3")-Ib, aph(6)-Id, catB9, dfrA1, sul2, tet(59)	Haitian gyrA Ser83 to Ile, ASP660 to Glu	4	ctxB_1	CTT	CTX-3	PLE1

	BGD093	2014	Clinical	This study	DRR335664	ICEtet	aph(3")-Ib, aph(6)-Id, catB9, dfrA1, sul2, tet(59)	Haitian gyrA Ser83 to Ile, ASP660 to Glu	4	ctxB_1	CTT	CTX-3	PLE1
	BGD094	2014	Clinical	Morita et al. 2020, mBio	DRX179792	ICEtet	aph(3")-Ib, aph(6)-Id, catB9, dfrA1, sul2, tet(59)	Haitian gyrA Ser83 to Ile, ASP660 to Glu	4	ctxB_1	CTT	CTX-3	PLE1
	BGD095	2014	Clinical	Morita et al. 2020, mBio	DRX179793	ICEtet	aph(3")-Ib, aph(6)-Id, catB9, dfrA1, sul2, tet(59)	Haitian gyrA Ser83 to Ile, ASP660 to Glu	4	ctxB_1	CTT	CTX-3	PLE1
	BGD096	2014	Clinical	Morita et al. 2020, mBio	DRX179794	ICEtet	aph(3")-Ib, aph(6)-Id, catB9, dfrA1, sul2, tet(59)	Haitian gyrA Ser83 to Ile, ASP660 to Glu	4	ctxB_1	CTT	CTX-3	PLE1
	BGD097	2014	Clinical	This study	DRR335665	ICEtet	aph(3")-Ib, aph(6)-Id, catB9, dfrA1, sul2, tet(59)	Haitian gyrA Ser83 to Ile, ASP660 to Glu	4	ctxB_1	CTT	CTX-3	PLE1
	BGD124	2014	No data	Morita et al. 2020, mBio	DRX179756	ICEtet	aph(3")-Ib, aph(6)-Id, catB9, dfrA1, sul2, tet(59)	Haitian gyrA Ser83 to Ile, ASP660 to Glu	5	ctxB_1	CTT	CTX-3	PLE1
	BGD127	2015	No data	This study	DRR335673	Untypeable	catB9	Haitian gyrA Ser83 to Ile, ASP660 to Glu	4	ctxB_1	CTT	CTX-3	PLE1
	BGD128	2015	No data	Morita et al. 2020, mBio	DRX179758	ICEtet	aph(3")-Ib, aph(6)-Id, catB9, dfrA1, sul2, tet(59)	Haitian gyrA Ser83 to Ile, ASP660 to Glu	4	ctxB_1	CTT	CTX-3	PLE1
	BGD130	2015	No data	Morita et al. 2020, mBio	DRX179759	ICEtet	aph(3")-Ib, aph(6)-Id, catB9, dfrA1, sul2, tet(59)	Haitian gyrA Ser83 to Ile, ASP660 to Glu	4	ctxB_1	CTT	CTX-3	PLE1
	BGD131	2015	No data	This study	DRR335674	ICEtet	aph(3")-Ib, aph(6)-Id, catB9, dfrA1, sul2, tet(59)	Haitian gyrA Ser83 to Ile, ASP660 to Glu	4	ctxB_1	CTT	CTX-3	PLE1
	BGD138	2016	No data	Morita et al. 2020, mBio	DRX179764	Untypeable	catB9	Haitian gyrA Ser83 to Ile, ASP660 to Glu	4	ctxB_1	CTT	CTX-3	PLE1

	MAB004	2005	Env	This study	DRR335694	ICEtet	aph(3")-Ib, aph(6)-Id, catB9, dfrA1, dfrA31, mph(A), qnrVC1, sul2, tet(59)	Haitian gyrA Ser83 to Ile	4	ctxB_1	CTT	CTX-3	PLE(-)
	MAB006	2005	Clinical	This study	DRR335696	ICEtet	aph(3")-Ib, aph(6)-Id, catB9, dfrA1, dfrA31, mph(A), qnrVC1, sul2, tet(59)	Haitian gyrA Ser83 to Ile	4	ctxB_1	CTT	CTX-3	PLE(-)
	MAB008	2006	Env	This study	DRR335698	ICEtet	catB9, dfrA1, dfrA31, qnrVC1	Haitian gyrA Ser83 to Ile	4	ctxB_1	CTT	CTX-3	PLE(-)
	BGD005	2007	Clinical	This study	DRR335608	ICEtet	aph(3")-Ib, aph(6)-Id, catB9, dfrA1, dfrA31, qnrVC1, sul2, tet(59)	Haitian gyrA Ser83 to Ile	4	ctxB_1	CTT	CTX-3	PLE(-)
	MAB010	2007	Env	This study	DRR335613	ICEtet	aph(3")-Ib, aph(6)-Id, catB9, dfrA1, dfrA31, qnrVC1, sul2, tet(59)	Haitian gyrA Ser83 to Ile	3	ctxB_1	CTT	CTX-3	PLE(-)
	BGD026	2009	Clinical	Morita et al. 2020, mBio	DRX179772	ICEtet	aph(3")-Ib, aph(6)-Id, catB9, dfrA1, dfrA31, qnrVC1, sul2, tet(59)	Haitian gyrA Ser83 to Ile, ASP660 to Glu	4	ctxB_1	CTT	CTX-3	PLE1
	BGD029	2009	Clinical	This study	DRR335628	ICEtet	aph(3")-Ib, aph(6)-Id, catB9, dfrA1, dfrA31, qnrVC1, sul2, tet(59)	Haitian gyrA Ser83 to Ile, ASP660 to Glu	4	ctxB_1	CTT	CTX-3	PLE1
	BGD030	2009	Clinical	This study	DRR335629	ICEtet	aph(3")-Ib, aph(6)-Id, catB9, dfrA1, dfrA31, qnrVC1, sul2, tet(59)	Haitian gyrA Ser83 to Ile, ASP660 to Glu	4	ctxB_1	CTT	CTX-3	PLE1
	BGD031	2009	Clinical	This study	DRR335630	ICEtet	catB9, dfrA1, dfrA31, qnrVC1	Haitian gyrA Ser83 to Ile, ASP660 to Glu	4	ctxB_1	CTT	CTX-3	PLE(-)
	BGD032	2009	Clinical	This study	DRR335631	ICEtet	aph(3")-Ib, aph(6)-Id, catB9, dfrA1, dfrA31, qnrVC1, sul2, tet(59)	Haitian gyrA Ser83 to Ile, ASP660 to Glu	4	ctxB_1	CTT	CTX-3	PLE1
	BGD033	2009	Clinical	This study	DRR335632	ICEtet	aph(3")-Ib, aph(6)-Id, catB9, dfrA1, dfrA31, qnrVC1, sul2, tet(59)	Haitian gyrA Ser83 to Ile, ASP660 to Glu	4	ctxB_1	CTT	CTX-3	PLE1
	BGD034	2009	Clinical	This study	DRR335633	Untypeable	catB9	Haitian gyrA Ser83 to Ile, ASP660 to Glu	4	ctxB_1	CTT	CTX-3	PLE(-)

	BGD035	2009	Clinical	This study	DRR335634	ICEtet	aph(3")-Ib, aph(6)-Id, catB9, dfrA1, dfrA31, qnrVC1, sul2, tet(59)	Haitian gyrA Ser83 to Ile, ASP660 to Glu	3	ctxB_1	CTT	CTX-3	PLE1
	BGD104	2009	No data	Morita et al. 2020, mBio	DRX179741	ICEtet	aph(3")-Ib, aph(6)-Id, catB9, dfrA1, dfrA31, sul2, tet(59)	Haitian gyrA Ser83 to Ile, ASP660 to Glu	4	ctxB_1	CTT	CTX-3	PLE1
	BGD105	2009	No data	Morita et al. 2020, mBio	DRX179742	ICEtet	aph(3")-Ib, aph(6)-Id, catB9, dfrA1, sul2, tet(59)	Haitian gyrA Ser83 to Ile, ASP660 to Glu	4	ctxB_1	CTT	CTX-3	PLE1
	BGD106	2009	No data	This study	DRR335667	ICEtet	aph(3")-Ib, aph(6)-Id, catB9, dfrA1, dfrA31, qnrVC1, sul2, tet(59)	Haitian gyrA Ser83 to Ile, ASP660 to Glu	4	ctxB_1	CTT	CTX-3	PLE1
	BGD108	2009	No data	This study	DRR335668	ICEtet	aph(3")-Ib, aph(6)-Id, catB9, dfrA1, dfrA31, qnrVC1, sul2, tet(59)	Haitian gyrA Ser83 to Ile, ASP660 to Glu	4	ctxB_1	CTT	CTX-3	PLE1
	BGD109	2009	No data	Morita et al. 2020, mBio	DRX179744	ICEtet	catB9, dfrA1, dfrA31, qnrVC1	Haitian gyrA Ser83 to Ile, ASP660 to Glu	3	ctxB_1	CTT	CTX-3	PLE(-)
	MAB017	2009	Clinical	This study	DRR335705	ICEtet	aph(3")-Ib, aph(6)-Id, catB9, dfrA1, dfrA31, qnrVC1, sul2, tet(59)	Haitian gyrA Ser83 to Ile	3	ctxB_1	CTT	CTX-3	PLE(-)
	BGD046	2010	Clinical	This study	DRR335643	ICEtet	aph(3")-Ib, aph(6)-Id, catB9, dfrA1, dfrA31, qnrVC1, sul2, tet(59)	Haitian gyrA Ser83 to Ile, ASP660 to Glu	4	ctxB_1	CTT	CTX-3	PLE1
	MAB022	2010	Clinical	This study	DRR335709	ICEtet	aph(3")-Ib, aph(6)-Id, catB9, dfrA1, dfrA31, qnrVC1, sul2, tet(59)	Haitian gyrA Ser83 to Ile, ASP660 to Glu	4	ctxB_1	CTT	CTX-3	PLE1
	MAB023	2011	Env	This study	DRR335710	ICEtet	aph(3")-Ib, aph(6)-Id, dfrA1, sul2, tet(59)	Haitian gyrA Ser83 to Ile, ASP660 to Glu	4	ctxB_1	CTT	CTX-3	PLE1
	MAB025	2011	Env	This study	DRR335711	ICEtet	aph(3")-Ib, aph(6)-Id, dfrA1, sul2, tet(59)	Haitian gyrA Ser83 to Ile, ASP660 to Glu	4	ctxB_1	CTT	CTX-3	PLE1
	MAB027	2011	Env	This study	DRR335713	ICEtet	aph(3")-Ib, aph(6)-Id, dfrA1, dfrA31,	Haitian gyrA Ser83 to Ile,	4	ctxB_1	CTT	CTX-3	PLE1

							qnrVC1, sul2, tet(59)	ASP660 to Glu					
	MAB028	2011	Env	This study	DRR335714	ICEtet	aph(3")-Ib, aph(6)-Id, dfrA1, sul2, tet(59)	Haitian gyrA Ser83 to Ile, ASP660 to Glu	4	ctxB_1	CTT	CTX-3	PLE1
	MAB030	2011	Clinical	This study	DRR335716	ICEtet	catB9, dfrA1, dfrA31, qnrVC1	Haitian gyrA Ser83 to Ile, ASP660 to Glu	4	ctxB_1	CTT	CTX-3	PLE(-)

Lineage refers to nearly genetically homogeneous groups of strains. Legends are strain ID, year of isolation, SXT/ICE elements, acquired antibiotic resistance profile, *gyrA* allele, number of ToxR binding repeats, *ctxB* allele, and PLE are tabulated. The alleles of *rstA*, and *ctxB* were used to type CTX prophage. *rstA* allele CTT refers T927C, C933T, and G942T; and allele TTAC refers C27T, C162T, C183A, and G258C.

Table S2. Number of strains belonging to the different lineages

Year	N_BD-0	N_BD-1	N_BD-2	Total
1991	3			3
1994	4			4
1999		1		1
2000	1			1
2001		4		4
2004		2	1	3
2005		2	2	4
2006		2	1	3
2007		16	2	18
2008		9	2	11
2009		1	14	15
2010		19	3	22
2011		10	8	18
2012	3	7	8	18
2013			18	18
2014		1	16	17
2015		1	8	9
2016		1	11	12
2017			11	11

Here, N_BD-0 = number of strains belonging to BD-0; N_BD-1 = number of strains belonging to BD-1; and N_BD2 = number of strains belonging to BD-2.

Table S3. Fisher exact test identifying significantly associated 140 SNPs and 31 indels of the two dominant lineages, BD-1 and BD-2

SNP/indel	Ref	Alt1	Alt2	P-value	Variant type	Amino acid change	Gene/region name	Product
S1_2200070	TC	T	-	5.61E-53	frameshift_variant	Asp163fs	acuC	Acetoin utilization protein AcuC
S1_1905668	C	A	-	5.61E-53	missense_variant	Glu7Asp	ASZ87_00023	hypothetical protein
S1_1622584	G	A	-	5.61E-53	missense_variant	Pro50Leu	cobB	NAD-dependent protein deacylase
S1_1782501	G	A	-	5.61E-53	missense_variant	Leu79Phe	cph2_4	Phytochrome-like protein cph2
S2_921045	A	C	-	5.61E-53	missense_variant	Ile161Ser	ctpH_6	Methyl-accepting chemotaxis protein CtpH
S1_1452755	T	C	-	5.61E-53	missense_variant	Val38Ala	cysG_1	Siroheme synthase
S1_2078472	C	T	-	5.61E-53	synonymous_variant	Ile312Ile	dctD_1	C4-dicarboxylate transport transcriptional regulatory protein DctD
S1_1118265	G	A	-	5.61E-53	synonymous_variant	Glu688Glu	fadJ	Fatty acid oxidation complex subunit alpha
S1_681574	G	T	-	5.61E-53	missense_variant	Arg196Leu	glmM	Phosphoglucosamine mutase
S2_839769	A	G	-	5.61E-53	synonymous_variant	Gly117Gly	kbl	2-amino-3-ketobutyrate coenzyme A ligase
S1_276112	G	A	-	5.61E-53	missense_variant	Gly116Arg	mak	Fructokinase
S1_1961274	C	T	-	5.61E-53	synonymous_variant	Asp59Asp	manP_1	PTS system mannose-specific EIIBCA component
S1_2609994	G	A	-	5.61E-53	missense_variant	Arg109Cys	nudF_1	ADP-ribose pyrophosphatase
S2_773493	T	A	-	5.61E-53	missense_variant	Gln19Leu	phhA	Phenylalanine-4-hydroxylase
S2_564621	C	T	-	5.61E-53	synonymous_variant	Leu275Leu	VCA0628	SecA-like protein
S2_1024884	G	A	-	5.61E-53	missense_variant	Ala600Val	putA	Bifunctional protein PutA
S1_2468430	T	A	-	5.61E-53	synonymous_variant	Ala69Ala	recD	RecBCD enzyme subunit RecD
S1_2551492	GAAAAAAAA	GAAAAAAA	-	5.61E-53	frameshift_variant	Asn296fs	recF_2	DNA replication and repair protein RecF
S2_161094	T	G	-	5.61E-53	missense_variant	Ser241Ala	siaT_5	Sialic acid TRAP transporter permease protein SiaT
S1_994229	G	A	-	5.61E-53	missense_variant	Gly201Asp	stcE_2	Metalloprotease StcE precursor
S1_798976	T	C	-	5.61E-53	missense_variant	Glu217Gly	suhB	Inositol-1-monophosphatase
S1_2731709	G	A	-	5.61E-53	missense_variant	Thr266Ile	tamA	Translocation and assembly module TamA precursor
S1_1563748	G	A	-	5.61E-53	stop_gained	Trp4534*	toxA	Toxin A

S2_266019	A	G	-	5.61E-53	missense_variant	Ile354Thr	ulaA	Ascorbate-specific permease IIC component UlaA
S1_200922	GAAAAAAA	GAAAAAAAA	-	5.61E-53	frameshift_variant	Gln248fs	VC0195	RarD protein
S2_333875	G	C	-	5.61E-53	upstream_gene_variant		VC0320	pantothenate kinase
S2_333877	T	A	-	5.61E-53	upstream_gene_variant		VC0320	pantothenate kinase
S2_333886	A	G	-	5.61E-53	upstream_gene_variant		VC0320	pantothenate kinase
S2_422960	C	T	-	5.61E-53	synonymous_variant	Gly59Gly	VC0397	single-stranded DNA-binding protein
S1_991447	A	G	-	5.61E-53	missense_variant	Met103Val	VC0928	hypothetical protein
S1_1344013	C	T	-	5.61E-53	missense_variant	Pro326Ser	VC1268	hypothetical protein
S1_1693044	G	A	-	5.61E-53	missense_variant	Asp98Asn	VC1582	hypothetical protein
S1_2195049	C	A	-	5.61E-53	missense_variant	Gly277Val	VC2038	hypothetical protein
S1_2317938	C	T	-	5.61E-53	upstream_gene_variant		VC2172	hypothetical protein
S1_2609364	C	A	-	5.61E-53	missense_variant	Asp108Tyr	VC2434	hypothetical protein
S2_989172	C	T	-	5.61E-53	missense_variant	Pro191Ser	yecS	Inner membrane amino-acid ABC transporter permease protein YecS
S1_2241989	A	G	-	5.61E-53	synonymous_variant	Val153Val	znuC	Zinc import ATP-binding protein ZnuC
S1_545919	T	G	-	4.32E-51	missense_variant	Leu249Trp	pctB_1	Methyl-accepting chemotaxis protein PctB
S1_906261	ATTTTTTTT	ATTTTTTTTT	-	4.32E-51	upstream_gene_variant		VC0845	accessory colonization factor AcfD
S1_2424759	C	T	-	5.94E-51	synonymous_variant	Leu186Leu	ribE_2	Riboflavin synthase
S1_2203897	C	T	-	5.94E-51	synonymous_variant	Val3Val	VC2046	hypothetical protein
S1_676136	GTTTTTTTT	GTTTTTTTTT	-	1.68E-49	upstream_gene_variant		mepM_2	Murein DD-endopeptidase MepM
S1_911354	GTTTTTTTT	GTTTTTTT	GTTTTTTTTT	1.68E-49	upstream_gene_variant		pasT	Persistence and stress-resistance toxin PasT
S1_799723	C	T	-	3.18E-49	upstream_gene_variant		suhB	Inositol-1-monophosphatase
S1_1418798	G	T	-	3.18E-49	upstream_gene_variant		VC1335	GntR family transcriptional regulator
S1_2669927	G	T	-	3.18E-49	upstream_gene_variant		VC2490	2-isopropylmalate synthase
S1_72585	G	A	-	3.18E-49	synonymous_variant	Lys354Lys	yegE_1	putative diguanylate cyclase YegE
S1_2814292	T	C	-	4.43E-48	missense_variant	Thr283Ala	argG	Argininosuccinate synthase
S2_737696	A	G	-	4.43E-48	synonymous_variant	Gln51Gln	VCA0794	hypothetical protein

S2_413076	G	A	-	1.44E-45	missense_variant	Asp39Asn	VCA0464	hypothetical protein
S1_1808852	G	A	-	6.86E-45	synonymous_variant	Ala18Ala	czcA	Cobalt-zinc-cadmium resistance protein CzcA
S2_414464	A	C	-	6.86E-45	upstream_gene_variant		higB-2	Toxin HigB-2
S1_1060408	C	T	-	6.86E-45	missense_variant	Asp150Asn	nagA_1	N-acetylglucosamine-6-phosphate deacetylase
S2_42229	C	A	-	6.86E-45	missense_variant	Gln272His	VCA0034	hypothetical protein
S1_881243	CTT	CTTT	-	8.60E-45	frameshift_variant	Leu373fs	VC0821	pseudogene
S1_1332186	T	G	-	1.96E-44	missense_variant	Asp660Glu	gyrA	DNA gyrase subunit A
S1_149686	G	T	-	1.96E-44	missense_variant	Ala137Ser	murI	Glutamate racemase
S2_408488	T	A	-	1.96E-44	upstream_gene_variant		VCA0458	hypothetical protein
S2_408508	A	G	-	1.96E-44	upstream_gene_variant		VCA0458	hypothetical protein
S2_408509	C	G	-	1.96E-44	upstream_gene_variant		VCA0458	hypothetical protein
S2_408510	G	C	-	1.96E-44	upstream_gene_variant		VCA0458	hypothetical protein
S2_562858	A	T	-	1.96E-44	missense_variant	Thr6Ser	VCA0627	rRNA methylase
S1_540849	TCCC	TCC	-	2.19E-44	frameshift_variant	Ser75fs	tnsB	Transposon Tn7 transposition protein TnsB
S1_876929	TAAAAAAA	TAAAAAA	-	2.20E-44	upstream_gene_variant		VC0820	ToxR-activated gene A protein
S1_543504	GA	GACTACA	GCCACTACA	1.27E-43	upstream_gene_variant		VC0513	AraC family transcriptional regulator
S2_408511	C	G	-	2.33E-43	upstream_gene_variant		VC0389	hypothetical protein
S1_881352	C	A	-	1.99E-40	upstream_gene_variant		aldB_1	Aldehyde dehydrogenase B
S1_1682925	C	T	-	3.63E-40	missense_variant	Ala226Thr	appC	Cytochrome bd-II ubiquinol oxidase subunit 1
S1_137214	T	C	-	3.63E-40	synonymous_variant	Ser87Ser	ftsY	Signal recognition particle receptor FtsY
S1_1359179	G	A	-	3.63E-40	missense_variant	Ala56Thr	licH	putative 6-phospho-beta-glucosidase
S1_368119	T	C	-	3.63E-40	missense_variant	Cys350Arg	mutL	DNA mismatch repair protein MutL
S1_903579	CAAAAAAAAA	CAAAAAAAA	-	3.63E-40	frameshift_variant	Asn67fs	VC0841	hypothetical protein
S1_1739750	C	T	-	3.63E-40	missense_variant	Val540Ile	VC1620	hypothetical protein
S1_2059741	C	T	-	3.63E-40	missense_variant	Ala175Val	VC1909	hypothetical protein
S2_685842	G	T	-	3.63E-40	stop_gained	Ser32*	VCA0739	hypothetical protein
S1_2483236	G	A	-	4.18E-39	missense_variant	Ala150Thr	lysX	Alpha-aminoadipate--LysW ligase LysX

S2_704891	G	A	-	4.18E-39	upstream_gene_variant		VCA0762	hypothetical protein
S1_543484	T	C	-	4.30E-37	upstream_gene_variant		VC0513	AraC family transcriptional regulator
S1_906290	TAAAAA	TAAAAAA	-	4.30E-37	upstream_gene_variant		VC0845	hypothetical protein
S2_333839	A	G	-	1.78E-35	upstream_gene_variant		VCA0332	hypothetical protein
S1_628646	C	T	-	1.32E-32	missense_variant	Ala782Val	hrpB_1	ATP-dependent RNA helicase HrpB
S1_673206	A	G	-	1.32E-32	missense_variant	Thr393Ala	tyrS_2	Tyrosine--tRNA ligase
S1_1584509	G	GAGACTGTAAGATC	-	6.42E-31	upstream_gene_variant		VC1479	hypothetical protein
S1_1584755	T	C	-	1.88E-30	upstream_gene_variant		VC1479	hypothetical protein
S1_744709	G	A	-	4.01E-30	synonymous_variant	Val30Val	tyrA	T-protein
S1_2357516	G	A	-	7.24E-29	missense_variant	Leu227Phe	angR	Anguibactin system regulator
S1_524675	CTTTTTTT	CTTTTTT	-	7.24E-29	frameshift_variant	Arg148fs	VC0490	hypothetical protein
S1_1878672	G	A	-	7.24E-29	upstream_gene_variant		VC1743	hypothetical protein
S2_306422	G	A	-	7.24E-29	upstream_gene_variant		thrS	Threonine--tRNA ligase
S1_1824680	GAGATGACTAGATGACTAGATGACT	GAGATGACTAGATGACT	-	7.24E-29	frameshift_variant&stop_lost&splice_region_variant	Val819fs	torA	Trimethylamine-N-oxide reductase precursor
S2_1050706	GTTGACCAT	G	-	1.39E-28	frameshift_variant	Asp576fs	cheA_3	Chemotaxis protein CheA
S1_1567284	G	T	-	1.39E-28	missense_variant	His20Asn	ctxB	Cholera enterotoxin subunit B precursor
S2_203369	G	T	-	1.39E-28	upstream_gene_variant		luxO_2	Regulatory protein LuxO
S1_2690493	G	A	-	1.39E-28	missense_variant	Arg599His	valS	Valine--tRNA ligase
S1_1970973	A	G	-	7.73E-28	synonymous_variant	Gly61Gly	alaE	L-alanine exporter AlaE
S1_2191730	A	G	-	7.73E-28	missense_variant	Val347Ala	asd1	Aspartate-semialdehyde dehydrogenase 1
S1_2240207	C	T	-	7.73E-28	missense_variant	Gly52Arg	cdhR	HTH-type transcriptional regulator CdhR
S1_1154205	GAACTATCTCCATAACAAA	GAACTATCTCCATAA	-	7.73E-28	frameshift_variant	Leu516fs	cph2_3	Phytochrome-like protein cph2

	CTATCTC<br>CATAA							
S1_2085882	C	T	-	7.73E-28	missense_variant	Ala340Val	cph2_6	Phytochrome-like protein cph2
S1_1812498	C	T	-	7.73E-28	upstream_gene_variant		czcA	Cobalt-zinc-cadmium resistance protein CzcA
S1_831779	C	A	-	7.73E-28	missense_variant	Ala170Ser	dhbF	Dimodular nonribosomal peptide synthase
S1_1117838	T	G	-	7.73E-28	missense_variant	Val546Gly	fadJ	Fatty acid oxidation complex subunit alpha
S2_990917	GCCCC	GCCC	-	7.73E-28	frameshift_variant	Pro217fs	glnP	Glutamine transport system permease protein GlnP
S2_609892	G	A	-	7.73E-28	missense_variant	Arg532Cys	narX	Nitrate/nitrite sensor protein NarX
S1_1354024	G	A	-	7.73E-28	missense_variant	Ala136Thr	opuD	Glycine betaine transporter OpuD
S1_2485904	G	A	-	7.73E-28	missense_variant	Ala158Thr	VC2335	hypothetical protein
S2_568477	C	T	-	7.73E-28	missense_variant	Arg106His	VCA0631	hypothetical protein
S1_1778509	CGATAA<br>TCCAGAT<br>AATCCA<br>GATAAT<br>CCAGAT<br>AATCCA<br>GATAAT<br>CCAGAT<br>AATCCA<br>GATAAT<br>CCAGA	CGATA<br>ATCCA<br>GATAA<br>TCCAG<br>ATAAT<br>CCAGA<br>TAATC<br>CAGAT<br>AATCC<br>AGATA<br>ATCCA<br>GA	-	7.73E-28	disruptive_inframe_deletion	Asp583_Pro5<br>85del	prt	Microbial collagenase precursor
S1_2476906	G	A	-	7.73E-28	missense_variant	Ala91Val	tehA	Tellurite resistance protein TehA
S1_532426	C	G	-	1.18E-27	upstream_gene_variant		VC0501	pseudogene
S1_532427	T	A	-	1.18E-27	upstream_gene_variant		VC0501	pseudogene
S1_532430	G	T	A	1.18E-27	upstream_gene_variant		VC0501	pseudogene
S1_532440	T	C	-	1.18E-27	upstream_gene_variant		VC0501	pseudogene
S1_532441	C	T	-	1.18E-27	upstream_gene_variant		VC0501	pseudogene
S1_532443	G	A	-	1.18E-27	upstream_gene_variant		VC0501	pseudogene
S1_532447	G	A	-	1.18E-27	upstream_gene_variant		VC0501	pseudogene
S2_253943	T	C	-	5.21E-27	synonymous_variant	Ile110Ile	bhuA	Heme transporter BhuA precursor

S1_2698225	C	T	-	5.21E-27	upstream_gene_variant		rluA_1	Ribosomal large subunit pseudouridine synthase A
S1_532409	C	T	-	4.63E-26	upstream_gene_variant		VC0501	pseudogene
S1_532411	T	A	-	4.63E-26	upstream_gene_variant		VC0501	pseudogene
S1_532412	G	C	-	4.63E-26	upstream_gene_variant		VC0501	pseudogene
S2_239068	TAACAAC	TAAC	-	6.08E-25	conservative_inframe_deletion	Asn495del	hlyA_3	Hemolysin precursor
S1_653278	C	T	-	6.08E-25	synonymous_variant	Ala63Ala	oppD_1	Oligopeptide transport ATP-binding protein OppD
S2_238321	CATTCATCTGAATGATCAA	CA	-	2.38E-24	frameshift_variant	Ile245fs	hlyA_3	Hemolysin precursor
S2_343197	C	T	-	2.53E-24	upstream_gene_variant		blc_2	Outer membrane lipoprotein Blc precursor
S1_532398	G	A	-	1.65E-23	upstream_gene_variant		VC0501	pseudogene
S1_532400	T	G	-	1.65E-23	upstream_gene_variant		VC0501	pseudogene
S2_411337	GTT	GTGGGTAAGTAAAGCTATT	-	5.29E-23	upstream_gene_variant		VCA0463	biphenyl-2%2C3-diol 1%2C2-dioxygenase
S1_137105	TAACAGAAACAGAAACAGAAACAGAAACAGAAACAGAAACAGAAACAGAAACAGAAACAG	TAACAGAAACAGAAACAGAAACAGAAACAGAAACAGAAACAGAAACAGAAACAGAAACAG	TAACAGAAACAGAAACAGAAACAGAAACAGAAACAGAAACAGAAACAGAAACAGAAACAGAAACAG	1.14E-22	disruptive_inframe_insertion	Glu67_Thr70dup	ftsY	Signal recognition particle receptor FtsY
S1_875131	T	A	-	8.67E-19	upstream_gene_variant		tehB	Tellurite methyltransferase

S1_875132	T	G	-	8.67E-19	upstream_gene_variant		tehB	Tellurite methyltransferase
S1_875133	A	G	-	8.67E-19	upstream_gene_variant		tehB	Tellurite methyltransferase
S1_875117	T	TCCG	TCCGAGATTCCGC	7.02E-18	upstream_gene_variant		tehB	Tellurite methyltransferase
S1_532469	ATG	A	-	2.71E-17	upstream_gene_variant		VC0501	pseudogene
S2_333857	A	T	-	3.17E-14	upstream_gene_variant		VCA0332	hypothetical protein
S2_407637	CA	CAAATAAAGGGGA	-	3.17E-14	upstream_gene_variant		VCA0457	hypothetical protein
S2_331952	G	A	-	8.01E-14	upstream_gene_variant		yafQ	mRNA interferase YafQ
S1_2787854	T	C	-	5.43E-13	upstream_gene_variant		VC2619	para-aminobenzoate synthase component II
S1_1904599	T	G	-	8.16E-12	missense_variant	Asn364His	VC1764	hypothetical protein
S2_256217	G	A	-	8.16E-12	upstream_gene_variant		soxS	Regulatory protein SoxS
S1_2910363	A	G	-	8.17E-12	missense_variant	Lys117Glu	pckA	Phosphoenolpyruvate carboxykinase [ATP]
S1_543498	G	C	-	2.50E-11	upstream_gene_variant		VC0513	AraC family transcriptional regulator
S1_2753124	G	A	-	4.41E-11	synonymous_variant	Arg13Arg	rpsD	30S ribosomal protein S4
S1_2312756	A	C	G	4.41E-11	synonymous_variant	Ala159Ala	yfgC_1	TPR repeat-containing protein YfgC precursor
S1_2858948	G	A	-	4.41E-11	upstream_gene_variant		zapB	Cell division protein ZapB
S2_333832	T	C	-	1.05E-10	upstream_gene_variant		VCA0332	hypothetical protein
S2_333834	T	C	-	1.05E-10	upstream_gene_variant		VCA0332	hypothetical protein
S1_1993908	TAAAAAAAAA	TAAAAAAAA	-	1.81E-10	upstream_gene_variant		VC1855	ATP-dependent DNA helicase DinG
S1_543496	G	T	-	2.25E-10	upstream_gene_variant		VC0513	AraC family transcriptional regulator
S2_333835	T	G	-	2.52E-10	upstream_gene_variant		VCA0332	hypothetical protein
S1_228348	C	T	-	3.09E-10	missense_variant	Ala140Thr	coaD	Phosphopantetheine adenylyltransferase
S2_333809	C	T	-	3.09E-10	upstream_gene_variant		VCA0332	hypothetical protein
S2_403580	AAAT	AAATGTATAGAACCAAGATAAT	-	6.56E-10	stop_gained&conservative_inframe_insertion	Asn139_Lys140insValTerAsnGlnAspAsn	VCA0451	hypothetical protein
S1_114836	G	A	-	1.93E-09	synonymous_variant	Gly287Gly	hemC	Porphobilinogen deaminase

S1_543493	A	C	-	1.93E-09	upstream_gene_variant		VC0513	AraC family transcriptional regulator
S1_543494	A	T	-	1.93E-09	upstream_gene_variant		VC0513	AraC family transcriptional regulator
S1_384096	C	T	-	1.93E-09	upstream_gene_variant		rpsF	30S ribosomal protein S6
S1_467110	CGACCCTAGACCCTAGACCCTAGACCCTAGACCCTAGACCCTAGACCCTAGACC	CGACCCTAGACCCTAGACCCTAGACC	CGACCCTAGACCCTAGACCCTAGACCCTAGACCCTAGACCCTAGACC	2.23E-09	upstream_gene_variant		ispB	Octaprenyl-diphosphate synthase
S1_1564656	C	T	-	2.98E-09	synonymous_variant	Lys62Lys	VC1453	RstB1 protein
S1_1564740	T	C	-	2.98E-09	synonymous_variant	Val34Val	VC1453	RstB1 protein
S1_1564752	G	A	-	2.98E-09	synonymous_variant	Tyr30Tyr	VC1453	RstB1 protein
S1_1564758	A	T	-	2.98E-09	synonymous_variant	Ala28Ala	VC1453	RstB1 protein
S2_333813	A	G	-	2.98E-09	upstream_gene_variant		VCA0332	hypothetical protein
S2_331943	CTT	CCGTCATCAATAAACAAAGTTT	-	2.98E-09	upstream_gene_variant		yafQ	mRNA interferase YafQ
S1_1564768	TTACT	TTACTACT	-	6.40E-09	conservative_inframe_insertion	Val23_Ser24insSer	VC1453	RstB1 protein
S2_333820	C	G	T	6.40E-09	upstream_gene_variant		VCA0332	hypothetical protein
S2_333822	A	C	-	6.40E-09	upstream_gene_variant		VCA0332	hypothetical protein
S2_333823	T	C	-	6.40E-09	upstream_gene_variant		VCA0332	hypothetical protein
S2_333824	G	C	-	6.40E-09	upstream_gene_variant		VCA0332	hypothetical protein
S2_333825	C	A	-	6.40E-09	upstream_gene_variant		VCA0332	hypothetical protein
S2_333826	G	A	-	6.40E-09	upstream_gene_variant		VCA0332	hypothetical protein

SNP/indel indicates significant SNP/indels identified by Fisher exact test. SNPs and indels named according to chromosomal position. For example, "S1 1905668" is an SNP/indel site, where "S" stands for site and "1905668" stands for location for site base pair. Reference allele is Ref, where as the alternative alleles are Alt1 and Alt2. P-value is Fisher exact test value. Variant type indicates SNP and indel type.

Table S4. Roary pangenome analysis showing gene compositions differences by lineage

Gene cluster	N_BD-0	N_BD-1	N_BD-2
Core genes (99% <= strains <= 100%)	1112	1882	3214
Soft core genes (95% <= strains < 99%)	0	1481	142
Shell genes (15% <= strains < 95%)	3076	244	355
Cloud genes (0% <= strains < 15%)	1993	1751	3499
Total genes (0% <= strains <= 100%)	6181	5358	7210

Gene cluster refers to group of genes clustered based on existence in the strains of different lineages. Code for number of genes in lineage BD-0, BD-1, and BD-2 is N_BD-0, N_BD-1, and N_BD-2, respectively.

Table S5A. Genes detected in more than 95% of BD-0, BD-1, and BD-2 strains

Gene	Freq_BD-0	Freq_BD-1	Freq_BD-2	Annotation
hisA	1.00	0.99	1.00	1-(5-phosphoribosyl)-5-[(5-phosphoribosylamino)methylideneamino] imidazole-4-carboxamide isomerase
dhaT	1.00	1.00	1.00	1,3-propanediol dehydrogenase
groS_1	1.00	1.00	1.00	10 kDa chaperonin
groS_2	1.00	0.99	1.00	10 kDa chaperonin
dapD	1.00	1.00	1.00	2,3,4,5-tetrahydropyridine-2,6-dicarboxylate N-succinyltransferase
dhbE	1.00	0.99	1.00	2,3-dihydroxybenzoate-AMP ligase
fadH	1.00	0.99	1.00	2,4-dienoyl-CoA reductase [NADPH]
rlmB_2	1.00	0.99	1.00	23S rRNA (guanosine-2'-O-)-methyltransferase RlmB
rlmC	1.00	1.00	1.00	23S rRNA (uracil(747)-C(5))-methyltransferase RlmC
kbl	1.00	1.00	1.00	2-amino-3-ketobutyrate coenzyme A ligase
ispF	1.00	1.00	1.00	2-C-methyl-D-erythritol 2,4-cyclodiphosphate synthase
ispD_2	1.00	1.00	1.00	2-C-methyl-D-erythritol 4-phosphate cytidylyltransferase
ispD_1	1.00	0.97	1.00	2-C-methyl-D-erythritol 4-phosphate cytidylyltransferase
panE	1.00	1.00	1.00	2-dehydropantoate 2-reductase
thiH	1.00	1.00	1.00	2-iminoacetate synthase

menH	1.00	0.99	1.00	2-succinyl-6-hydroxy-2,4-cyclohexadiene-1-carboxylate synthase
group_3188	1.00	1.00	1.00	3 beta-hydroxysteroid dehydrogenase/Delta 5-->4-isomerase
mhpA	1.00	1.00	1.00	3-(3-hydroxy-phenyl)propionate/3-hydroxycinnamic acid hydroxylase
cpdA	1.00	0.99	1.00	3',5'-cyclic adenosine monophosphate phosphodiesterase CpdA
rpsJ	1.00	1.00	1.00	30S ribosomal protein S10
rpsK	1.00	1.00	1.00	30S ribosomal protein S11
rpsL	1.00	1.00	1.00	30S ribosomal protein S12
rpsM	1.00	0.99	1.00	30S ribosomal protein S13
rpsN	1.00	1.00	1.00	30S ribosomal protein S14
rpsO	1.00	1.00	1.00	30S ribosomal protein S15
rpsP	1.00	1.00	1.00	30S ribosomal protein S16
rpsS	1.00	1.00	1.00	30S ribosomal protein S19
rpsT	1.00	1.00	1.00	30S ribosomal protein S20
rpsU	1.00	0.99	1.00	30S ribosomal protein S21
rpsC	1.00	0.99	1.00	30S ribosomal protein S3
rpsD	1.00	1.00	1.00	30S ribosomal protein S4
rpsE	1.00	1.00	1.00	30S ribosomal protein S5
rpsF	1.00	1.00	1.00	30S ribosomal protein S6
rpsI	1.00	1.00	1.00	30S ribosomal protein S9
aroQ	1.00	0.99	1.00	3-dehydroquinate dehydratase
aroB	1.00	0.99	1.00	3-dehydroquinate synthase
kdsC	1.00	1.00	0.99	3-deoxy-D-manno-octulosonate 8-phosphate phosphatase KdsC
waaA	1.00	1.00	1.00	3-deoxy-D-manno-octulosonic acid transferase
fadA	1.00	0.99	1.00	3-ketoacyl-CoA thiolase
sgbH	1.00	0.97	0.98	3-keto-L-gulonate-6-phosphate decarboxylase SgbH
ubiD	1.00	0.99	1.00	3-octaprenyl-4-hydroxybenzoate carboxy-lyase
fabG_3	1.00	1.00	1.00	3-oxoacyl-[acyl-carrier-protein] reductase FabG
fabG_2	1.00	1.00	1.00	3-oxoacyl-[acyl-carrier-protein] reductase FabG
fabB	1.00	1.00	1.00	3-oxoacyl-[acyl-carrier-protein] synthase 1
fabF	1.00	1.00	1.00	3-oxoacyl-[acyl-carrier-protein] synthase 2

fabH1	1.00	1.00	1.00	3-oxoacyl-[acyl-carrier-protein] synthase 3 protein 1
malQ	1.00	0.99	0.99	4-alpha-glucanotransferase
ispG	1.00	1.00	1.00	4-hydroxy-3-methylbut-2-en-1-yl diphosphate synthase
ispH	1.00	1.00	1.00	4-hydroxy-3-methylbut-2-enyl diphosphate reductase
rplK	1.00	1.00	1.00	50S ribosomal protein L11
rplM	1.00	1.00	1.00	50S ribosomal protein L13
rplS	1.00	1.00	1.00	50S ribosomal protein L19
rplB	1.00	1.00	1.00	50S ribosomal protein L2
rplV	1.00	1.00	1.00	50S ribosomal protein L22
rplW	1.00	0.99	1.00	50S ribosomal protein L23
rplX	1.00	1.00	1.00	50S ribosomal protein L24
rpmA	1.00	1.00	1.00	50S ribosomal protein L27
rpmB	1.00	1.00	1.00	50S ribosomal protein L28
rpmC	1.00	1.00	1.00	50S ribosomal protein L29
rplC	1.00	1.00	1.00	50S ribosomal protein L3
rpmD	1.00	0.99	1.00	50S ribosomal protein L30
rpmE	1.00	1.00	1.00	50S ribosomal protein L31
rpmF	1.00	1.00	1.00	50S ribosomal protein L32
rpmG	1.00	0.99	0.99	50S ribosomal protein L33
rpmH	1.00	0.96	1.00	50S ribosomal protein L34
rpmI	1.00	1.00	1.00	50S ribosomal protein L35
rpmJ	1.00	1.00	1.00	50S ribosomal protein L36
rplD	1.00	1.00	1.00	50S ribosomal protein L4
rplF	1.00	1.00	1.00	50S ribosomal protein L6
rplI	1.00	1.00	1.00	50S ribosomal protein L9
mtnN	1.00	1.00	1.00	5'-methylthioadenosine/S-adenosylhomocysteine nucleosidase
ribE_2	1.00	1.00	1.00	6,7-dimethyl-8-ribityllumazine synthase
groL_2	1.00	0.99	1.00	60 kDa chaperonin
groL_1	1.00	0.99	0.99	60 kDa chaperonin
yieH	1.00	1.00	1.00	6-phosphogluconate phosphatase
pgl	1.00	1.00	1.00	6-phosphogluconolactonase
queC	1.00	1.00	0.99	7-cyano-7-deazaguanine synthase

artI_2	1.00	1.00	1.00	ABC transporter arginine-binding protein precursor
ilvM	1.00	0.99	1.00	Acetolactate synthase isozyme 2 small subunit
ilvI	1.00	0.99	1.00	Acetolactate synthase isozyme 3 large subunit
ilvH	1.00	1.00	1.00	Acetolactate synthase isozyme 3 small subunit
accA	1.00	0.99	1.00	Acetyl-coenzyme A carboxylase carboxyl transferase subunit alpha
argB	1.00	1.00	1.00	Acetylglutamate kinase
ypeA_2	1.00	0.99	1.00	Acetyltransferase YpeA
ypeA_1	1.00	0.99	1.00	Acetyltransferase YpeA
tesB	1.00	1.00	1.00	Acyl-CoA thioesterase 2
fadE_2	1.00	1.00	1.00	Acyl-coenzyme A dehydrogenase
cyaA	1.00	1.00	1.00	Adenylate cyclase
purA	1.00	1.00	1.00	Adenylosuccinate synthetase
rfaF_1	1.00	1.00	1.00	ADP-heptose--LPS heptosyltransferase 2
rfaF_2	1.00	0.99	1.00	ADP-heptose--LPS heptosyltransferase 2
nudF_1	1.00	1.00	1.00	ADP-ribose pyrophosphatase
glpD	1.00	0.99	0.99	Aerobic glycerol-3-phosphate dehydrogenase
aer_2	1.00	1.00	1.00	Aerotaxis receptor
tqsA	1.00	1.00	1.00	AI-2 transport protein TqsA
alaS_1	1.00	1.00	1.00	Alanine--tRNA ligase
ssuB	1.00	1.00	1.00	Aliphatic sulfonates import ATP-binding protein SsuB
phoP	1.00	1.00	1.00	Alkaline phosphatase synthesis transcriptional regulatory protein PhoP
lysX	1.00	1.00	1.00	Alpha-aminoadipate--LysW ligase LysX
rafA	1.00	0.99	1.00	Alpha-galactosidase
hlyB	1.00	0.99	0.99	Alpha-hemolysin translocation ATP-binding protein HlyB
purF	1.00	0.97	1.00	Amidophosphoribosyltransferase
argA	1.00	1.00	1.00	Amino-acid acetyltransferase
alsT_1	1.00	1.00	1.00	Amino-acid carrier protein AlsT
gcvT	1.00	0.99	1.00	Aminomethyltransferase
amtB	1.00	1.00	1.00	Ammonia channel precursor
anmK	1.00	0.99	1.00	Anhydro-N-acetylmuramic acid kinase
chrR	1.00	0.99	1.00	Anti-sigma-E factor ChrR

rseA	1.00	0.97	1.00	Anti-sigma-E factor RseA
cptB	1.00	0.99	1.00	Antitoxin CptB
citX	1.00	1.00	1.00	Apo-citrate lyase phosphoribosyl-dephospho-CoA transferase
kdsD	1.00	0.99	0.98	Arabinose 5-phosphate isomerase KdsD
artM	1.00	0.99	1.00	Arginine ABC transporter permease protein ArtM
artQ	1.00	1.00	1.00	Arginine ABC transporter permease protein ArtQ
argO	1.00	1.00	1.00	Arginine exporter protein ArgO
artP	1.00	1.00	1.00	Arginine transport ATP-binding protein ArtP
tyrB	1.00	1.00	1.00	Aromatic-amino-acid aminotransferase
ulaC	1.00	1.00	1.00	Ascorbate-specific phosphotransferase enzyme IIA component
asnB	1.00	1.00	1.00	Asparagine synthetase B [glutamine-hydrolyzing]
pyrB	1.00	1.00	1.00	Aspartate carbamoyltransferase catalytic chain
pyrI	1.00	1.00	1.00	Aspartate carbamoyltransferase regulatory chain
lysC_2	1.00	1.00	1.00	Aspartokinase
hisG	1.00	1.00	1.00	ATP phosphoribosyltransferase
atpC	1.00	0.99	0.99	ATP synthase epsilon chain
atpG	1.00	1.00	1.00	ATP synthase gamma chain
atpB	1.00	1.00	1.00	ATP synthase subunit a
atpA	1.00	0.99	1.00	ATP synthase subunit alpha
atpF	1.00	1.00	1.00	ATP synthase subunit b
atpE	1.00	1.00	1.00	ATP synthase subunit c
atpH	1.00	1.00	1.00	ATP synthase subunit delta
clpP	1.00	1.00	1.00	ATP-dependent Clp protease proteolytic subunit precursor
bioD1	1.00	1.00	1.00	ATP-dependent dethiobiotin synthetase BioD 1
recQ	1.00	0.97	1.00	ATP-dependent DNA helicase RecQ
hslU	1.00	0.99	1.00	ATP-dependent protease ATPase subunit HslU
hslV	1.00	0.99	1.00	ATP-dependent protease subunit HslV
rhlB	1.00	1.00	0.99	ATP-dependent RNA helicase RhlB
rhlE_3	1.00	1.00	1.00	ATP-dependent RNA helicase RhlE
luxQ_3	1.00	1.00	1.00	Autoinducer 2 sensor kinase/phosphatase LuxQ
grcA	1.00	1.00	1.00	Autonomous glycyl radical cofactor
bfd	1.00	1.00	0.99	Bacterioferritin-associated ferredoxin

bicA_1	1.00	1.00	1.00	Bicarbonate transporter BicA
bicA_2	1.00	1.00	1.00	Bicarbonate transporter BicA
thrA_2	1.00	1.00	1.00	Bifunctional aspartokinase/homoserine dehydrogenase 1
thrA_1	1.00	1.00	1.00	Bifunctional aspartokinase/homoserine dehydrogenase 1
folC	1.00	1.00	1.00	Bifunctional protein FolC
folD	1.00	1.00	1.00	Bifunctional protein FolD protein
glmU	1.00	0.99	1.00	Bifunctional protein GlmU
putA	1.00	0.99	1.00	Bifunctional protein PutA
purH	1.00	1.00	1.00	Bifunctional purine biosynthesis protein PurH
bdlA_2	1.00	1.00	1.00	Biofilm dispersion protein BdlA
accB	1.00	1.00	1.00	Biotin carboxyl carrier protein of acetyl-CoA carboxylase
accC	1.00	0.99	0.99	Biotin carboxylase
brnQ	1.00	0.99	1.00	Branched-chain amino acid transport system 2 carrier protein
ilvE	1.00	0.99	1.00	Branched-chain-amino-acid aminotransferase
dctB_3	1.00	1.00	1.00	C4-dicarboxylate transport sensor protein DctB
dctB_1	1.00	1.00	1.00	C4-dicarboxylate transport sensor protein DctB
dctD_1	1.00	0.99	1.00	C4-dicarboxylate transport transcriptional regulatory protein DctD
crp	1.00	1.00	1.00	cAMP-activated global transcriptional regulator CRP
carA	1.00	0.99	1.00	Carbamoyl-phosphate synthase small chain
caiE	1.00	1.00	1.00	Carnitine operon protein CaiE
katG	1.00	1.00	1.00	Catalase-peroxidase
fiu	1.00	0.99	1.00	Catecholate siderophore receptor Fiu precursor
ftsE	1.00	0.99	1.00	Cell division ATP-binding protein FtsE
ftsB	1.00	1.00	1.00	Cell division protein FtsB
ftsN	1.00	0.99	1.00	Cell division protein FtsN
ftsX	1.00	1.00	1.00	Cell division protein FtsX
ftsZ	1.00	0.99	1.00	Cell division protein FtsZ
zipA	1.00	1.00	1.00	Cell division protein ZipA
mreC	1.00	1.00	1.00	Cell shape-determining protein MreC
htpG	1.00	1.00	1.00	Chaperone protein HtpG

skp	1.00	0.99	1.00	Chaperone protein Skp precursor
yajL	1.00	0.99	1.00	Chaperone protein YajL
cheA_2	1.00	1.00	1.00	Chemotaxis protein CheA
cheA_3	1.00	1.00	1.00	Chemotaxis protein CheA
cheV_3	1.00	1.00	1.00	Chemotaxis protein CheV
group_3379	1.00	1.00	1.00	Chemotaxis protein CheV
cheV_2	1.00	1.00	1.00	Chemotaxis protein CheV
cheV_1	1.00	1.00	1.00	Chemotaxis protein CheV
cheW_4	1.00	1.00	1.00	Chemotaxis protein CheW
cheW_1	1.00	1.00	1.00	Chemotaxis protein CheW
cheW_2	1.00	0.99	1.00	Chemotaxis protein CheW
group_5564	1.00	0.99	1.00	Chemotaxis protein CheW
cheX	1.00	1.00	1.00	CheY-P phosphatase CheX
cbh	1.00	0.99	1.00	Choloylglycine hydrolase
aroC	1.00	0.99	1.00	Chorismate synthase
iciA	1.00	0.99	1.00	Chromosome initiation inhibitor
citF	1.00	0.99	1.00	Citrate lyase alpha chain
btuR	1.00	1.00	1.00	Cob(I)yrinic acid a,c-diamide adenosyltransferase
cbiB	1.00	1.00	1.00	Cobalamin biosynthesis protein CbiB
czcB	1.00	1.00	1.00	Cobalt-zinc-cadmium resistance protein CzcB
cobQ	1.00	0.99	1.00	Cobyric acid synthase
coaBC	1.00	1.00	1.00	Coenzyme A biosynthesis bifunctional protein CoaBC
cvpA	1.00	1.00	1.00	Colicin V production protein
calB	1.00	1.00	1.00	Coniferyl aldehyde dehydrogenase
hemF	1.00	0.99	1.00	Coproporphyrinogen-III oxidase, aerobic
nudG	1.00	1.00	1.00	CTP pyrophosphohydrolase
pyrG	1.00	1.00	1.00	CTP synthase
rpfG_6	1.00	1.00	1.00	Cyclic di-GMP phosphodiesterase response regulator RpfG
rpfG_1	1.00	1.00	1.00	Cyclic di-GMP phosphodiesterase response regulator RpfG
rpfG_7	1.00	1.00	1.00	Cyclic di-GMP phosphodiesterase response regulator RpfG

rpfG_4	1.00	0.99	1.00	Cyclic di-GMP phosphodiesterase response regulator RpfG
moaA	1.00	1.00	1.00	Cyclic pyranopterin monophosphate synthase
moaC	1.00	0.99	1.00	Cyclic pyranopterin monophosphate synthase accessory protein
iscS	1.00	0.97	1.00	Cysteine desulfurase
cysK	1.00	0.99	1.00	Cysteine synthase A
fliY	1.00	1.00	1.00	Cystine-binding periplasmic protein precursor
fbcH	1.00	1.00	1.00	Cytochrome b/c1
cydB_1	1.00	1.00	1.00	Cytochrome bd-I ubiquinol oxidase subunit 2
cydX	1.00	1.00	1.00	Cytochrome bd-I ubiquinol oxidase subunit X
cc4	1.00	1.00	1.00	Cytochrome c4 precursor
ccmH_1	1.00	1.00	1.00	Cytochrome c-type biogenesis protein CcmH precursor
napC	1.00	0.99	1.00	Cytochrome c-type protein NapC
torY	1.00	1.00	1.00	Cytochrome c-type protein TorY
rodZ	1.00	1.00	1.00	Cytoskeleton protein RodZ
pepD	1.00	1.00	0.99	Cytosol non-specific dipeptidase
dgt_2	1.00	1.00	1.00	Deoxyguanosinetriphosphate triphosphohydrolase
deoR	1.00	0.99	1.00	Deoxyribonucleoside regulator
coaE	1.00	0.97	1.00	Dephospho-CoA kinase
yihI	1.00	1.00	1.00	Der GTPase-activating protein YihI
epd	1.00	1.00	1.00	D-erythrose-4-phosphate dehydrogenase
dapF	1.00	0.99	1.00	Diaminopimelate epimerase
dosC_1	1.00	1.00	1.00	Diguanylate cyclase DosC
dosC_2	1.00	1.00	1.00	Diguanylate cyclase DosC
lpd	1.00	1.00	1.00	Dihydrolipoyl dehydrogenase
mshA	1.00	1.00	1.00	D-inositol 3-phosphate glycosyltransferase
dppC_1	1.00	1.00	1.00	Dipeptide transport system permease protein DppC
dppC_2	1.00	0.99	1.00	Dipeptide transport system permease protein DppC
rdgB	1.00	1.00	1.00	dITP/XTP pyrophosphatase
dam	1.00	0.99	1.00	DNA adenine methylase
ligA_2	1.00	0.97	1.00	DNA ligase

mutL_1	1.00	1.00	1.00	DNA mismatch repair protein MutL
dnaN	1.00	0.99	1.00	DNA polymerase III subunit beta
holC	1.00	1.00	1.00	DNA polymerase III subunit chi
holB	1.00	1.00	1.00	DNA polymerase III subunit delta'
dnaQ	1.00	1.00	1.00	DNA polymerase III subunit epsilon
dinB_3	1.00	1.00	1.00	DNA polymerase IV
dps	1.00	1.00	1.00	DNA protection during starvation protein
rmuC	1.00	1.00	1.00	DNA recombination protein RmuC
recN	1.00	1.00	1.00	DNA repair protein RecN
recO	1.00	0.97	1.00	DNA repair protein RecO
recF_1	1.00	0.99	1.00	DNA replication and repair protein RecF
parC	1.00	1.00	1.00	DNA topoisomerase 4 subunit A
ftsK	1.00	0.99	0.98	DNA translocase FtsK
diaA	1.00	1.00	1.00	DnaA initiator-associating protein DiaA
hupB	1.00	1.00	1.00	DNA-binding protein HU-beta
rbsB	1.00	1.00	1.00	D-ribose-binding periplasmic protein precursor
dtd	1.00	0.97	1.00	D-tyrosyl-tRNA(Tyr) deacylase
rlmN	1.00	1.00	1.00	Dual-specificity RNA methyltransferase RlmN
rpoE_1	1.00	1.00	1.00	ECF RNA polymerase sigma-E factor
rnfA	1.00	1.00	1.00	Electron transport complex protein RnfA
rnfD	1.00	1.00	1.00	Electron transport complex protein RnfD
lepA	1.00	1.00	1.00	Elongation factor 4
fusA_1	1.00	0.99	1.00	Elongation factor G
efp	1.00	0.99	1.00	Elongation factor P
epmA	1.00	1.00	1.00	Elongation factor P--(R)-beta-lysine ligase
yeiP	1.00	1.00	1.00	Elongation factor P-like protein
tsf	1.00	1.00	1.00	Elongation factor Ts
ridA	1.00	0.99	1.00	Enamine/imine deaminase
nfo	1.00	1.00	1.00	Endonuclease 4
nth	1.00	1.00	1.00	Endonuclease III
queG	1.00	0.99	1.00	Epoxyqueuosine reductase
pdxB	1.00	1.00	1.00	Erythronate-4-phosphate dehydrogenase
ybfF	1.00	1.00	1.00	Esterase YbfF
xseA	1.00	1.00	1.00	Exodeoxyribonuclease 7 large subunit

sbcB	1.00	1.00	1.00	Exodeoxyribonuclease I
dns	1.00	1.00	1.00	Extracellular deoxyribonuclease precursor
pstI	1.00	1.00	1.00	Extracellular serine proteinase precursor
fecA	1.00	1.00	0.99	Fe(3+) dicitrate transport protein FecA precursor
fbpC	1.00	0.99	0.99	Fe(3+) ions import ATP-binding protein FbpC
nfuA	1.00	1.00	1.00	Fe/S biogenesis protein NfuA
group_4171	1.00	1.00	1.00	Ferredoxin 1
group_1966	1.00	1.00	0.99	Ferredoxin--NADP reductase
fhuA_1	1.00	1.00	1.00	Ferrichrome-iron receptor precursor
fhuA_2	1.00	0.99	1.00	Ferrichrome-iron receptor precursor
fepB	1.00	1.00	1.00	Ferrienterobactin-binding periplasmic protein precursor
hemH	1.00	1.00	1.00	Ferrochelatase
pilE	1.00	1.00	1.00	Fimbrial protein precursor
fkpB	1.00	1.00	1.00	FKBP-type 16 kDa peptidyl-prolyl cis-trans isomerase
fkpA_2	1.00	0.99	1.00	FKBP-type peptidyl-prolyl cis-trans isomerase FkpA precursor
flgC	1.00	1.00	1.00	Flagellar basal-body rod protein FlgC
flgF	1.00	1.00	1.00	Flagellar basal-body rod protein FlgF
flhA	1.00	1.00	1.00	Flagellar biosynthesis protein FlhA
fliP	1.00	1.00	1.00	Flagellar biosynthetic protein FliP precursor
fliQ	1.00	1.00	1.00	Flagellar biosynthetic protein FliQ
flgE	1.00	1.00	1.00	Flagellar hook protein FlgE
flgK	1.00	1.00	1.00	Flagellar hook-associated protein 1
fliD	1.00	0.97	1.00	Flagellar hook-associated protein 2
flgL	1.00	1.00	1.00	Flagellar hook-associated protein 3
fliE	1.00	1.00	1.00	Flagellar hook-basal body complex protein FliE
fliK	1.00	0.99	1.00	Flagellar hook-length control protein
fliG	1.00	0.99	1.00	Flagellar motor switch protein FliG
flgI	1.00	1.00	1.00	Flagellar P-ring protein precursor
flaC	1.00	1.00	1.00	Flagellin C
hmp	1.00	1.00	1.00	Flavohemoprotein
fdoI	1.00	1.00	1.00	Formate dehydrogenase, cytochrome b556(fdo) subunit
hutG_1	1.00	0.97	1.00	Formimidoylglutamase

purU	1.00	1.00	1.00	Formyltetrahydrofolate deformylase
glpX	1.00	0.99	1.00	Fructose-1,6-bisphosphatase 1 class 2
yqaB	1.00	0.99	1.00	Fructose-1-phosphate phosphatase YqaB
fbaA	1.00	1.00	1.00	Fructose-bisphosphate aldolase class 2
frdA	1.00	1.00	1.00	Fumarate reductase flavoprotein subunit
frdB	1.00	1.00	1.00	Fumarate reductase iron-sulfur subunit
frdC	1.00	1.00	1.00	Fumarate reductase subunit C
nagK_2	1.00	1.00	1.00	Fumarylpyruvate hydrolase
galT	1.00	1.00	1.00	Galactose-1-phosphate uridylyltransferase
mglC	1.00	0.99	1.00	Galactoside transport system permease protein MglC
gbpA_2	1.00	1.00	1.00	GlcNAc-binding protein A precursor
gspK_2	1.00	1.00	1.00	Glucosamine kinase GspK
nagB	1.00	1.00	1.00	Glucosamine-6-phosphate deaminase
crr	1.00	1.00	1.00	Glucose-specific phosphotransferase enzyme IIA component
gcdB	1.00	1.00	1.00	Glutaconyl-CoA decarboxylase subunit beta
proB	1.00	0.99	1.00	Glutamate 5-kinase
gltX	1.00	1.00	1.00	Glutamate--tRNA ligase
glsA2	1.00	0.99	1.00	Glutaminase 2
glnA	1.00	1.00	1.00	Glutamine synthetase
glnQ_1	1.00	0.99	1.00	Glutamine transport ATP-binding protein GlnQ
glnQ_2	1.00	0.99	1.00	Glutamine transport ATP-binding protein GlnQ
glnS	1.00	0.99	1.00	Glutamine--tRNA ligase
gluQ	1.00	1.00	1.00	Glutamyl-Q tRNA(Asp) synthetase
grxB	1.00	1.00	1.00	Glutaredoxin-2
gsiA_5	1.00	1.00	1.00	Glutathione import ATP-binding protein GsiA
gsiA_4	1.00	1.00	1.00	Glutathione import ATP-binding protein GsiA
gsiA_1	1.00	1.00	1.00	Glutathione import ATP-binding protein GsiA
fosA	1.00	1.00	1.00	Glutathione transferase FosA
gap	1.00	0.99	0.99	Glyceraldehyde-3-phosphate dehydrogenase
glpQ	1.00	1.00	1.00	Glycerophosphoryl diester phosphodiesterase precursor
gcvA_1	1.00	1.00	1.00	Glycine cleavage system transcriptional activator
gcvA_4	1.00	0.99	1.00	Glycine cleavage system transcriptional activator

gcvR	1.00	1.00	1.00	Glycine cleavage system transcriptional repressor
glyQ	1.00	0.99	1.00	Glycine--tRNA ligase alpha subunit
glyS	1.00	0.99	1.00	Glycine--tRNA ligase beta subunit
gtf1	1.00	1.00	1.00	Glycogen synthase
glgA	1.00	1.00	0.98	Glycogen synthase
ghrA	1.00	1.00	1.00	Glyoxylate/hydroxypyruvate reductase A
guaC	1.00	1.00	1.00	GMP reductase
folE	1.00	1.00	1.00	GTP cyclohydrolase 1
gmk	1.00	1.00	1.00	Guanylate kinase
hslR	1.00	0.99	1.00	Heat shock protein 15
ccmC	1.00	1.00	1.00	Heme exporter protein C
hmuV	1.00	1.00	1.00	Hemin import ATP-binding protein HmuV
hmuT	1.00	1.00	1.00	Hemin-binding periplasmic protein HmuT precursor
tlyC	1.00	1.00	1.00	Hemolysin C
hlyD	1.00	1.00	1.00	Hemolysin secretion protein D, chromosomal
znuA	1.00	1.00	0.99	High-affinity zinc uptake system protein ZnuA precursor
hutH	1.00	0.99	1.00	Histidine ammonia-lyase
hisS	1.00	1.00	1.00	Histidine--tRNA ligase
cidA_1	1.00	0.99	1.00	Holin-like protein CidA
thrB_1	1.00	1.00	1.00	Homoserine kinase
thrB_2	1.00	0.99	1.00	Homoserine kinase
rhtB_1	1.00	0.99	1.00	Homoserine/homoserine lactone efflux protein
btr_2	1.00	1.00	1.00	HTH-type transcriptional activator Btr
rhaR	1.00	1.00	1.00	HTH-type transcriptional activator RhaR
rhaS	1.00	1.00	1.00	HTH-type transcriptional activator RhaS
benM	1.00	1.00	1.00	HTH-type transcriptional regulator BenM
betI	1.00	1.00	1.00	HTH-type transcriptional regulator BetI
cynR_2	1.00	1.00	1.00	HTH-type transcriptional regulator CynR
dmlR_7	1.00	1.00	1.00	HTH-type transcriptional regulator DmlR
dmlR_1	1.00	1.00	1.00	HTH-type transcriptional regulator DmlR
dmlR_2	1.00	1.00	1.00	HTH-type transcriptional regulator DmlR
dmlR_10	1.00	1.00	1.00	HTH-type transcriptional regulator DmlR
dmlR_6	1.00	1.00	1.00	HTH-type transcriptional regulator DmlR

group_3356	1.00	1.00	1.00	HTH-type transcriptional regulator DmlR
dmlR_5	1.00	1.00	1.00	HTH-type transcriptional regulator DmlR
galR_2	1.00	0.99	1.00	HTH-type transcriptional regulator GalR
gltR_2	1.00	1.00	1.00	HTH-type transcriptional regulator GltR
gntR	1.00	0.96	0.98	HTH-type transcriptional regulator GntR
iscR	1.00	1.00	1.00	HTH-type transcriptional regulator IscR
leuO_1	1.00	1.00	1.00	HTH-type transcriptional regulator LeuO
leuO_2	1.00	0.99	1.00	HTH-type transcriptional regulator LeuO
puuR	1.00	1.00	1.00	HTH-type transcriptional regulator PuuR
ttgR	1.00	0.97	1.00	HTH-type transcriptional regulator TtgR
ulaR	1.00	1.00	1.00	HTH-type transcriptional regulator UlaR
yidZ	1.00	0.99	1.00	HTH-type transcriptional regulator YidZ
comR_1	1.00	1.00	1.00	HTH-type transcriptional repressor ComR
comR_2	1.00	1.00	0.99	HTH-type transcriptional repressor ComR
cytR	1.00	0.99	1.00	HTH-type transcriptional repressor CytR
fabR	1.00	1.00	1.00	HTH-type transcriptional repressor FabR
oxyR_2	1.00	1.00	1.00	Hydrogen peroxide-inducible genes activator
hupR1_1	1.00	1.00	1.00	Hydrogenase transcriptional regulatory protein hupR1
gloB	1.00	1.00	1.00	Hydroxyacylglutathione hydrolase
group_10036	1.00	1.00	1.00	hypothetical protein
group_10044	1.00	1.00	1.00	hypothetical protein
group_1489	1.00	1.00	1.00	hypothetical protein
group_1565	1.00	1.00	1.00	hypothetical protein
group_1588	1.00	1.00	1.00	hypothetical protein
group_1704	1.00	1.00	1.00	hypothetical protein
group_1817	1.00	1.00	1.00	hypothetical protein
group_1905	1.00	1.00	1.00	hypothetical protein
group_1937	1.00	1.00	1.00	hypothetical protein
group_1991	1.00	1.00	1.00	hypothetical protein
group_2076	1.00	1.00	1.00	hypothetical protein
group_2108	1.00	1.00	1.00	hypothetical protein
group_2122	1.00	1.00	1.00	hypothetical protein
group_2142	1.00	1.00	1.00	hypothetical protein
group_2154	1.00	1.00	1.00	hypothetical protein

group_2183	1.00	1.00	1.00	hypothetical protein
group_2211	1.00	1.00	1.00	hypothetical protein
group_2222	1.00	1.00	1.00	hypothetical protein
group_2472	1.00	1.00	1.00	hypothetical protein
group_2565	1.00	1.00	1.00	hypothetical protein
group_2582	1.00	1.00	1.00	hypothetical protein
group_2593	1.00	1.00	1.00	hypothetical protein
group_2608	1.00	1.00	1.00	hypothetical protein
group_2618	1.00	1.00	1.00	hypothetical protein
group_2698	1.00	1.00	1.00	hypothetical protein
group_2767	1.00	1.00	1.00	hypothetical protein
group_2777	1.00	1.00	1.00	hypothetical protein
group_2854	1.00	1.00	1.00	hypothetical protein
group_2915	1.00	1.00	1.00	hypothetical protein
group_3079	1.00	1.00	1.00	hypothetical protein
group_3090	1.00	1.00	1.00	hypothetical protein
group_3093	1.00	1.00	1.00	hypothetical protein
group_3106	1.00	1.00	1.00	hypothetical protein
group_3163	1.00	1.00	1.00	hypothetical protein
group_3168	1.00	1.00	1.00	hypothetical protein
group_3257	1.00	1.00	1.00	hypothetical protein
group_3272	1.00	1.00	1.00	hypothetical protein
group_3277	1.00	1.00	1.00	hypothetical protein
group_3297	1.00	1.00	1.00	hypothetical protein
group_3313	1.00	1.00	1.00	hypothetical protein
group_3314	1.00	1.00	1.00	hypothetical protein
group_3323	1.00	1.00	1.00	hypothetical protein
group_3345	1.00	1.00	1.00	hypothetical protein
group_3362	1.00	1.00	1.00	hypothetical protein
group_3372	1.00	1.00	1.00	hypothetical protein
group_3392	1.00	1.00	1.00	hypothetical protein
group_3457	1.00	1.00	1.00	hypothetical protein
group_3470	1.00	1.00	1.00	hypothetical protein
group_3526	1.00	1.00	1.00	hypothetical protein

group_3529	1.00	1.00	1.00	hypothetical protein
group_3550	1.00	1.00	1.00	hypothetical protein
group_3675	1.00	1.00	1.00	hypothetical protein
group_3718	1.00	1.00	1.00	hypothetical protein
group_3724	1.00	1.00	1.00	hypothetical protein
group_3744	1.00	1.00	1.00	hypothetical protein
group_3775	1.00	1.00	1.00	hypothetical protein
group_3788	1.00	1.00	1.00	hypothetical protein
group_3793	1.00	1.00	1.00	hypothetical protein
group_3799	1.00	1.00	1.00	hypothetical protein
group_3800	1.00	1.00	1.00	hypothetical protein
group_3810	1.00	1.00	1.00	hypothetical protein
group_3820	1.00	1.00	1.00	hypothetical protein
group_3834	1.00	1.00	1.00	hypothetical protein
group_3842	1.00	1.00	1.00	hypothetical protein
group_3860	1.00	1.00	1.00	hypothetical protein
ttgW	1.00	1.00	1.00	hypothetical protein
group_3902	1.00	1.00	1.00	hypothetical protein
group_3907	1.00	1.00	1.00	hypothetical protein
group_3911	1.00	1.00	1.00	hypothetical protein
group_3931	1.00	1.00	1.00	hypothetical protein
group_3946	1.00	1.00	1.00	hypothetical protein
group_3954	1.00	1.00	1.00	hypothetical protein
group_3963	1.00	1.00	1.00	hypothetical protein
group_3964	1.00	1.00	1.00	hypothetical protein
group_3973	1.00	1.00	1.00	hypothetical protein
group_3981	1.00	1.00	1.00	hypothetical protein
group_3992	1.00	1.00	1.00	hypothetical protein
group_3998	1.00	1.00	1.00	hypothetical protein
group_4024	1.00	1.00	1.00	hypothetical protein
group_4034	1.00	1.00	1.00	hypothetical protein
group_4035	1.00	1.00	1.00	hypothetical protein
group_4041	1.00	1.00	1.00	hypothetical protein
group_4069	1.00	1.00	1.00	hypothetical protein

group_4087	1.00	1.00	1.00	hypothetical protein
group_4092	1.00	1.00	1.00	hypothetical protein
group_4104	1.00	1.00	1.00	hypothetical protein
group_4105	1.00	1.00	1.00	hypothetical protein
group_4124	1.00	1.00	1.00	hypothetical protein
group_4130	1.00	1.00	1.00	hypothetical protein
group_4138	1.00	1.00	1.00	hypothetical protein
group_4139	1.00	1.00	1.00	hypothetical protein
group_4149	1.00	1.00	1.00	hypothetical protein
group_4165	1.00	1.00	1.00	hypothetical protein
group_4170	1.00	1.00	1.00	hypothetical protein
group_4180	1.00	1.00	1.00	hypothetical protein
group_4181	1.00	1.00	1.00	hypothetical protein
group_4205	1.00	1.00	1.00	hypothetical protein
group_4207	1.00	1.00	1.00	hypothetical protein
group_4212	1.00	1.00	1.00	hypothetical protein
group_4223	1.00	1.00	1.00	hypothetical protein
group_4420	1.00	1.00	1.00	hypothetical protein
group_4453	1.00	1.00	1.00	hypothetical protein
group_4458	1.00	1.00	1.00	hypothetical protein
group_4467	1.00	1.00	1.00	hypothetical protein
group_4527	1.00	1.00	1.00	hypothetical protein
group_4536	1.00	1.00	1.00	hypothetical protein
group_4547	1.00	1.00	1.00	hypothetical protein
group_4555	1.00	1.00	1.00	hypothetical protein
group_4575	1.00	1.00	1.00	hypothetical protein
group_4576	1.00	1.00	1.00	hypothetical protein
group_4577	1.00	1.00	1.00	hypothetical protein
group_4586	1.00	1.00	1.00	hypothetical protein
group_4589	1.00	1.00	1.00	hypothetical protein
group_4598	1.00	1.00	1.00	hypothetical protein
group_4633	1.00	1.00	1.00	hypothetical protein
group_4634	1.00	1.00	1.00	hypothetical protein
group_4644	1.00	1.00	1.00	hypothetical protein

group_4665	1.00	1.00	1.00	hypothetical protein
group_4713	1.00	1.00	1.00	hypothetical protein
group_4716	1.00	1.00	1.00	hypothetical protein
group_4737	1.00	1.00	1.00	hypothetical protein
group_4738	1.00	1.00	1.00	hypothetical protein
group_4739	1.00	1.00	1.00	hypothetical protein
group_4744	1.00	1.00	1.00	hypothetical protein
group_4747	1.00	1.00	1.00	hypothetical protein
group_4750	1.00	1.00	1.00	hypothetical protein
group_4758	1.00	1.00	1.00	hypothetical protein
group_4772	1.00	1.00	1.00	hypothetical protein
group_4799	1.00	1.00	1.00	hypothetical protein
group_4810	1.00	1.00	1.00	hypothetical protein
group_4861	1.00	1.00	1.00	hypothetical protein
group_4886	1.00	1.00	1.00	hypothetical protein
group_4887	1.00	1.00	1.00	hypothetical protein
group_4889	1.00	1.00	1.00	hypothetical protein
group_4896	1.00	1.00	1.00	hypothetical protein
group_4898	1.00	1.00	1.00	hypothetical protein
group_4899	1.00	1.00	1.00	hypothetical protein
group_5149	1.00	1.00	1.00	hypothetical protein
group_5157	1.00	1.00	1.00	hypothetical protein
group_970	1.00	1.00	1.00	hypothetical protein
group_9866	1.00	1.00	1.00	hypothetical protein
group_9869	1.00	1.00	1.00	hypothetical protein
group_9870	1.00	1.00	1.00	hypothetical protein
group_9872	1.00	1.00	1.00	hypothetical protein
group_9876	1.00	1.00	1.00	hypothetical protein
group_9883	1.00	1.00	1.00	hypothetical protein
group_9884	1.00	1.00	1.00	hypothetical protein
group_9886	1.00	1.00	1.00	hypothetical protein
group_9894	1.00	1.00	1.00	hypothetical protein
group_9895	1.00	1.00	1.00	hypothetical protein
group_9897	1.00	1.00	1.00	hypothetical protein

group_9906	1.00	1.00	1.00	hypothetical protein
group_9908	1.00	1.00	1.00	hypothetical protein
group_9909	1.00	1.00	1.00	hypothetical protein
group_9914	1.00	1.00	1.00	hypothetical protein
group_9915	1.00	1.00	1.00	hypothetical protein
group_9918	1.00	1.00	1.00	hypothetical protein
group_9920	1.00	1.00	1.00	hypothetical protein
group_9924	1.00	1.00	1.00	hypothetical protein
group_9928	1.00	1.00	1.00	hypothetical protein
group_9929	1.00	1.00	1.00	hypothetical protein
group_9933	1.00	1.00	1.00	hypothetical protein
group_9934	1.00	1.00	1.00	hypothetical protein
group_9935	1.00	1.00	1.00	hypothetical protein
group_9941	1.00	1.00	1.00	hypothetical protein
group_9945	1.00	1.00	1.00	hypothetical protein
group_9949	1.00	1.00	1.00	hypothetical protein
group_9953	1.00	1.00	1.00	hypothetical protein
group_9954	1.00	1.00	1.00	hypothetical protein
group_9955	1.00	1.00	1.00	hypothetical protein
group_9957	1.00	1.00	1.00	hypothetical protein
group_9961	1.00	1.00	1.00	hypothetical protein
group_9962	1.00	1.00	1.00	hypothetical protein
group_9964	1.00	1.00	1.00	hypothetical protein
group_1386	1.00	0.99	1.00	hypothetical protein
group_2065	1.00	0.99	1.00	hypothetical protein
group_2757	1.00	0.99	1.00	hypothetical protein
group_2778	1.00	0.99	1.00	hypothetical protein
group_3055	1.00	1.00	0.99	hypothetical protein
group_3119	1.00	0.99	1.00	hypothetical protein
group_3166	1.00	0.99	1.00	hypothetical protein
group_3175	1.00	0.99	1.00	hypothetical protein
group_3176	1.00	0.99	1.00	hypothetical protein
group_3181	1.00	0.99	1.00	hypothetical protein
group_3249	1.00	0.99	1.00	hypothetical protein

group_3258	1.00	0.99	1.00	hypothetical protein
group_3286	1.00	0.99	1.00	hypothetical protein
group_3596	1.00	0.99	1.00	hypothetical protein
group_3703	1.00	0.99	1.00	hypothetical protein
group_3735	1.00	0.99	1.00	hypothetical protein
group_3747	1.00	0.99	1.00	hypothetical protein
group_3774	1.00	0.99	1.00	hypothetical protein
group_3821	1.00	0.99	1.00	hypothetical protein
group_3827	1.00	0.99	1.00	hypothetical protein
group_3849	1.00	0.99	1.00	hypothetical protein
group_3945	1.00	0.99	1.00	hypothetical protein
group_3947	1.00	0.99	1.00	hypothetical protein
group_3975	1.00	1.00	0.99	hypothetical protein
group_4010	1.00	0.99	1.00	hypothetical protein
group_4058	1.00	0.99	1.00	hypothetical protein
group_4062	1.00	0.99	1.00	hypothetical protein
group_4166	1.00	0.99	1.00	hypothetical protein
group_4221	1.00	0.99	1.00	hypothetical protein
group_4351	1.00	0.99	1.00	hypothetical protein
group_4376	1.00	0.99	1.00	hypothetical protein
group_4439	1.00	0.99	1.00	hypothetical protein
group_4457	1.00	1.00	0.99	hypothetical protein
group_4459	1.00	1.00	0.99	hypothetical protein
group_4466	1.00	0.99	1.00	hypothetical protein
group_4472	1.00	0.99	1.00	hypothetical protein
group_4516	1.00	0.99	1.00	hypothetical protein
group_4522	1.00	0.99	1.00	hypothetical protein
group_4534	1.00	0.99	1.00	hypothetical protein
group_4578	1.00	0.99	1.00	hypothetical protein
group_4579	1.00	0.99	1.00	hypothetical protein
group_4595	1.00	0.99	1.00	hypothetical protein
group_4597	1.00	0.99	1.00	hypothetical protein
group_4612	1.00	0.99	1.00	hypothetical protein
group_4647	1.00	0.99	1.00	hypothetical protein

group_4678	1.00	1.00	0.99	hypothetical protein
group_4728	1.00	0.99	1.00	hypothetical protein
group_4759	1.00	0.99	1.00	hypothetical protein
group_4760	1.00	0.99	1.00	hypothetical protein
group_4771	1.00	0.99	1.00	hypothetical protein
group_4802	1.00	0.99	1.00	hypothetical protein
group_4807	1.00	0.99	1.00	hypothetical protein
group_4844	1.00	0.99	1.00	hypothetical protein
group_4850	1.00	1.00	0.99	hypothetical protein
group_4878	1.00	0.99	1.00	hypothetical protein
group_5177	1.00	0.99	1.00	hypothetical protein
group_5250	1.00	0.99	1.00	hypothetical protein
group_5267	1.00	0.99	1.00	hypothetical protein
group_5274	1.00	1.00	0.99	hypothetical protein
group_5327	1.00	0.99	1.00	hypothetical protein
group_5387	1.00	0.99	1.00	hypothetical protein
group_5396	1.00	0.99	1.00	hypothetical protein
group_5397	1.00	0.99	1.00	hypothetical protein
group_5398	1.00	0.99	1.00	hypothetical protein
group_5408	1.00	0.99	1.00	hypothetical protein
group_5413	1.00	0.99	1.00	hypothetical protein
group_5439	1.00	0.99	1.00	hypothetical protein
group_5519	1.00	1.00	0.99	hypothetical protein
group_5536	1.00	0.99	1.00	hypothetical protein
group_5551	1.00	0.99	1.00	hypothetical protein
group_5591	1.00	0.99	1.00	hypothetical protein
group_3118	1.00	0.97	1.00	hypothetical protein
group_3784	1.00	0.97	1.00	hypothetical protein
group_4134	1.00	0.97	1.00	hypothetical protein
group_4435	1.00	0.99	0.99	hypothetical protein
group_4509	1.00	0.97	1.00	hypothetical protein
group_4557	1.00	0.97	1.00	hypothetical protein
group_4569	1.00	0.99	0.99	hypothetical protein
group_5452	1.00	0.99	0.99	hypothetical protein

group_5525	1.00	0.99	0.99	hypothetical protein
group_1508	1.00	0.96	1.00	hypothetical protein
group_2038	1.00	0.96	1.00	hypothetical protein
group_2189	1.00	0.97	0.99	hypothetical protein
group_3460	1.00	0.99	0.98	hypothetical protein
group_1826	1.00	0.96	0.99	hypothetical protein
group_711	1.00	0.99	0.96	hypothetical protein
hrp1	1.00	1.00	1.00	Hypoxic response protein 1
hisF	1.00	1.00	1.00	Imidazole glycerol phosphate synthase subunit HisF
hutI	1.00	0.99	1.00	Imidazolonepropionase
ina	1.00	1.00	1.00	Immune inhibitor A precursor
yigZ	1.00	1.00	1.00	IMPACT family member YigZ
ynjC	1.00	1.00	1.00	Inner membrane ABC transporter permease protein YnjC
yecS	1.00	1.00	1.00	Inner membrane amino-acid ABC transporter permease protein YecS
alx	1.00	1.00	1.00	Inner membrane protein alx
yccS	1.00	1.00	1.00	Inner membrane protein YccS
yhaI	1.00	1.00	1.00	Inner membrane protein YhaI
yhhQ	1.00	1.00	1.00	Inner membrane protein YhhQ
yijD	1.00	0.99	0.99	Inner membrane protein YijD
ypjD	1.00	1.00	1.00	Inner membrane protein YpjD
yqaA	1.00	1.00	1.00	Inner membrane protein YqaA
yadH	1.00	1.00	1.00	Inner membrane transport permease YadH
ydhC_1	1.00	1.00	0.99	Inner membrane transport protein YdhC
gsk_1	1.00	1.00	1.00	Inosine-guanosine kinase
ihfB	1.00	0.99	1.00	Integration host factor subunit beta
dsrF	1.00	1.00	1.00	Intracellular sulfur oxidation protein DsrF
fhuC_1	1.00	1.00	1.00	Iron(3+)-hydroxamate import ATP-binding protein FhuC
fhuC_2	1.00	0.99	1.00	Iron(3+)-hydroxamate import ATP-binding protein FhuC
iaaA	1.00	0.99	1.00	Isoaspartyl peptidase precursor
dhbC	1.00	1.00	1.00	Isochorismate synthase DhbC
ileS	1.00	0.99	1.00	Isoleucine--tRNA ligase

nhaP2_2	1.00	1.00	1.00	K(+)/H(+) antiporter NhaP2
ddc_1	1.00	1.00	1.00	L-2,4-diaminobutyrate decarboxylase
ddc_2	1.00	1.00	1.00	L-2,4-diaminobutyrate decarboxylase
lhgO	1.00	1.00	1.00	L-2-hydroxyglutarate oxidase LhgO
yfiH	1.00	1.00	1.00	Laccase domain protein YfiH
lip_1	1.00	1.00	1.00	Lactonizing lipase precursor
gloA	1.00	0.99	1.00	Lactoylglutathione lyase
ansA	1.00	1.00	1.00	L-asparaginase 1
ectC	1.00	1.00	1.00	L-ectoine synthase
lrp_2	1.00	1.00	1.00	Leucine-responsive regulatory protein
lrp_3	1.00	1.00	1.00	Leucine-responsive regulatory protein
msbB	1.00	1.00	1.00	Lipid A biosynthesis (KDO)2-(lauroyl)-lipid IVA acyltransferase
htrB	1.00	0.99	1.00	Lipid A biosynthesis lauroyl acyltransferase
msbA_1	1.00	1.00	1.00	Lipid A export ATP-binding/permease protein MsbA
msbA_2	1.00	1.00	1.00	Lipid A export ATP-binding/permease protein MsbA
ftsW	1.00	1.00	1.00	Lipid II flippase FtsW
lpxB	1.00	0.99	1.00	Lipid-A-disaccharide synthase
rfaQ	1.00	0.99	1.00	Lipopolysaccharide core heptosyltransferase RfaQ
lptF	1.00	1.00	1.00	Lipopolysaccharide export system permease protein LptF
lptA	1.00	0.99	1.00	Lipopolysaccharide export system protein LptA precursor
nlpE	1.00	1.00	1.00	Lipoprotein NlpE precursor
lspA	1.00	1.00	1.00	Lipoprotein signal peptidase
lolD_2	1.00	1.00	1.00	Lipoprotein-releasing system ATP-binding protein LolD
lolD_1	1.00	1.00	1.00	Lipoprotein-releasing system ATP-binding protein LolD
lldD	1.00	1.00	1.00	L-lactate dehydrogenase [cytochrome]
lon_3	1.00	1.00	1.00	Lon protease
lon_1	1.00	0.99	1.00	Lon protease
group_2410	1.00	1.00	1.00	Long-chain-fatty-acid--CoA ligase FadD15
pitA	1.00	0.99	0.99	Low-affinity inorganic phosphate transporter 1

lptE	1.00	1.00	1.00	LPS-assembly lipoprotein LptE precursor
ulaF	1.00	0.99	1.00	L-ribulose-5-phosphate 4-epimerase UlaF
ilvA	1.00	0.96	1.00	L-threonine dehydratase biosynthetic IlvA
cadA	1.00	1.00	1.00	Lysine decarboxylase, inducible
argT	1.00	0.99	1.00	Lysine-arginine-ornithine-binding periplasmic protein precursor
lysU	1.00	0.99	1.00	Lysine--tRNA ligase, heat inducible
macA	1.00	1.00	1.00	Macrolide export protein MacA
mdh	1.00	1.00	1.00	Malate dehydrogenase
nagL_1	1.00	1.00	1.00	Maleylpyruvate isomerase
fabD	1.00	1.00	1.00	Malonyl CoA-acyl carrier protein transacylase
bioC	1.00	1.00	1.00	Malonyl-[acyl-carrier protein] O-methyltransferase
malP	1.00	0.99	0.99	Maltodextrin phosphorylase
maa	1.00	1.00	1.00	Maltose O-acetyltransferase
mtlR	1.00	1.00	1.00	Mannitol operon repressor
mtlD	1.00	1.00	1.00	Mannitol-1-phosphate 5-dehydrogenase
yidC	1.00	0.99	1.00	Membrane protein insertase YidC
mltB	1.00	1.00	1.00	Membrane-bound lytic murein transglycosylase B precursor
mltF	1.00	1.00	1.00	Membrane-bound lytic murein transglycosylase F precursor
menF	1.00	0.99	1.00	Menaquinone-specific isochorismate synthase
metJ	1.00	0.99	1.00	Met repressor
map	1.00	1.00	1.00	Methionine aminopeptidase
mcp4_4	1.00	1.00	1.00	Methyl-accepting chemotaxis protein 4
ctpH_3	1.00	1.00	1.00	Methyl-accepting chemotaxis protein CtpH
group_1060	1.00	0.97	1.00	Methyl-accepting chemotaxis protein CtpH
pctA_1	1.00	1.00	1.00	Methyl-accepting chemotaxis protein PctA
pctB_7	1.00	1.00	1.00	Methyl-accepting chemotaxis protein PctB
pctB_1	1.00	1.00	1.00	Methyl-accepting chemotaxis protein PctB
pctC_5	1.00	1.00	1.00	Methyl-accepting chemotaxis protein PctC
pctC_4	1.00	1.00	1.00	Methyl-accepting chemotaxis protein PctC
ogt_2	1.00	1.00	1.00	Methylated-DNA--protein-cysteine methyltransferase

ogt_1	1.00	0.99	1.00	Methylated-DNA--protein-cysteine methyltransferase
cfiA	1.00	0.99	1.00	Methylmalonyl-CoA carboxyltransferase 5S subunit
hflC	1.00	1.00	1.00	Modulator of FtsH protease HflC
yccA	1.00	1.00	1.00	Modulator of FtsH protease YccA
moeA_2	1.00	1.00	1.00	Molybdopterin molybdenumtransferase
moaD	1.00	1.00	1.00	Molybdopterin synthase sulfur carrier subunit
moeB	1.00	1.00	1.00	Molybdopterin-synthase adenylyltransferase
yafQ	1.00	1.00	1.00	mRNA interferase YafQ
bmr3_2	1.00	0.99	1.00	Multidrug resistance protein 3
mdtA_2	1.00	0.97	0.99	Multidrug resistance protein MdtA precursor
mdtB	1.00	1.00	1.00	Multidrug resistance protein MdtB
mdtH	1.00	1.00	1.00	Multidrug resistance protein MdtH
norM_2	1.00	1.00	1.00	Multidrug resistance protein NorM
mepM_4	1.00	1.00	1.00	Murein DD-endopeptidase MepM
nlpD	1.00	0.97	1.00	Murein hydrolase activator NlpD precursor
mshD	1.00	1.00	1.00	Mycothiol acetyltransferase
nusB	1.00	1.00	1.00	N utilization substance protein B
chb	1.00	0.99	1.00	N,N'-diacetylchitobiase precursor
purE	1.00	1.00	1.00	N5-carboxyaminoimidazole ribonucleotide mutase
nhaD	1.00	1.00	1.00	Na(+)/H(+) antiporter NhaD
nqrA	1.00	1.00	1.00	Na(+)-translocating NADH-quinone reductase subunit A
nqrC	1.00	1.00	1.00	Na(+)-translocating NADH-quinone reductase subunit C
nqrD	1.00	1.00	1.00	Na(+)-translocating NADH-quinone reductase subunit D
nqrE	1.00	1.00	1.00	Na(+)-translocating NADH-quinone reductase subunit E
argC	1.00	1.00	1.00	N-acetyl-gamma-glutamyl-phosphate reductase
nagC_2	1.00	1.00	1.00	N-acetylglucosamine repressor
nagA_2	1.00	1.00	1.00	N-acetylglucosamine-6-phosphate deacetylase
murQ_1	1.00	1.00	1.00	N-acetylmuramic acid 6-phosphate etherase
fre	1.00	1.00	1.00	NAD(P)H-flavin reductase

ndhI	1.00	0.99	1.00	NAD(P)H-quinone oxidoreductase subunit I, chloroplastic
cobB	1.00	1.00	1.00	NAD-dependent protein deacylase
ndh	1.00	0.99	1.00	NADH dehydrogenase
nudC	1.00	1.00	1.00	NADH pyrophosphatase
sacX	1.00	0.97	1.00	Negative regulator of SacY activity
nikB	1.00	1.00	1.00	Nickel transport system permease protein NikB
nikA	1.00	0.99	1.00	Nickel-binding periplasmic protein precursor
pnuC	1.00	1.00	1.00	Nicotinamide riboside transporter PnuC
pncC	1.00	1.00	1.00	Nicotinamide-nucleotide amidohydrolase PncC
pncB	1.00	1.00	1.00	Nicotinate phosphoribosyltransferase
narX	1.00	0.99	1.00	Nitrate/nitrite sensor protein NarX
glnG	1.00	1.00	1.00	Nitrogen regulation protein NR(I)
glnL	1.00	1.00	1.00	Nitrogen regulation protein NR(II)
astD	1.00	0.99	1.00	N-succinylglutamate 5-semialdehyde dehydrogenase
sbcD	1.00	1.00	1.00	Nuclease SbcCD subunit D
slmA	1.00	0.99	1.00	Nucleoid occlusion factor SlmA
ndk	1.00	1.00	1.00	Nucleoside diphosphate kinase
nupX_2	1.00	0.99	1.00	Nucleoside permease NupX
mazG	1.00	1.00	1.00	Nucleoside triphosphate pyrophosphohydrolase
occP	1.00	1.00	1.00	Octopine permease ATP-binding protein P
prlC	1.00	1.00	1.00	Oligopeptidase A
oppD_2	1.00	1.00	1.00	Oligopeptide transport ATP-binding protein OppD
oppD_1	1.00	1.00	1.00	Oligopeptide transport ATP-binding protein OppD
oppF_1	1.00	1.00	1.00	Oligopeptide transport ATP-binding protein OppF
oppC	1.00	1.00	1.00	Oligopeptide transport system permease protein OppC
ohrR	1.00	1.00	1.00	Organic hydroperoxide resistance transcriptional regulator
argF	1.00	1.00	1.00	Ornithine carbamoyltransferase
speF	1.00	1.00	1.00	Ornithine decarboxylase, inducible
pyrF	1.00	1.00	1.00	Orotidine 5'-phosphate decarboxylase
envZ_2	1.00	0.99	1.00	Osmolarity sensor protein EnvZ

ompA	1.00	1.00	1.00	Outer membrane protein A precursor
bamB	1.00	1.00	1.00	Outer membrane protein assembly factor BamB precursor
bamC	1.00	1.00	1.00	Outer membrane protein assembly factor BamC precursor
bamE	1.00	0.99	1.00	Outer membrane protein assembly factor BamE precursor
tolC_2	1.00	0.99	1.00	Outer membrane protein TolC precursor
nreC	1.00	0.99	1.00	Oxygen regulatory protein NreC
nreB	1.00	1.00	1.00	Oxygen sensor histidine kinase NreB
hemN_1	1.00	1.00	1.00	Oxygen-independent coproporphyrinogen-III oxidase
hemN_3	1.00	1.00	1.00	Oxygen-independent coproporphyrinogen-III oxidase 1
abgT_1	1.00	1.00	1.00	p-aminobenzoyl-glutamate transport protein
panC	1.00	1.00	1.00	Pantothenate synthetase
pqiA	1.00	0.99	1.00	Paraquat-inducible protein A
lpoB	1.00	1.00	1.00	Penicillin-binding protein activator LpoB precursor
pepB	1.00	1.00	1.00	Peptidase B
pepT_2	1.00	1.00	1.00	Peptidase T
prfC	1.00	1.00	0.99	Peptide chain release factor 3
arfA_2	1.00	0.99	1.00	Peptidoglycan-binding protein ArfA
pth	1.00	1.00	1.00	Peptidyl-tRNA hydrolase
dppA_2	1.00	1.00	1.00	Periplasmic dipeptide transport protein precursor
oppA	1.00	1.00	1.00	Periplasmic oligopeptide-binding protein precursor
pspA	1.00	1.00	1.00	Phage shock protein A
phhA	1.00	0.99	1.00	Phenylalanine-4-hydroxylase
pheS	1.00	1.00	1.00	Phenylalanine--tRNA ligase alpha subunit
ybeZ_1	1.00	1.00	1.00	PhoH-like protein
ybeZ_2	1.00	1.00	1.00	PhoH-like protein
pta	1.00	0.99	1.00	Phosphate acetyltransferase
pstB	1.00	1.00	1.00	Phosphate import ATP-binding protein PstB
phoB_1	1.00	1.00	1.00	Phosphate regulon transcriptional regulatory protein PhoB

pstC_2	1.00	0.99	1.00	Phosphate transport system permease protein PstC
pstS1_1	1.00	1.00	1.00	Phosphate-binding protein PstS 1 precursor
phoU	1.00	1.00	1.00	Phosphate-specific transport system accessory protein PhoU
cdsA_2	1.00	1.00	1.00	Phosphatidate cytidylyltransferase
psd	1.00	1.00	0.99	Phosphatidylserine decarboxylase proenzyme
ppsA	1.00	1.00	0.99	Phosphoenolpyruvate synthase
ptsI_1	1.00	1.00	1.00	Phosphoenolpyruvate-protein phosphotransferase
ptsI_2	1.00	1.00	1.00	Phosphoenolpyruvate-protein phosphotransferase
pgm_1	1.00	1.00	1.00	Phosphoglucomutase
edd	1.00	1.00	1.00	Phosphogluconate dehydratase
glmM	1.00	1.00	1.00	Phosphoglucosamine mutase
pgk	1.00	1.00	1.00	Phosphoglycerate kinase
pgtC	1.00	0.99	1.00	Phosphoglycerate transport regulatory protein PgtC precursor
pgtB	1.00	0.99	1.00	Phosphoglycerate transport system sensor protein PgtB
gmhA	1.00	0.99	1.00	Phosphoheptose isomerase
sixA	1.00	1.00	1.00	Phosphohistidine phosphatase SixA
ytpA	1.00	1.00	1.00	Phospholipase YtpA
mraY	1.00	1.00	1.00	Phospho-N-acetylmuramoyl-pentapeptide-transferase
coaD	1.00	1.00	1.00	Phosphopantetheine adenylyltransferase
deoB	1.00	0.99	1.00	Phosphopentomutase
luxU	1.00	0.99	1.00	Phosphorelay protein LuxU
hisE	1.00	1.00	1.00	Phosphoribosyl-ATP pyrophosphatase
cfxP	1.00	0.99	1.00	Phosphoribulokinase, plasmid
group_3348	1.00	1.00	1.00	Phytochrome-like protein cph2
cph2_12	1.00	1.00	1.00	Phytochrome-like protein cph2
cph2_10	1.00	1.00	1.00	Phytochrome-like protein cph2
cph2_1	1.00	1.00	1.00	Phytochrome-like protein cph2
bioH_1	1.00	1.00	1.00	Pimeloyl-[acyl-carrier protein] methyl ester esterase
bioH_2	1.00	1.00	1.00	Pimeloyl-[acyl-carrier protein] methyl ester esterase
pcnB	1.00	1.00	1.00	Poly(A) polymerase I precursor

ppk	1.00	1.00	1.00	Polyphosphate kinase
priB	1.00	1.00	1.00	Primosomal replication protein n
lgt	1.00	1.00	1.00	Prolipoprotein diacylglyceryl transferase
proQ	1.00	0.99	1.00	ProP effector
ptrA_1	1.00	1.00	1.00	Protease 3 precursor
htpX	1.00	0.97	1.00	Protease HtpX
cyaY	1.00	1.00	1.00	Protein CyaY
cysZ	1.00	1.00	1.00	Protein CysZ
fdhD	1.00	1.00	1.00	Protein FdhD
slyX	1.00	0.99	1.00	Protein SlyX
smg	1.00	1.00	1.00	Protein Smg
sprT	1.00	0.99	1.00	Protein SprT
syd	1.00	1.00	1.00	Protein Syd
secD_1	1.00	1.00	1.00	Protein translocase subunit SecD
secE	1.00	1.00	1.00	Protein translocase subunit SecE
secY	1.00	1.00	1.00	Protein translocase subunit SecY
tusB	1.00	1.00	1.00	Protein TusB
secB	1.00	0.99	1.00	Protein-export protein SecB
pcm	1.00	0.99	1.00	Protein-L-isoaspartate O-methyltransferase
gltP_1	1.00	1.00	1.00	Proton glutamate symport protein
hemG	1.00	1.00	1.00	Protoporphyrinogen IX dehydrogenase [menaquinone]
mtlA	1.00	0.97	1.00	PTS system mannitol-specific EIICBA component
manP_1	1.00	1.00	1.00	PTS system mannose-specific EIIBCA component
pucG	1.00	1.00	1.00	Purine catabolism protein PucG
deoD	1.00	1.00	0.99	Purine nucleoside phosphorylase DeoD-type
deoD1	1.00	1.00	1.00	Purine nucleoside phosphorylase DeoD-type 1
yoaD	1.00	1.00	1.00	Putative 2-hydroxyacid dehydrogenase YoaD
ybhF_2	1.00	1.00	1.00	putative ABC transporter ATP-binding protein YbhF
yclQ	1.00	1.00	1.00	putative ABC transporter solute-binding protein YclQ precursor
vatD_2	1.00	0.99	1.00	Putative acetyltransferase
acuI	1.00	1.00	1.00	putative acrylyl-CoA reductase AcuI

yihG	1.00	0.96	1.00	putative acyltransferase YihG
purP	1.00	1.00	1.00	putative adenine permease PurP
yxeN	1.00	1.00	1.00	putative amino-acid permease protein YxeN
btrV	1.00	0.99	1.00	Putative anti-sigma factor antagonist BtrV
ydgI_2	1.00	1.00	1.00	Putative arginine/ornithine antiporter
ydgI_1	1.00	0.99	1.00	Putative arginine/ornithine antiporter
parB	1.00	1.00	1.00	putative chromosome-partitioning protein ParB
yfeX	1.00	1.00	1.00	putative deferrochelatase/peroxidase YfeX
adrA_8	1.00	1.00	1.00	putative diguanylate cyclase AdrA
adrA_1	1.00	1.00	1.00	putative diguanylate cyclase AdrA
ydaM_1	1.00	1.00	1.00	putative diguanylate cyclase YdaM
glnM	1.00	1.00	0.99	putative glutamine ABC transporter permease protein GlnM
engB	1.00	1.00	1.00	putative GTP-binding protein EngB
ydfH	1.00	1.00	1.00	putative HTH-type transcriptional regulator YdfH
yijE	1.00	1.00	1.00	putative inner membrane transporter yiJE
ycfS	1.00	1.00	1.00	putative L,D-transpeptidase YcfS precursor
ulaG	1.00	1.00	1.00	putative L-ascorbate-6-phosphate lactonase UlaG
ppaC	1.00	1.00	1.00	putative manganese-dependent inorganic pyrophosphatase
group_1675	1.00	1.00	1.00	Putative multidrug export ATP-binding/permease protein
hepA	1.00	0.99	1.00	Putative multidrug export ATP-binding/permease protein
emrK	1.00	1.00	1.00	putative multidrug resistance protein EmrK
tagA	1.00	0.99	1.00	Putative N-acetylmannosaminyltransferase
yjcF	1.00	1.00	1.00	putative N-acetyltransferase YjcF
group_4726	1.00	0.99	1.00	putative oxidoreductase
yceM	1.00	1.00	1.00	Putative oxidoreductase YceM
ydgJ	1.00	1.00	1.00	putative oxidoreductase YdgJ
tsaA_1	1.00	1.00	1.00	putative peroxiredoxin
bcp	1.00	1.00	1.00	Putative peroxiredoxin bcp
mlaE	1.00	1.00	1.00	putative phospholipid ABC transporter permease protein MlaE
mlaF	1.00	1.00	0.99	putative phospholipid import ATP-binding protein MlaF

sohB	1.00	0.99	1.00	putative protease SohB
psuT	1.00	1.00	1.00	Putative pseudouridine transporter
yusV	1.00	1.00	1.00	putative siderophore transport system ATP-binding protein YusV
yfiZ	1.00	1.00	1.00	putative siderophore transport system permease protein YfiZ precursor
tsaA_2	1.00	1.00	1.00	putative tRNA (adenine(37)-N6)-methyltransferase
dus	1.00	1.00	1.00	putative tRNA-dihydrouridine synthase
gspK_1	1.00	0.99	1.00	Putative type II secretion system protein K
hemX	1.00	1.00	1.00	Putative uroporphyrinogen-III C-methyltransferase
potH	1.00	1.00	1.00	Putrescine transport system permease protein PotH
potF	1.00	1.00	1.00	Putrescine-binding periplasmic protein precursor
potE	1.00	0.99	1.00	Putrescine-ornithine antiporter
yigL	1.00	1.00	1.00	Pyridoxal phosphate phosphatase YigL
pdxJ	1.00	1.00	1.00	Pyridoxine 5'-phosphate synthase
yjjG	1.00	0.99	1.00	Pyrimidine 5'-nucleotidase YjjG
sugE	1.00	1.00	1.00	Quaternary ammonium compound-resistance protein SugE
yhhW	1.00	1.00	1.00	Quercetin 2,3-dioxygenase
tgt	1.00	1.00	1.00	Queuine tRNA-ribosyltransferase
recB	1.00	0.97	1.00	RecBCD enzyme subunit RecB
recC	1.00	0.99	0.99	RecBCD enzyme subunit RecC
recD	1.00	1.00	1.00	RecBCD enzyme subunit RecD
rraA	1.00	1.00	1.00	Regulator of ribonuclease activity A
rsd	1.00	1.00	1.00	Regulator of sigma D
rseP	1.00	1.00	1.00	Regulator of sigma-E protease RseP
asnC	1.00	1.00	1.00	Regulatory protein AsnC
luxO_2	1.00	1.00	1.00	Regulatory protein LuxO
spxA	1.00	1.00	1.00	Regulatory protein Spx
pleD_4	1.00	1.00	1.00	Response regulator PleD
pleD_3	1.00	1.00	1.00	Response regulator PleD
glpG	1.00	0.99	0.99	Rhomboid protease GlpG
ribBA	1.00	0.99	1.00	Riboflavin biosynthesis protein RibBA

ribD	1.00	1.00	1.00	Riboflavin biosynthesis protein RibD
ribN	1.00	1.00	1.00	Riboflavin transporter
rnhA	1.00	0.99	1.00	Ribonuclease HI
rnhB	1.00	1.00	1.00	Ribonuclease HII
rph	1.00	0.99	1.00	Ribonuclease PH
rnt	1.00	0.99	1.00	Ribonuclease T
rbsC	1.00	0.97	1.00	Ribose transport system permease protein RbsC
rpiA	1.00	1.00	1.00	Ribose-5-phosphate isomerase A
prs	1.00	1.00	1.00	Ribose-phosphate pyrophosphokinase
rluA_1	1.00	1.00	1.00	Ribosomal large subunit pseudouridine synthase A
rluB	1.00	1.00	1.00	Ribosomal large subunit pseudouridine synthase B
rluC	1.00	1.00	1.00	Ribosomal large subunit pseudouridine synthase C
rluD_1	1.00	1.00	1.00	Ribosomal large subunit pseudouridine synthase D
rluD_2	1.00	1.00	1.00	Ribosomal large subunit pseudouridine synthase D
rluF	1.00	1.00	1.00	Ribosomal large subunit pseudouridine synthase F
rimO_3	1.00	0.99	1.00	Ribosomal protein S12 methylthiotransferase RimO
rlmG	1.00	1.00	1.00	Ribosomal RNA large subunit methyltransferase G
rlmI	1.00	0.99	1.00	Ribosomal RNA large subunit methyltransferase I
rsmD	1.00	0.99	1.00	Ribosomal RNA small subunit methyltransferase D
rsmE	1.00	1.00	1.00	Ribosomal RNA small subunit methyltransferase E
rsmH	1.00	1.00	1.00	Ribosomal RNA small subunit methyltransferase H
rsmI	1.00	0.99	1.00	Ribosomal RNA small subunit methyltransferase I
hpf	1.00	0.97	1.00	Ribosome hibernation promoting factor
rimM	1.00	1.00	1.00	Ribosome maturation factor RimM
ychF	1.00	0.97	1.00	Ribosome-binding ATPase YchF
rbfA	1.00	1.00	1.00	Ribosome-binding factor A

frr	1.00	1.00	1.00	Ribosome-recycling factor
rpe	1.00	1.00	1.00	Ribulose-phosphate 3-epimerase
fliA	1.00	1.00	0.99	RNA polymerase sigma factor FliA
rpoD	1.00	1.00	1.00	RNA polymerase sigma factor RpoD
sigV	1.00	1.00	1.00	RNA polymerase sigma factor SigV
hfq	1.00	1.00	1.00	RNA-binding protein Hfq
csrD	1.00	1.00	1.00	RNase E specificity factor CsrD
mreD	1.00	0.99	1.00	Rod shape-determining protein MreD
tatA_1	1.00	1.00	1.00	Sec-independent protein translocase protein TatA
tatB	1.00	0.99	1.00	Sec-independent protein translocase protein TatB
tatC	1.00	1.00	1.00	Sec-independent protein translocase protein TatC
yehU	1.00	0.99	1.00	Sensor histidine kinase YehU
cpxA	1.00	0.99	1.00	Sensor protein CpxA
zraS_1	1.00	1.00	1.00	Sensor protein ZraS
zraS_4	1.00	0.99	1.00	Sensor protein ZraS
sdh	1.00	1.00	1.00	Serine 3-dehydrogenase
glyA	1.00	1.00	1.00	Serine hydroxymethyltransferase
serS	1.00	0.99	1.00	Serine--tRNA ligase
aroE	1.00	1.00	1.00	Shikimate dehydrogenase
siaT_5	1.00	1.00	1.00	Sialic acid TRAP transporter permease protein SiaT
rseB	1.00	1.00	1.00	Sigma-E factor regulatory protein RseB precursor
lepB	1.00	1.00	1.00	Signal peptidase I
ffh	1.00	1.00	1.00	Signal recognition particle protein
ftsY	1.00	0.99	1.00	Signal recognition particle receptor FtsY
recJ	1.00	1.00	0.99	Single-stranded-DNA-specific exonuclease RecJ
cysG_1	1.00	1.00	1.00	Siroheme synthase
ibpA	1.00	1.00	1.00	Small heat shock protein IbpA
mscS_2	1.00	0.99	1.00	Small-conductance mechanosensitive channel
group_869	1.00	1.00	0.98	Sodium-dependent dicarboxylate transporter SdcS
slt	1.00	1.00	1.00	Soluble lytic murein transglycosylase precursor
sunS	1.00	0.99	1.00	SPBc2 prophage-derived glycosyltransferase SunS
potA_1	1.00	1.00	1.00	Spermidine/putrescine import ATP-binding protein PotA

potB	1.00	1.00	1.00	Spermidine/putrescine transport system permease protein PotB
potD_4	1.00	0.99	1.00	Spermidine/putrescine-binding periplasmic protein precursor
kinD_2	1.00	1.00	1.00	Sporulation kinase D
spo0M	1.00	0.99	1.00	Sporulation-control protein spo0M
smpB	1.00	1.00	1.00	SsrA-binding protein
spoIVFB	1.00	1.00	1.00	Stage IV sporulation protein FB
group_3961	1.00	1.00	1.00	Stearoyl-CoA 9-desaturase electron transfer partner
sspB	1.00	1.00	1.00	Stringent starvation protein B
sdhC	1.00	1.00	1.00	Succinate dehydrogenase cytochrome b556 subunit
sdhA	1.00	1.00	1.00	Succinate dehydrogenase flavoprotein subunit
sdhD	1.00	1.00	1.00	Succinate dehydrogenase hydrophobic membrane anchor subunit
sdhB	1.00	1.00	1.00	Succinate dehydrogenase iron-sulfur subunit
sucC	1.00	1.00	1.00	Succinyl-CoA ligase [ADP-forming] subunit beta
dapE	1.00	1.00	1.00	Succinyl-diaminopimelate desuccinylase
astE	1.00	1.00	1.00	Succinylglutamate desuccinylase
sfsA	1.00	1.00	1.00	Sugar fermentation stimulation protein A
cysN	1.00	1.00	0.99	Sulfate adenylyltransferase subunit 1
cysD	1.00	1.00	1.00	Sulfate adenylyltransferase subunit 2
cysT_1	1.00	0.99	1.00	Sulfate transport system permease protein CysT
cysA	1.00	1.00	1.00	Sulfate/thiosulfate import ATP-binding protein CysA
cysJ_2	1.00	1.00	1.00	Sulfite reductase [NADPH] flavoprotein alpha-component
csdE	1.00	1.00	1.00	Sulfur acceptor protein CsdE
thiF	1.00	1.00	1.00	Sulfur carrier protein ThiS adenylyltransferase
tusD	1.00	0.99	1.00	Sulfurtransferase TusD
sodC	1.00	0.97	1.00	Superoxide dismutase [Cu-Zn] precursor
sodB	1.00	1.00	1.00	Superoxide dismutase [Fe]
lacC	1.00	0.99	1.00	Tagatose-6-phosphate kinase
lpxK	1.00	1.00	1.00	Tetraacyldisaccharide 4'-kinase
gntK	1.00	0.99	1.00	Thermoresistant gluconokinase

apbE	1.00	1.00	1.00	Thiamine biosynthesis lipoprotein ApbE precursor
thiQ	1.00	0.99	1.00	Thiamine import ATP-binding protein ThiQ
thiB	1.00	0.97	0.99	Thiamine-binding periplasmic protein precursor
thiL	1.00	1.00	1.00	Thiamine-monophosphate kinase
thiE	1.00	1.00	1.00	Thiamine-phosphate synthase
thiG	1.00	0.99	1.00	Thiazole synthase
tpx	1.00	1.00	1.00	Thiol peroxidase
dsbC_2	1.00	1.00	1.00	Thiol:disulfide interchange protein DsbC precursor
glpE_1	1.00	1.00	1.00	Thiosulfate sulfurtransferase GlpE
cysP	1.00	0.99	1.00	Thiosulfate-binding protein precursor
rhtC	1.00	1.00	1.00	Threonine efflux protein
thrS	1.00	0.97	0.99	Threonine--tRNA ligase
ywlC	1.00	1.00	1.00	Threonylcarbamoyl-AMP synthase
tsaC_1	1.00	0.99	1.00	Threonylcarbamoyl-AMP synthase
deoA	1.00	0.99	1.00	Thymidine phosphorylase
thyA	1.00	1.00	1.00	Thymidylate synthase
higB.2	1.00	0.96	0.95	Toxin HigB-2
apxIB_1	1.00	1.00	1.00	Toxin RTX-I translocation ATP-binding protein
apxIB_2	1.00	0.99	1.00	Toxin RTX-I translocation ATP-binding protein
yfgC_2	1.00	0.99	1.00	TPR repeat-containing protein YfgC precursor
rfaH	1.00	1.00	1.00	Transcription antitermination protein RfaH
greA	1.00	1.00	1.00	Transcription elongation factor GreA
cadC_1	1.00	0.99	1.00	Transcriptional activator CadC
basR	1.00	1.00	1.00	Transcriptional regulatory protein BasR
citT	1.00	1.00	1.00	Transcriptional regulatory protein CitT
ompR	1.00	1.00	1.00	Transcriptional regulatory protein OmpR
uhpA	1.00	0.99	1.00	Transcriptional regulatory protein UhpA
zraR_1	1.00	0.99	1.00	Transcriptional regulatory protein ZraR
tamA	1.00	0.99	1.00	Translocation and assembly module TamA precursor
tpiA	1.00	0.99	1.00	Triosephosphate isomerase
trmL	1.00	0.99	1.00	tRNA (cytidine(34)-2'-O)-methyltransferase
trmD	1.00	1.00	1.00	tRNA (guanine-N(1)-)-methyltransferase

cmoB	1.00	1.00	1.00	tRNA (mo5U34)-methyltransferase
mnmC_2	1.00	0.99	1.00	tRNA 5-methylaminomethyl-2-thiouridine biosynthesis bifunctional protein MnmC
mnmC_1	1.00	0.99	1.00	tRNA 5-methylaminomethyl-2-thiouridine biosynthesis bifunctional protein MnmC
miaA	1.00	1.00	1.00	tRNA dimethylallyltransferase
mnmE	1.00	0.97	1.00	tRNA modification GTPase MnmE
tsaD	1.00	1.00	1.00	tRNA N6-adenosine threonylcarbamoyltransferase
truB	1.00	0.99	1.00	tRNA pseudouridine synthase B
truC	1.00	0.99	1.00	tRNA pseudouridine synthase C
tcdA	1.00	0.99	1.00	tRNA threonylcarbamoyladenosine dehydratase
tilS	1.00	0.99	1.00	tRNA(Ile)-lysidine synthase
trmA	1.00	1.00	1.00	tRNA/tmRNA (uracil-C(5))-methyltransferase
yfiC	1.00	1.00	1.00	tRNA1(Val) (adenine(37)-N6)-methyltransferase
dusC_2	1.00	1.00	1.00	tRNA-dihydrouridine synthase C
dusC_1	1.00	1.00	1.00	tRNA-dihydrouridine synthase C
tadA_1	1.00	0.99	1.00	tRNA-specific adenosine deaminase
trpB	1.00	1.00	1.00	Tryptophan synthase beta chain
ydjZ	1.00	0.99	1.00	TVP38/TMEM64 family inner membrane protein YdjZ
pilT_1	1.00	1.00	0.99	Twitching mobility protein
epsF_2	1.00	1.00	1.00	Type II secretion system protein F
epsF_1	1.00	0.99	1.00	Type II secretion system protein F
epsG	1.00	0.99	1.00	Type II secretion system protein G precursor
epsH	1.00	0.99	1.00	Type II secretion system protein H precursor
epsM	1.00	0.99	1.00	Type II secretion system protein M
pilQ	1.00	1.00	1.00	Type IV pilus biogenesis and competence protein PilQ precursor
xerC	1.00	0.99	1.00	Tyrosine recombinase XerC
xerD_1	1.00	0.99	1.00	Tyrosine recombinase XerD
tyrS_1	1.00	1.00	1.00	Tyrosine--tRNA ligase
ubiG_1	1.00	1.00	1.00	Ubiquinone biosynthesis O-methyltransferase
lpxC	1.00	1.00	1.00	UDP-3-O-[3-hydroxymyristoyl] N-acetylglucosamine deacetylase
ugd	1.00	1.00	1.00	UDP-glucose 6-dehydrogenase

wcaJ	1.00	1.00	1.00	UDP-glucose:undecaprenyl-phosphate glucose-1-phosphate transferase
murB	1.00	0.99	1.00	UDP-N-acetylenolpyruvoylglucosamine reductase
murC	1.00	0.99	1.00	UDP-N-acetylmuramate--L-alanine ligase
murF	1.00	0.97	1.00	UDP-N-acetylmuramoyl-tripeptide--D-alanyl-D-alanine ligase
uppP	1.00	1.00	1.00	Undecaprenyl-diphosphatase
uspA	1.00	0.99	1.00	Universal stress protein A
uspB	1.00	0.99	1.00	Universal stress protein B
uspF	1.00	1.00	1.00	Universal stress protein F
pyrP	1.00	1.00	1.00	Uracil permease
ung	1.00	1.00	1.00	Uracil-DNA glycosylase
udk	1.00	0.99	1.00	Uridine kinase
udp_2	1.00	1.00	1.00	Uridine phosphorylase
udp_1	1.00	1.00	1.00	Uridine phosphorylase
pyrH	1.00	1.00	1.00	Uridylate kinase
hemE	1.00	1.00	1.00	Uroporphyrinogen decarboxylase
uvrB_3	1.00	1.00	1.00	UvrABC system protein B
btuD	1.00	1.00	1.00	Vitamin B12 import ATP-binding protein BtuD
btuB_2	1.00	0.99	1.00	Vitamin B12 transporter BtuB
btuB_1	1.00	1.00	1.00	Vitamin B12 transporter BtuB precursor
gpt	1.00	1.00	1.00	Xanthine phosphoribosyltransferase
zntB	1.00	1.00	1.00	Zinc transport protein ZntB
zur	1.00	1.00	1.00	Zinc uptake regulation protein

Gene references presence in more than 95% of the lineage strains. Frequency of genes in BD-0 lineage = Freq_BD-0. Frequency of genes in BD-1 lineage = Freq_BD-1. Frequency of genes in BD-2 = Freq_BD-2. Annotation refers to gene annotation.

Table S5B. List of unique genes in BD-2

**Gene**	**Freq_BD-2**	**Annotation**
mcrB	0.942307692	5-methylcytosine-specific restriction enzyme B
dhfrI_2	0.192307692	Dihydrofolate reductase type 1
group_7033	0.971153846	hypothetical protein
group_4412	0.942307692	hypothetical protein

group_7009	0.942307692	hypothetical protein
group_7010	0.942307692	hypothetical protein
group_7011	0.942307692	hypothetical protein
group_7013	0.942307692	hypothetical protein
group_7015	0.942307692	hypothetical protein
group_7016	0.942307692	hypothetical protein
group_7017	0.942307692	hypothetical protein
group_7018	0.942307692	hypothetical protein
group_7020	0.942307692	hypothetical protein
group_7021	0.942307692	hypothetical protein
group_7019	0.932692308	hypothetical protein
group_311	0.846153846	hypothetical protein
group_5217	0.798076923	hypothetical protein
group_7022	0.798076923	hypothetical protein
group_7023	0.798076923	hypothetical protein
group_7024	0.798076923	hypothetical protein
group_7025	0.798076923	hypothetical protein
group_7026	0.798076923	hypothetical protein
group_7028	0.798076923	hypothetical protein
group_7029	0.798076923	hypothetical protein
group_7030	0.798076923	hypothetical protein
group_7027	0.788461538	hypothetical protein
group_3688	0.615384615	hypothetical protein
group_868	0.423076923	hypothetical protein
group_9986	0.221153846	hypothetical protein
group_5218	0.192307692	hypothetical protein
group_7038	0.192307692	hypothetical protein
group_7047	0.192307692	hypothetical protein
group_859	0.163461538	hypothetical protein
group_860	0.125	hypothetical protein
group_861	0.125	hypothetical protein
ctpH_4	0.653846154	Methyl-accepting chemotaxis protein CtpH
group_343	0.173076923	Microbial collagenase precursor
mrr	0.942307692	Mrr restriction system protein
group_1367	0.817307692	Phytochrome-like protein cph2

tetR	0.903846154	Tetracycline repressor protein class H
tetA	0.903846154	Tetracycline resistance protein, class B
hsdM	0.942307692	Type I restriction enzyme EcoKI M protein
hsdR_2	0.942307692	Type I restriction enzyme EcoR124II R protein
group_10030	0.596153846	Virulence protein

Gene indicates those exclusive to BD-2. Frequency of genes in BD-2 = Freq_BD-2. Annotation refers to gene annotation.

Table S5C. List of unique genes in BD-1

**Gene**	**Freq_BD-1**	**Annotation**
group_6727	0.15493	hypothetical protein
fruA_1	0.140845	PTS system fructose-specific EIIABC component
group_1838	0.098592	hypothetical protein
group_6860	0.098592	hypothetical protein
group_6861	0.098592	hypothetical protein
group_6862	0.098592	hypothetical protein
group_6863	0.098592	hypothetical protein
group_6864	0.098592	hypothetical protein
group_6866	0.098592	hypothetical protein
group_6867	0.098592	hypothetical protein
group_6868	0.098592	hypothetical protein
group_6869	0.098592	hypothetical protein
group_6870	0.098592	hypothetical protein
group_6871	0.098592	hypothetical protein
group_1703	0.070423	Oligopeptide transport ATP-binding protein OppF
group_2204	0.070423	hypothetical protein
group_1919	0.056338	Arabinose 5-phosphate isomerase KdsD
group_2258	0.056338	Methyl-accepting chemotaxis protein CtpH
group_3338	0.056338	(Dimethylallyl)adenosine tRNA methylthiotransferase MiaB

Gene indicates those exclusive to BD-1. Frequency of genes in BD-1 = Freq_BD-1. Annotation refers to gene annotation.

Table S6A. List of genes associated with BD-0 and BD-1

**Gene**	**Freq_BD-0**	**Freq_BD-1**	**P-value**	**Annotation**
nlhH	0.454545455	1	1.32E-06	Carboxylesterase NlhH

group_5718	0.272727273	0.985915	4.02E-08	hypothetical protein
pyrC	0.272727273	1	4.63E-09	Dihydroorotase
group_540	0.272727273	0.971831	1.93E-07	hypothetical protein
group_733	0.272727273	0.971831	1.93E-07	hypothetical protein
sdaC_2	0.272727273	0.985915	4.02E-08	Serine transporter
group_3309	0.272727273	0.971831	1.93E-07	hypothetical protein
group_4237	0.272727273	1	4.63E-09	hypothetical protein
group_1257	0.272727273	0.985915	4.02E-08	hypothetical protein
group_3563	0.272727273	0.985915	4.02E-08	hypothetical protein
group_2607	0	0.887324	5.40E-09	hypothetical protein
group_1292	0	0.859155	2.52E-08	hypothetical protein
group_1977	0.090909091	0.985915	5.58E-11	hypothetical protein
group_4490	1	0.028169	5.57E-12	hypothetical protein
group_2723	0.909090909	0.028169	3.31E-10	Aerotaxis receptor
group_3769	0.909090909	0.028169	3.31E-10	Murein DD-endopeptidase MepM
group_4489	0.909090909	0.028169	3.31E-10	hypothetical protein
group_5289	0.909090909	0.028169	3.31E-10	hypothetical protein
group_5290	0.909090909	0.028169	3.31E-10	hypothetical protein
tnsB	0.909090909	0.028169	3.31E-10	Transposon Tn7 transposition protein TnsB
group_5838	0.909090909	0.028169	3.31E-10	hypothetical protein
group_5074	0.818181818	0.028169	9.82E-09	hypothetical protein
group_5837	0.818181818	0.014085	1.83E-09	hypothetical protein
group_5162	0.636363636	0	8.68E-08	hypothetical protein
group_6526	0.545454545	0	1.32E-06	hypothetical protein
group_6527	0.545454545	0	1.32E-06	hypothetical protein
group_5170	0.545454545	0	1.32E-06	hypothetical protein
group_5444	1	0.028169	5.57E-12	hypothetical protein
group_2930	0.272727273	1	4.63E-09	Lon protease
group_3020	0.272727273	1	4.63E-09	hypothetical protein
group_3566	0.272727273	1	4.63E-09	hypothetical protein
group_4240	0.363636364	0.985915	6.62E-07	hypothetical protein
group_4936	0.272727273	1	4.63E-09	hypothetical protein
group_2275	0.272727273	0.985915	4.02E-08	hypothetical protein
group_3565	0.181818182	1	1.88E-10	hypothetical protein

dnaA_1	0.181818182	1	1.88E-10	Chromosomal replication initiator protein DnaA
group_5694	0.181818182	1	1.88E-10	hypothetical protein
group_5695	0.181818182	1	1.88E-10	hypothetical protein
endA	0.181818182	1	1.88E-10	Endonuclease-1 precursor
group_5699	0.181818182	1	1.88E-10	hypothetical protein
group_1747	0.181818182	0.985915	1.83E-09	hypothetical protein
group_4935	0.181818182	0.985915	1.83E-09	hypothetical protein
group_5693	0.181818182	0.985915	1.83E-09	hypothetical protein
group_5700	0.181818182	0.985915	1.83E-09	hypothetical protein
group_1752	0.181818182	0.971831	9.82E-09	hypothetical protein
group_3307	0.818181818	0.028169	9.82E-09	hypothetical protein
group_3930	1	0.028169	5.57E-12	hypothetical protein
group_4629	1	0.014085	8.57E-13	hypothetical protein
group_5445	1	0.014085	8.57E-13	hypothetical protein
rnhA_1	0.909090909	0.014085	5.58E-11	Ribonuclease HI
group_1976	0.818181818	0.014085	1.83E-09	hypothetical protein
group_2444	0.636363636	0.014085	6.62E-07	Dihydroorotase
group_5839	0.818181818	0.014085	1.83E-09	hypothetical protein
xre	0.727272727	0	4.63E-09	HTH-type transcriptional regulator Xre
group_6530	0.636363636	0	8.68E-08	hypothetical protein
group_1260	0.545454545	0	1.32E-06	hypothetical protein
recD_1	0.545454545	0	1.32E-06	RecBCD enzyme subunit RecD
group_4383	0.545454545	0	1.32E-06	hypothetical protein
group_4385	0.545454545	0	1.32E-06	hypothetical protein
group_4387	0.545454545	0	1.32E-06	hypothetical protein
group_4389	0.545454545	0	1.32E-06	hypothetical protein
group_4390	0.545454545	0	1.32E-06	Tetracycline resistance protein, class C
group_5163	0.545454545	0	1.32E-06	Regulatory protein LuxO
group_5164	0.545454545	0	1.32E-06	hypothetical protein
group_5165	0.545454545	0	1.32E-06	hypothetical protein
group_5166	0.545454545	0	1.32E-06	hypothetical protein
group_5167	0.545454545	0	1.32E-06	hypothetical protein
group_5172	0.545454545	0	1.32E-06	hypothetical protein

czcD	0.545454545	0	1.32E-06	Cadmium, cobalt and zinc/H(+)-K(+) antiporter
cls_1	0.545454545	0	1.32E-06	Cardiolipin synthase
group_6531	0.545454545	0	1.32E-06	hypothetical protein
group_6532	0.545454545	0	1.32E-06	hypothetical protein
group_6533	0.545454545	0	1.32E-06	Lon protease
group_6534	0.545454545	0	1.32E-06	hypothetical protein
group_6535	0.545454545	0	1.32E-06	hypothetical protein
group_6536	0.545454545	0	1.32E-06	hypothetical protein
group_6537	0.545454545	0	1.32E-06	hypothetical protein
group_6538	0.545454545	0	1.32E-06	hypothetical protein
group_6539	0.545454545	0	1.32E-06	hypothetical protein
group_6540	0.545454545	0	1.32E-06	hypothetical protein
group_6542	0.545454545	0	1.32E-06	hypothetical protein
group_6544	0.545454545	0	1.32E-06	hypothetical protein
group_6545	0.545454545	0	1.32E-06	hypothetical protein
group_6546	0.545454545	0	1.32E-06	hypothetical protein
group_6547	0.545454545	0	1.32E-06	hypothetical protein
group_6548	0.545454545	0	1.32E-06	hypothetical protein
group_6549	0.545454545	0	1.32E-06	hypothetical protein
group_6551	0.545454545	0	1.32E-06	hypothetical protein
group_6552	0.545454545	0	1.32E-06	hypothetical protein
group_6553	0.545454545	0	1.32E-06	hypothetical protein
group_6556	0.545454545	0	1.32E-06	hypothetical protein
group_6557	0.545454545	0	1.32E-06	hypothetical protein

Gene indicates linked to the lineage. Frequency of genes in BD-0 = Freq_BD-0. Frequency of genes in BD-1 = Freq_BD-1. P-value refers Fisher exact test p value. Annotation = gene annotation.

Table S6B. List of genes associated with BD-0 and BD-2

Gene	Freq_BD-0	Freq_BD-2	P-value	Annotation
pctC_10	0.545454545	1	3.01E-06	Methyl-accepting chemotaxis protein PctC
nlhH	0.454545455	1	1.64E-07	Carboxylesterase NlhH
group_3302	0.545454545	1	3.01E-06	hypothetical protein
group_3650	0.545454545	1	3.01E-06	hypothetical protein
group_3955	0.545454545	1	3.01E-06	hypothetical protein

group_4395	0.545454545	1	3.01E-06	hypothetical protein
group_5106	0.545454545	1	3.01E-06	hypothetical protein
group_5870	0.454545455	1	1.64E-07	hypothetical protein
group_2966	0.454545455	1	1.64E-07	hypothetical protein
group_4558	0.545454545	1	3.01E-06	hypothetical protein
swrC	0.454545455	0.99038462	1.10E-06	Swarming motility protein SwrC
pgpA	0.454545455	1	1.64E-07	Phosphatidylglycerophosphatase A
group_5821	0.454545455	0.99038462	1.10E-06	hypothetical protein
group_5860	0.545454545	1	3.01E-06	hypothetical protein
group_3047	0.454545455	0.99038462	1.10E-06	hypothetical protein
group_319	0.454545455	0.99038462	1.10E-06	hypothetical protein
higA.2	0.545454545	1	3.01E-06	Antitoxin igA-2
group_5718	0.272727273	1	2.79E-10	hypothetical protein
group_5863	0.454545455	1	1.64E-07	hypothetical protein
group_5865	0.454545455	1	1.64E-07	hypothetical protein
yiaD_2	0.545454545	1	3.01E-06	putative lipoprotein YiaD precursor
group_5869	0.454545455	1	1.64E-07	hypothetical protein
pyrC	0.272727273	0.98076923	1.19E-08	Dihydroorotase
hsdR	0.454545455	0.99038462	1.10E-06	Type-1 restriction enzyme R protein
group_540	0.272727273	0.99038462	2.45E-09	hypothetical protein
group_733	0.272727273	0.99038462	2.45E-09	hypothetical protein
sdaC_2	0.272727273	0.97115385	4.26E-08	Serine transporter
group_3309	0.272727273	0.98076923	1.19E-08	hypothetical protein
cat_1	0.545454545	1	3.01E-06	Chloramphenicol acetyltransferase
group_482	0.363636364	0.97115385	8.18E-07	putative type I restriction enzymeP M protein
group_4237	0.272727273	0.94230769	7.18E-07	hypothetical protein
group_1257	0.272727273	0.94230769	7.18E-07	hypothetical protein
group_3563	0.272727273	0.94230769	7.18E-07	hypothetical protein
group_4368	0.363636364	1	7.53E-09	hypothetical protein
group_5199	0.272727273	1	2.79E-10	hypothetical protein
group_2607	0	1	1.41E-15	hypothetical protein
group_5160	0.181818182	0.98076923	4.16E-10	hypothetical protein
group_197	0.454545455	0.99038462	1.10E-06	hypothetical protein
group_1292	0	0.92307692	1.06E-10	hypothetical protein

tktA_1	0.090909091	0.85576923	4.20E-07	Transketolase 1
luxP	0.363636364	0.99038462	5.83E-08	Autoinducer 2-binding periplasmic protein LuxP precursor
topB	0.363636364	1	7.53E-09	DNA topoisomerase 3
group_4343	0.090909091	0.99038462	1.61E-12	hypothetical protein
group_4929	0	0.98076923	1.10E-13	hypothetical protein
group_7033	0	0.97115385	5.12E-13	hypothetical protein
group_1262	0	0.94230769	1.74E-11	hypothetical protein
group_5197	0.090909091	0.94230769	1.12E-09	hypothetical protein
group_4412	0	0.94230769	1.74E-11	hypothetical protein
hsdM	0	0.94230769	1.74E-11	Type I restriction enzyme EcoKI M protein
hsdR_2	0	0.94230769	1.74E-11	Type I restriction enzyme EcoR124II R protein
group_7009	0	0.94230769	1.74E-11	hypothetical protein
group_7010	0	0.94230769	1.74E-11	hypothetical protein
group_7011	0	0.94230769	1.74E-11	hypothetical protein
mcrB	0	0.94230769	1.74E-11	5-methylcytosine-specific restriction enzyme B
group_7013	0	0.94230769	1.74E-11	hypothetical protein
mrr	0	0.94230769	1.74E-11	Mrr restriction system protein
group_7015	0	0.94230769	1.74E-11	hypothetical protein
group_7016	0	0.94230769	1.74E-11	hypothetical protein
group_7017	0	0.94230769	1.74E-11	hypothetical protein
group_7018	0	0.94230769	1.74E-11	hypothetical protein
group_7020	0	0.94230769	1.74E-11	hypothetical protein
group_7021	0	0.94230769	1.74E-11	hypothetical protein
group_7019	0	0.93269231	4.47E-11	hypothetical protein
tetA	0	0.90384615	4.96E-10	Tetracycline resistance protein, class B
tetR	0	0.90384615	4.96E-10	Tetracycline repressor protein class H
group_5208	0	0.79807692	1.81E-07	hypothetical protein
group_5209	0	0.79807692	1.81E-07	hypothetical protein
group_5210	0	0.79807692	1.81E-07	hypothetical protein
group_5211	0	0.79807692	1.81E-07	hypothetical protein
group_5212	0	0.79807692	1.81E-07	hypothetical protein
group_6872	0	0.79807692	1.81E-07	hypothetical protein
group_6873	0	0.79807692	1.81E-07	hypothetical protein
group_6875	0	0.79807692	1.81E-07	hypothetical protein

group_6876	0	0.79807692	1.81E-07	hypothetical protein
group_6877	0	0.79807692	1.81E-07	hypothetical protein
group_311	0	0.84615385	1.83E-08	hypothetical protein
group_1370	0.090909091	0.81730769	2.47E-06	Multidrug resistance protein MdtL
group_1208	0	0.73076923	2.36E-06	Hemolysin precursor
group_1367	0	0.81730769	7.68E-08	Phytochrome-like protein cph2
group_5270	0.909090909	0.04807692	4.24E-10	hypothetical protein
group_5217	0	0.79807692	1.81E-07	hypothetical protein
group_7022	0	0.79807692	1.81E-07	hypothetical protein
group_7023	0	0.79807692	1.81E-07	hypothetical protein
group_7024	0	0.79807692	1.81E-07	hypothetical protein
group_7025	0	0.79807692	1.81E-07	hypothetical protein
group_7026	0	0.79807692	1.81E-07	hypothetical protein
group_7028	0	0.79807692	1.81E-07	hypothetical protein
group_7029	0	0.79807692	1.81E-07	hypothetical protein
group_7030	0	0.79807692	1.81E-07	hypothetical protein
group_7027	0	0.78846154	2.72E-07	hypothetical protein
group_3562	0.909090909	0	1.48E-13	hypothetical protein
aldB_1	0.727272727	0.02884615	4.26E-08	Aldehyde dehydrogenase B
group_5701	0.818181818	0	7.82E-12	hypothetical protein
group_914	0.727272727	0	2.79E-10	DNA topoisomerase 3
bcr_2	0.727272727	0	2.79E-10	Bicyclomycin resistance protein
group_2363	0.727272727	0.02884615	4.26E-08	Metalloprotease StcE precursor
group_3620	0.636363636	0	7.53E-09	hypothetical protein
group_4934	0.727272727	0	2.79E-10	hypothetical protein
group_5062	0.818181818	0	7.82E-12	hypothetical protein
group_5742	0.636363636	0	7.53E-09	hypothetical protein
group_5103	0.545454545	0	1.64E-07	hypothetical protein
cynR_3	0.454545455	0	3.01E-06	HTH-type transcriptional regulator CynR
group_198	0.545454545	0.01923077	4.24E-06	hypothetical protein
group_5771	0.545454545	0.01923077	4.24E-06	hypothetical protein
group_47	0.454545455	0	3.01E-06	hypothetical protein
group_3307	0.818181818	0.01923077	4.16E-10	hypothetical protein
group_3930	1	0	1.41E-15	hypothetical protein

group_6865	0.545454545	0	1.64E-07	hypothetical protein
group_6874	0.545454545	0	1.64E-07	hypothetical protein
group_4629	1	0	1.41E-15	hypothetical protein
group_5445	1	0	1.41E-15	hypothetical protein
group_6541	0.454545455	0	3.01E-06	hypothetical protein
rnhA_1	0.909090909	0	1.48E-13	Ribonuclease HI
group_541	0.636363636	0.00961538	5.83E-08	hypothetical protein
group_1976	0.818181818	0	7.82E-12	hypothetical protein
group_2444	0.636363636	0.01923077	2.54E-07	Dihydroorotase
group_5839	0.818181818	0	7.82E-12	hypothetical protein
group_736	0.545454545	0.00961538	1.10E-06	hypothetical protein
xre	0.727272727	0	2.79E-10	HTH-type transcriptional regulator Xre
group_3869	0.454545455	0	3.01E-06	Response regulator MprA
group_6530	0.636363636	0	7.53E-09	hypothetical protein
group_1260	0.545454545	0	1.64E-07	hypothetical protein
recD_1	0.545454545	0	1.64E-07	RecBCD enzyme subunit RecD
group_4383	0.545454545	0	1.64E-07	hypothetical protein
group_4385	0.545454545	0	1.64E-07	hypothetical protein
group_4387	0.545454545	0	1.64E-07	hypothetical protein
group_4389	0.545454545	0	1.64E-07	hypothetical protein
group_4390	0.545454545	0	1.64E-07	Tetracycline resistance protein, class C
group_5163	0.545454545	0	1.64E-07	Regulatory protein LuxO
group_5164	0.545454545	0	1.64E-07	hypothetical protein
group_5165	0.545454545	0	1.64E-07	hypothetical protein
group_5166	0.545454545	0	1.64E-07	hypothetical protein
group_5167	0.545454545	0	1.64E-07	hypothetical protein
group_5172	0.545454545	0	1.64E-07	hypothetical protein
czcD	0.545454545	0	1.64E-07	Cadmium, cobalt and zinc/H(+)-K(+) antiporter
cls_1	0.545454545	0	1.64E-07	Cardiolipin synthase
group_6531	0.545454545	0	1.64E-07	hypothetical protein
group_6532	0.545454545	0	1.64E-07	hypothetical protein
group_6533	0.545454545	0	1.64E-07	Lon protease
group_6534	0.545454545	0	1.64E-07	hypothetical protein
group_6535	0.545454545	0	1.64E-07	hypothetical protein

group_6536	0.545454545	0	1.64E-07	hypothetical protein
group_6537	0.545454545	0	1.64E-07	hypothetical protein
group_6538	0.545454545	0	1.64E-07	hypothetical protein
group_6539	0.545454545	0	1.64E-07	hypothetical protein
group_6540	0.545454545	0	1.64E-07	hypothetical protein
group_6542	0.545454545	0	1.64E-07	hypothetical protein
group_6544	0.545454545	0	1.64E-07	hypothetical protein
group_6545	0.545454545	0	1.64E-07	hypothetical protein
group_6546	0.545454545	0	1.64E-07	hypothetical protein
group_6547	0.545454545	0	1.64E-07	hypothetical protein
group_6548	0.545454545	0	1.64E-07	hypothetical protein
group_6549	0.545454545	0	1.64E-07	hypothetical protein
group_6551	0.545454545	0	1.64E-07	hypothetical protein
group_6552	0.545454545	0	1.64E-07	hypothetical protein
group_6553	0.545454545	0	1.64E-07	hypothetical protein
group_6556	0.545454545	0	1.64E-07	hypothetical protein
group_6557	0.545454545	0	1.64E-07	hypothetical protein
group_1398	0.454545455	0	3.01E-06	Inner membrane transport protein YnfM
group_5174	0.454545455	0	3.01E-06	Tetracycline repressor protein class A from transposon 1721
group_6550	0.454545455	0	3.01E-06	hypothetical protein
group_6554	0.454545455	0	3.01E-06	hypothetical protein
group_6555	0.454545455	0	3.01E-06	hypothetical protein
czrA	0.454545455	0	3.01E-06	HTH-type transcriptional repressor CzrA

Here, Gene indicates linked to the lineage. Frequency of genes in BD-0 = Freq_BD-0. Frequency of genes in BD-2 = Freq_BD-2. P-value indicate Fisher exact test p value. Annotation refers to gene annotation.

Table S6C. List of genes associated with BD-1 and BD-2

**Gene**	**Freq_BD-1**	**Freq_BD-2**	**P-value**	**Annotation**
group_197	0.718309859	0.990384615	3.37E-08	hypothetical protein
group_4055	1	0.692307692	7.80E-09	hypothetical protein
group_5840	0.971830986	0.701923077	2.43E-06	hypothetical protein
group_1977	0.985915493	0.711538462	4.21E-07	hypothetical protein
group_5678	0.957746479	0.653846154	6.58E-07	hypothetical protein
group_421	0.943661972	0.653846154	2.93E-06	hypothetical protein

tar_2	0.225352113	0.990384615	1.18E-29	Methyl-accepting chemotaxis protein II
group_5822	0.126760563	0.990384615	2.70E-36	hypothetical protein
group_4490	0.028169014	0.990384615	2.98E-45	hypothetical protein
luxP	0.112676056	0.990384615	2.20E-37	Autoinducer 2-binding periplasmic protein LuxP precursor
group_2723	0.028169014	0.990384615	2.98E-45	Aerotaxis receptor
group_3769	0.028169014	0.990384615	2.98E-45	Murein DD-endopeptidase MepM
group_4489	0.028169014	0.990384615	2.98E-45	hypothetical protein
group_5289	0.028169014	0.990384615	2.98E-45	hypothetical protein
group_5290	0.028169014	0.990384615	2.98E-45	hypothetical protein
tnsB	0.028169014	0.990384615	2.98E-45	Transposon Tn7 transposition protein TnsB
group_5838	0.028169014	0.990384615	2.98E-45	hypothetical protein
group_5074	0.028169014	0.990384615	2.98E-45	hypothetical protein
group_1756	0.985915493	0.336538462	5.99E-21	Methyl-accepting chemotaxis protein CtpH
yihX	0.985915493	0.326923077	1.80E-21	Alpha-D-glucose-1-phosphate phosphatase YihX
group_5837	0.014084507	0.990384615	5.75E-47	hypothetical protein
topB	0.014084507	1	8.18E-49	DNA topoisomerase 3
group_1726	0.943661972	0.326923077	8.52E-18	Prophage CP4-57 integrase
group_4343	0.028169014	0.990384615	2.98E-45	hypothetical protein
group_4929	0.056338028	0.980769231	9.09E-41	hypothetical protein
group_77	0.915492958	0.317307692	2.76E-16	hypothetical protein
group_3686	0.943661972	0.298076923	3.84E-19	hypothetical protein
group_5162	0	0.942307692	1.85E-42	hypothetical protein
group_6526	0	0.942307692	1.85E-42	hypothetical protein
group_6527	0	0.942307692	1.85E-42	hypothetical protein
group_5161	0	0.942307692	1.85E-42	hypothetical protein
group_6528	0	0.942307692	1.85E-42	hypothetical protein
group_7033	0	0.971153846	5.05E-46	hypothetical protein
group_1262	0.014084507	0.942307692	1.69E-40	hypothetical protein
group_5197	0	0.942307692	1.85E-42	hypothetical protein
mdtL_2	0.985915493	0.182692308	7.68E-30	Multidrug resistance protein MdtL
group_4412	0	0.942307692	1.85E-42	hypothetical protein
hsdM	0	0.942307692	1.85E-42	Type I restriction enzyme EcoKI M protein
hsdR_2	0	0.942307692	1.85E-42	Type I restriction enzyme EcoR124II R protein

group_7009	0	0.942307692	1.85E-42	hypothetical protein
group_7010	0	0.942307692	1.85E-42	hypothetical protein
group_7011	0	0.942307692	1.85E-42	hypothetical protein
mcrB	0	0.942307692	1.85E-42	5-methylcytosine-specific restriction enzyme B
group_7013	0	0.942307692	1.85E-42	hypothetical protein
mrr	0	0.942307692	1.85E-42	Mrr restriction system protein
group_7015	0	0.942307692	1.85E-42	hypothetical protein
group_7016	0	0.942307692	1.85E-42	hypothetical protein
group_7017	0	0.942307692	1.85E-42	hypothetical protein
group_7018	0	0.942307692	1.85E-42	hypothetical protein
group_7020	0	0.942307692	1.85E-42	hypothetical protein
group_7021	0	0.942307692	1.85E-42	hypothetical protein
dauA	0.971830986	0.182692308	3.19E-28	C4-dicarboxylic acid transporter DauA
group_7019	0	0.932692308	2.06E-41	hypothetical protein
group_5169	0.098591549	0.798076923	3.75E-21	hypothetical protein
group_2538	0.957746479	0.182692308	6.29E-27	hypothetical protein
tetA	0	0.903846154	1.46E-38	Tetracycline resistance protein, class B
tetR	0	0.903846154	1.46E-38	Tetracycline repressor protein class H
group_2509	0.014084507	0.855769231	1.26E-32	hypothetical protein
group_2537	0.042253521	0.817307692	6.29E-27	hypothetical protein
group_5208	0.098591549	0.798076923	3.75E-21	hypothetical protein
group_5209	0.098591549	0.798076923	3.75E-21	hypothetical protein
group_5210	0.098591549	0.798076923	3.75E-21	hypothetical protein
group_5211	0.098591549	0.798076923	3.75E-21	hypothetical protein
group_5212	0.098591549	0.798076923	3.75E-21	hypothetical protein
group_6872	0.098591549	0.798076923	3.75E-21	hypothetical protein
group_6873	0.098591549	0.798076923	3.75E-21	hypothetical protein
group_6875	0.098591549	0.798076923	3.75E-21	hypothetical protein
group_6876	0.098591549	0.798076923	3.75E-21	hypothetical protein
group_6877	0.098591549	0.798076923	3.75E-21	hypothetical protein
group_2143	0.028169014	0.817307692	3.19E-28	C4-dicarboxylic acid transporter DauA
group_5170	0	0.798076923	2.32E-30	hypothetical protein
group_5444	0.028169014	0.730769231	4.86E-23	hypothetical protein
group_311	0	0.846153846	9.23E-34	hypothetical protein

group_5171	0	0.798076923	2.32E-30	hypothetical protein
group_6543	0	0.798076923	2.32E-30	hypothetical protein
group_1370	0.014084507	0.817307692	7.68E-30	Multidrug resistance protein MdtL
group_5168	0	0.798076923	2.32E-30	hypothetical protein
group_1208	0.126760563	0.730769231	5.65E-16	Hemolysin precursor
group_1367	0	0.817307692	1.25E-31	Phytochrome-like protein cph2
group_5270	0.971830986	0.048076923	6.35E-40	hypothetical protein
group_5217	0	0.798076923	2.32E-30	hypothetical protein
group_7022	0	0.798076923	2.32E-30	hypothetical protein
group_7023	0	0.798076923	2.32E-30	hypothetical protein
group_7024	0	0.798076923	2.32E-30	hypothetical protein
group_7025	0	0.798076923	2.32E-30	hypothetical protein
group_7026	0	0.798076923	2.32E-30	hypothetical protein
group_7028	0	0.798076923	2.32E-30	hypothetical protein
group_7029	0	0.798076923	2.32E-30	hypothetical protein
group_7030	0	0.798076923	2.32E-30	hypothetical protein
group_7027	0	0.788461538	1.17E-29	hypothetical protein
group_3562	1	0	7.79E-51	hypothetical protein
aldB_1	0.985915493	0.028846154	4.94E-44	Aldehyde dehydrogenase B
group_5701	0.985915493	0	8.18E-49	hypothetical protein
group_1724	0.042253521	0.673076923	7.60E-19	Prophage CP4-57 integrase
group_914	0.985915493	0	8.18E-49	DNA topoisomerase 3
bcr_2	0.971830986	0	4.33E-47	Bicyclomycin resistance protein
group_2363	0.929577465	0.028846154	4.15E-38	Metalloprotease StcE precursor
group_3620	0.985915493	0	8.18E-49	hypothetical protein
group_4934	0.971830986	0	4.33E-47	hypothetical protein
group_5062	0.957746479	0	1.55E-45	hypothetical protein
group_5742	0.985915493	0	8.18E-49	hypothetical protein
group_4235	0.718309859	0.230769231	1.85E-10	hypothetical protein
group_4475	0.732394366	0.153846154	9.36E-15	hypothetical protein
group_2336	0.014084507	0.692307692	2.97E-22	hypothetical protein
group_2930	1	0	7.79E-51	Lon protease
group_3020	1	0	7.79E-51	hypothetical protein
group_3566	1	0	7.79E-51	hypothetical protein

group_4240	0.985915493	0	8.18E-49	hypothetical protein
group_4936	1	0	7.79E-51	hypothetical protein
group_5103	0.957746479	0	1.55E-45	hypothetical protein
group_2275	0.985915493	0	8.18E-49	hypothetical protein
group_2512	0.028169014	0.673076923	6.54E-20	Alpha-D-glucose-1-phosphate phosphatase YihX
group_2935	0.028169014	0.653846154	3.59E-19	hypothetical protein
group_3565	1	0	7.79E-51	hypothetical protein
dnaA_1	1	0	7.79E-51	Chromosomal replication initiator protein DnaA
group_5694	1	0	7.79E-51	hypothetical protein
group_5695	1	0	7.79E-51	hypothetical protein
endA	1	0	7.79E-51	Endonuclease-1 precursor
group_5699	1	0	7.79E-51	hypothetical protein
group_1747	0.985915493	0	8.18E-49	hypothetical protein
group_4935	0.985915493	0	8.18E-49	hypothetical protein
group_5693	0.985915493	0	8.18E-49	hypothetical protein
group_5700	0.985915493	0	8.18E-49	hypothetical protein
group_1752	0.971830986	0	4.33E-47	hypothetical protein
cynR_3	0.929577465	0	9.10E-43	HTH-type transcriptional regulator CynR
group_76	0.070422535	0.615384615	4.60E-14	hypothetical protein
ctpH_4	0	0.653846154	3.44E-22	Methyl-accepting chemotaxis protein CtpH
group_2633	0.901408451	0	2.65E-40	Autoinducer 2-binding periplasmic protein LuxP precursor
group_316	0.845070423	0.009615385	3.06E-34	hypothetical protein
group_3688	0	0.615384615	2.16E-20	hypothetical protein
group_10030	0	0.596153846	2.00E-19	Virulence protein
group_1140	0.774647887	0.009615385	1.18E-29	Methyl-accepting chemotaxis protein II
group_3587	0.704225352	0.086538462	9.14E-18	hypothetical protein
group_313	0.084507042	0.471153846	2.12E-08	hypothetical protein
group_194	0.76056338	0	1.72E-30	Chemotaxis protein CheA
group_4931	0.647887324	0.009615385	9.24E-23	hypothetical protein
group_868	0	0.423076923	1.53E-12	hypothetical protein
group_2369	0.014084507	0.394230769	3.03E-10	hypothetical protein
group_9987	0.436619718	0.067307692	1.04E-08	hypothetical protein

group_6707	0	0.298076923	1.72E-08	hypothetical protein
group_198	0.253521127	0.019230769	1.82E-06	hypothetical protein
group_9986	0	0.221153846	3.40E-06	hypothetical protein
arcA_3	0.23943662	0.009615385	7.40E-07	Arginine deiminase

Gene indicates linked to the lineage. Frequency of genes in BD-1 = Freq_BD-1. Frequency in BD-2 = Freq_BD-2. P-value indicates Fisher exact test p value. Annotation refers to gene annotation.

Table S7. Number of strains with phage inducible chromosomal island like elements (PLE)

Lineage	PLE(-)	PLE1	PLE2
BD-0	11	0	0
BD-1	66	0	10
BD-2	22	83	0

Absence of PLE = PLE(-), and PLE1 and PLE2 are two different types of PLE.

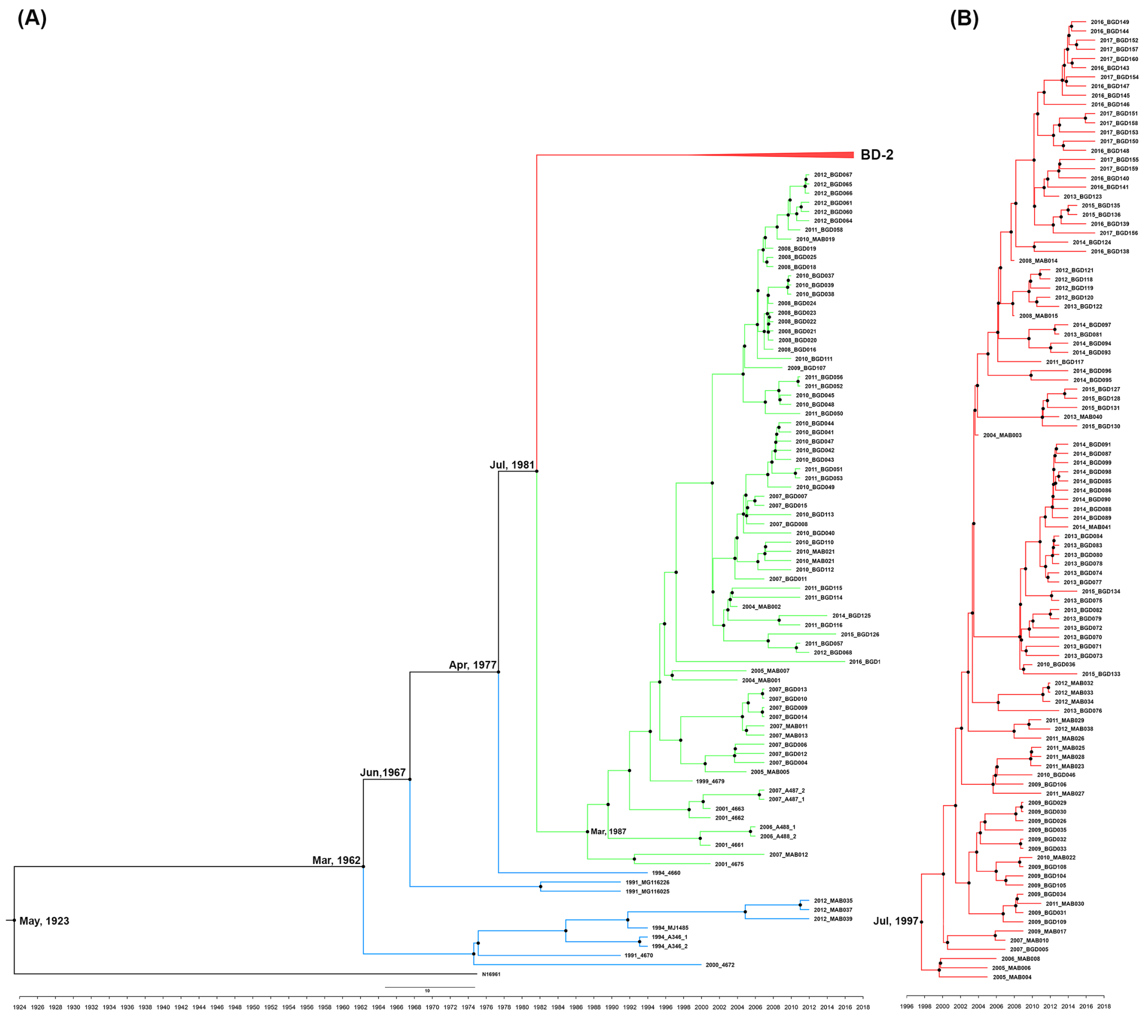

FIG S1 Bayesian phylogenetic analysis of *V. cholerae* O1. Node ages obtained from BEAST analysis. Tree visualized using FigTree v1.4.4. Colors of clades reference the lineage.

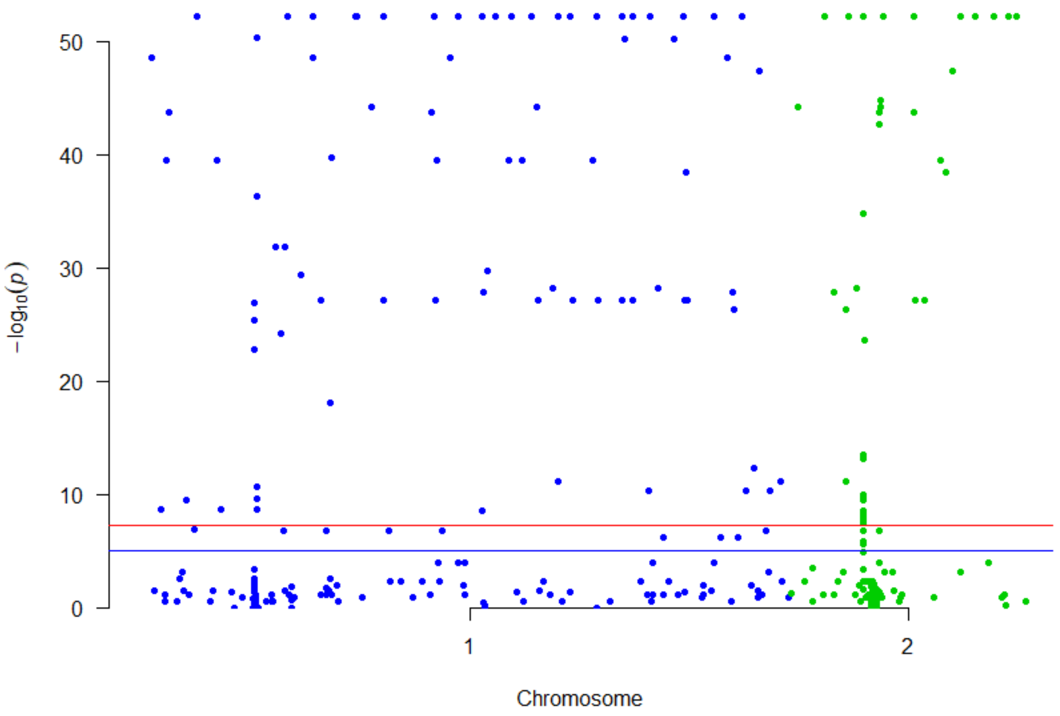

FIG S2 Manhattan plots of *p*-values for association studies of SNPs and BD-1 and BD-2 lineages. Blue represent suggested significant and red indicates high significance. Association analysis reveals 140 SNP difference between BD-1 and BD-2 lineages.

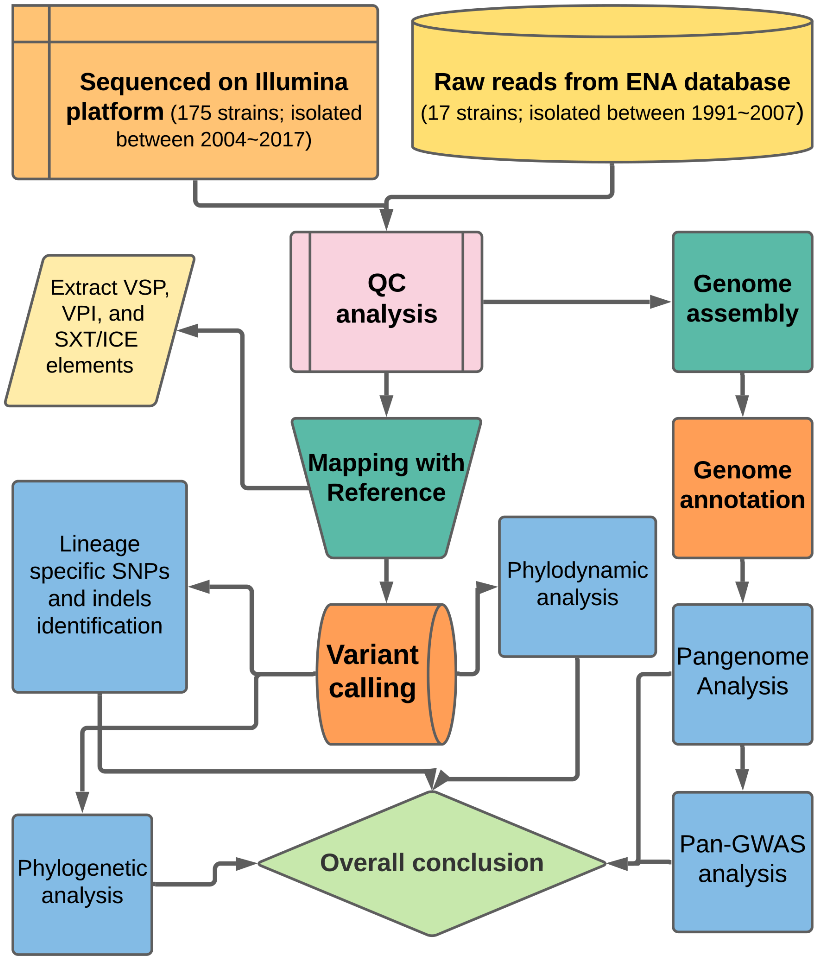

FIG S3 Study flow chart. Data curation and analyses steps are given in the flow chart.
